# Assessing the Association of Element Imbalances With Arsenism and the Potential Application Value of *Rosa roxburghii* Tratt Juice

**DOI:** 10.3389/fphar.2022.819472

**Published:** 2022-04-25

**Authors:** Yuyan Xu, Baofei Sun, Qibing Zeng, Shaofeng Wei, Guanghong Yang, Aihua Zhang

**Affiliations:** The Key Laboratory of Environmental Pollution Monitoring and Disease Control, Ministry of Education & School of Public Health, Guizhou Medical University, Guiyang, China

**Keywords:** arsenism, *Rosa roxburghii* Tratt, element imbalance, cross-sectional study, intervention study

## Abstract

Endemic arsenism caused by coal burning is a unique type of biogeochemical disease that only exists in China, and it is also a disease of element imbalances. Previous studies have shown that element imbalances are involved in the pathogenesis of arsenic; however, the interaction between the various elements and effective preventive measures have not been fully studied. This study first conducted a cross-sectional study of a total of 365 participants. The results showed that arsenic exposure can increase the content of elements (Al, As, Fe, Hg, K, and Na) in the hair (*p <* 0.05), but the content of other elements (Ca, Co, Cu, Mn, Mo, P, Se, Sr, V, and Zn) was significantly decreased (*p <* 0.05). Also, the high level of As, Fe, and Pb and the low level of Se can increase the risk of arsenism (*p <* 0.05). Further study found that the combined exposure of Fe–As and Pb–As can increase the risk of arsenism, but the combined exposure of Se–As can reduce the risk of arsenism (*p <* 0.05). In particular, a randomized, controlled, double-blind intervention study reveals that *Rosa roxburghii* Tratt juice (*RRT*) can reverse the abovementioned element imbalances (the high level of Al, As, and Fe and the low level of Cu, Mn, Se, Sr, and Zn) caused by arsenic (*p <* 0.05). Our study provides some limited evidence that the element imbalances (the high level of As, Fe, and Pb and the low level of Se) are the risk factors for the occurrences of arsenism. The second major finding was that *RRT* can regulate the element imbalances, which is expected to improve arsenism. This study provides a scientific basis for further understanding a possible traditional Chinese health food, *RRT*, as a more effective detoxication of arsenism.

## Introduction

Arsenic is a major metabolic poison and is widely distributed in virtually all rocks and sediments, aqueous environments, air, soil, food, and other natural and geogenic environments (Sarkar and Paul, 2016; Doerge et al., 2020; Podgorski and Berg, 2020). The important route of inorganic arsenic exposure is through the high-arsenic-contaminated water worldwide (Rodríguez-Lado et al., 2013; Podgorski and Berg, 2020), such as in the Bengal delta (Kippler et al., 2016), India (Chakraborti et al., 2016; Kumar et al., 2016), Pakistan (Podgorski et al., 2017), Mexico (Alarcón-Herrera et al., 2013), Argentina (Bardach et al., 2015), Vietnam (Winkel et al., 2011), Cambodia (Buschmann et al., 2007), the United States (Ayotte et al., 2017), and China (Rodríguez-Lado et al., 2013; Zhou et al., 2017). However, exposure to an arsenic-contaminated diet (such as chili peppers, corn, and rice, which are very popular in Guizhou Province, China) and air via the burning of high-arsenic coal in unventilated indoor stoves is unique to China. A large-scale census study consisting of 4763 samples found that the average arsenic content of Chinese coal is 3.18 ppm in weight (Kang et al., 2011). Especially in the southwest region of China, the level of coal arsenic content was as high as 35,000 ppm in Guizhou Province, 20 years ago (Finkelman et al., 1999). With the government’s commitment to mobilize the resources of the whole society, the prevention and control of endemic diseases have been promoted and remarkable progress has been achieved (Wang et al., 2019). In particular, with the implementation of comprehensive intervention measures such as improving stoves and health education, the total arsenic level in Guizhou areas has significantly decreased (Wang et al., 2019), but the health hazards in endemic arsenism have the characteristics of accumulation and irreversibility. Furthermore, the pathogenic mechanism of arsenism is unclear and there are no targeted effective treatment drugs and other factors, which have become the bottleneck restricting the continuous control and elimination of the disease. Currently, exposure to arsenic-contaminated diet and air remains a major environmental public health concern in Guizhou Province, China.

Trace elements (Al, As, B, Cd, Co, Cr, Cu, Fe, Hg, Li, Mn, Mo, Ni, Pb, Se, Sr, V, and Zn) are minerals with small amounts (less than 0.01% of body weight) in the human body, compared with constant elements (such as Ca, K, Mg, Na, and P) (Zhang et al., 2018; Xu et al., 2021a). Although most trace elements are essential to human health, excessive concentrations of trace elements can also be toxic to the body (Zeng et al., 2021; Zheng et al., 2021). Growing evidence from animal and human studies (Wei et al., 2018a; Wei et al., 2018b; Li et al., 2019a; Hu et al., 2020; Outa et al., 2020; Hu et al., 2021; Çiner et al., 2021) indicates that arsenism is a disease with element imbalances, which is related to the uneven distribution of elements in the environment and the element imbalances in individuals exposed to arsenic. However, the results of the aforementioned studies are not consistent, and the element imbalances in arsenic-exposed people still need to be fully examined. In particular, from the perspective of multi-element interaction to explore the role of element imbalances in the pathogenesis of arsenic, it is expected to deepen the understanding of the pathogenicity of arsenism from a new perspective.

For thousands of years, humans have relied on plants as food and to alleviate diseases (Sen and Samanta, 2015). Natural medicinal and edible plants have contributed extensively towards the development of modern medicine (Kushiro et al., 2003); however, a large number of natural products are yet to be developed and applied (Sen and Samanta, 2015). *Rosa roxburghii* Tratt (*RRT*) juice is the original juice of *Rosa roxburghii* Tratt fruit, which is a traditional Chinese health food that is unique to the mountainous area of southwest China (Shi et al., 2020). *RRT* contains a variety of biologically active metabolites (such as pentacyclic triterpenoids and flavonoids) and rich nutrients (including trace elements, vitamins, polysaccharides, dietary fiber, unsaturated fatty acids, and superoxide dismutase) (Wu et al., 2020). Our previous animal study found that *RRT* can attenuate liver damage in arsenic-poisoned rats by regulating element balance and oxidative stress (Xu et al., 2021b). However, very little is known about the interventional effects of *RRT* in arsenism populations. Since there is a significant correlation between element imbalances and arsenism, the *RRT* has been found in animal experiments to regulate the balance of elements. Therefore, based on population intervention studies, it is explored whether *RRT* can improve arsenism and its possible mechanism is useful for better explaining the role of *RRT* in arsenism and it has important scientific significance and transformational application value.

In this study, we first analyzed the association between arsenism and the content of various trace elements (Al, As, B, Cd, Co, Cr, Cu, Fe, Hg, Li, Mn, Mo, Ni, Pb, Se, Sr, V, and Zn) and constant elements (Ca, K, Mg, Na, and P) in the hair through a cross-sectional study. Second, three logistic regression models were used to explore the risk of arsenism caused by the changes of the aforementioned elements and their interactions. Finally, a randomized, controlled, double-blind intervention study of *RRT* lasting 3 months was designed. By observing the changes in the aforementioned trace elements and constant elements, the aim was to study the role and potential mechanism of *RRT* in the detoxication of arsenism. This study will help to better understand the mechanisms of arsenism, and our results will be used to find one more effective health food to detoxify arsenism.

## Materials and Methods

### Study Population

As described in our previous study (Zeng et al., 2019), the Jiaole village and Daguoduo village, Xingren County, Guizhou Province, China, were chosen as the arsenic-exposed area and arsenic-free sites, according to the “Definition and Division Standard for Endemic Arsenism” (WS277-2007, Ministry of Health of the People’s Republic of China) and referring to the World Health Organization (WHO) safety standard of 10 μg/L. We worked with the original 44th Hospital of the Chinese People’s Liberation Army to recruit volunteers. A total of 365 participants in the cross-sectional study were selected by the cluster random sampling method, including an arsenism group of 311 cases and a reference group of 54 cases. The inclusion criterion is that all participants must be permanent residents of Jiaole village and Daguoduo village. Also, the exclusion criteria included the recent history of consumption of seafood and drugs (such as trace elemental supplements), which may affect the metabolism of elements.

A randomized, placebo-controlled, double-blind, parallel trial was applied in this study. *RRT* is the original juice of *Rosa roxburghii* Tratt [Rosaceae] fruit, which was purchased from Sinopharm Group Guizhou Healthcare Industry Development Co., Ltd. [the health food permission number of the National Health Commission of the People’s Republic of China is (2002)0004]; placebo was produced by Sinopharm Group Guizhou Healthcare Industry Development Co., Ltd., and the physical characteristics (such as appearance, size, color, dosage form, weight, taste, and smell) are the same as those of *RRT*, but the main component is glucose. According to the block random design, a total of 92 participants (aged 30–65 years) were divided into different block groups according to age (an interval every 5 years) and gender; the same block group was equally distributed to the *RRT* group (46 cases) and the placebo group (46 cases) according to the principle of randomization. The inclusion and exclusion criteria are the same as in the cross-sectional study except for age restrictions. Oral administration was used, with a dose of 20 ml each day (according to the recommended dosage in the health food instructions), once a day in the morning after breakfast for 90 days. During the entire cycle of the intervention, we conducted regular monitoring and made daily records, including the food intake and intake habits of the participants. We, especially, strictly controlled the intake of seafood and trace element supplements. To ensure the compliance and reliability of *RRT* intervention across the study, all patients were controlled through telephonic follow-up and on-site supervision was arranged every day. Finally, 84 participants completed the standard full-course intervention, 42 cases per group.

Both the cross-sectional study and the intervention study were approved by the Ethics Committee of Guizhou Medical University (No. 201403001). Also, written informed consent was obtained from each participant.

### Arsenism Diagnosis

According to the “Diagnostic Standards for Endemic Arsenic Poisoning” (WS/T 211-2015, Ministry of Health of the People’s Republic of China) and referring to the WHO diagnostic criteria for chronic arsenic poisoning (Organization, 2000), residents living in endemic arsenism areas with a history of excessive arsenic exposure (the hair arsenic content was significantly higher than the reference value of the non-arsenism area) and meeting one of the following clinical characteristics can be diagnosed as endemic arsenism: 1) the palm and plantar skin has other reasons that are difficult to explain, papule-like, nodular or verrucous hyperkeratosis; 2) the skin on the non-exposed part of the torso has diffuse or scattered spot-like pigmentation that is difficult to explain for other reasons and/or the mesh-shaped pigment loss spots with fuzzy edges, and the size ranges from millet grains to soybean grains. Dermatologists qualified for the diagnosis of arsenism are employed for the disease diagnosis of all participants. These dermatologists have more than 20 years of clinical experience. All participants undergo a double diagnosis and review. When the doctors’ diagnosis conclusions are inconsistent, another doctor will be invited to conduct a review.

### Hair Collection and Pretreatment

Under the principle of informed consent, we collected all participants’ hair behind the occiput (close to the hair root within 2 cm) for two periods of time before and after the intervention. We put the hair sample into the sample tube and washed the hair sample with acetone (Merck, Germany), deionized water, deionized water, and acetone in sequence. After drying, we used stainless steel scissors to cut the hair into approximately 1 mm segments. We weighed two portions of 20 mg hair into a sample tube and added 800 µL of 65% concentrated nitric acid (Merck, Germany) and 200 µL of 30% hydrogen peroxide (Merck, Germany), respectively. It was allowed to stand for 10 min in a sealed state, and then, the sample tube was placed in a dry thermostat and heated to 90°C for heating and digestion for 3 h. After the digestion was completed, we let the digestion solution drop to room temperature, unscrewed the cap in a fume hood, transfered the digestion solution to a volumetric flask, rinsed the sample tube three times with a little deionized water, poured it into a 10 ml volumetric flask, and continued adding the diluted volume of deionized water to the mark, until analysis.

### Element Determination

The determination of 23 elements in the hair is similar to that described in previous studies (Li et al., 2019b; Yang et al., 2019). In short, the inductively coupled plasma mass spectrometer (Avio 200, PerkinElmer, United States) is used for the determination of the aforementioned elements in the hair. All samples are measured in random order. To ensure the accuracy and reliability of the data, internal and external standard methods are used for quality control in the measurement process. After we measure every 20 samples, we will add a trace element quality control sample (No. 8883 and 8884, Recipe, Germany). When the element content in the sample is below the detection limit, we estimate the element content as half of the detection limit.

### Statistical Analysis

R for Windows version 4.03 software is used for frequency, median, and interquartile range calculations, the *t*-test, the chi-squared test, the median test, analysis of variance, and logistic regression analysis. Independent-sample *t*-tests were used to compare the differences in age between the two groups, and the data were expressed as mean ± SD. We performed rate (such as gender, smoking status, drinking alcohol, and arsenism) comparisons using the chi-squared test. The median test was used to compare the content of 23 elements in the hair between the various groups, and the median and interquartile ranges were expressed in the results. For the association and risk of the aforementioned elements and the incidence of arsenism, we used three logistic regression models. First, model 1 (univariate logistic regression) was used to determine the relationship between 23 elements, effect factors (including age, gender, smoking, and drinking), and arsenism. Then, the statistically significant elements in model 1 analysis along with age, gender, smoking status, and drinking alcohol status are placed into the model together; model 2 (multivariate logistic regression) was applied to analyze the independent factors associated with arsenism and estimate its risks. Finally, the statistically significant elements in model 2 analysis are placed into the model together, after adjusting for age, gender, smoking, and drinking; and model 3 (interaction logistic regression) was used to analyze the interaction between the elements and arsenism and estimate its risks. The criterion for a significant difference was *p <* 0.05.

## Results

### Characteristics of the Study Participants

Table 1 shows the characteristics of the study participants, and the age and the prevalence of arsenism in the arsenic exposure group are higher than those in the reference group (*T = −*6.51, *p <* 0.01; *χ* ^*2*^ *=* 127.80; *p <* 0.01). Also, there are significant differences in the gender composition ratios between the reference and arsenic exposure groups (*χ* ^*2*^ *=* 12.30, *p <* 0.01). However, there is no significant difference between the smoking status and drinking alcohol status (*χ* ^*2*^ *=* 5.05, 4.90; *p =* 0.80, 0.09).

**TABLE 1:** Characteristics of the study participants (*n =* 365).

Characteristics	Reference	Arsenic exposure	Statistical value	*p* value
Age	40.57 ± 9.65	50.38 ± 10.32	−6.5^a^	<0.01
Gender			12.30^b^	<0.01
Male	13 (24.07%)	155 (49.84%)		
Female	41 (75.93%)	156 (50.16%)		
Smoking status			5.05^b^	0.80
Never smoking	40 (74.07%)	186 (59.81%)		
Even smoking	13 (24.07%)	99 (31.83%)		
Now smoking	1 (1.85%)	26 (8.36%)		
Drinking alcohol status			4.90^b^	0.09
Never drinking	44 (81.48%)	220 (70.74%)		
Even drinking	9 (16.67%)	56 (18.01%)		
Now drinking	1 (1.85%)	35 (11.25%)		
Arsenism			127.80^b^	<0.01
Yes	0 (0.00%)	244 (78.46%)		
No	54 (100.00%)	67 (21.54%)		

a *t*-test, the statistical value is *t* value, and the data were presented as mean ± standard deviation.

b Chi-square test, the statistical value is *χ* ^*2*^ value, and the data were presented as numbers (percentage).

### Association and Risk of Element Imbalances With Arsenism

#### Effects of Arsenic Exposure in the Balance of Elements

**FIGURE 1:**
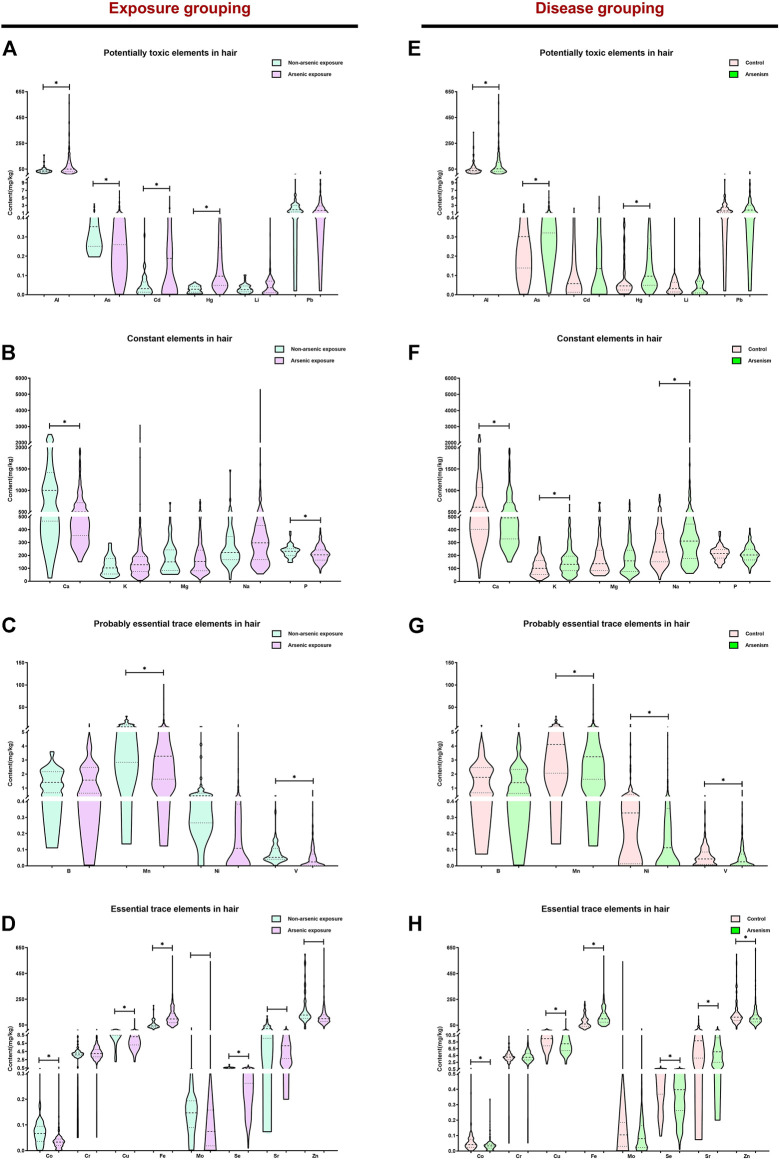
The content of 23 elements in the hair of different groups. In this study, the median and interquartile range were expressed in the results, *p < 0.05. Based on exposure grouping, **(A–D)** the content of potentially toxic elements, constant elements, probably essential trace elements, and essential trace elements in different groups, respectively, is shown. Subsequently, based on disease grouping, the differences of potentially toxic elements, constant elements, probably essential trace elements, and essential trace elements in the two groups are clearly shown **(E–H)**.

Hair arsenic is useful as an exposure biomarker, reflecting the arsenic intake of the chronic arsenism population (Hindmarsh, 2002). To study the effects of arsenic exposure in the balance of elements, the content of 23 elements in hair was determined. Figures 1A–D clearly show that the content of the potentially toxic elements (Al, As, Cd, and Hg) and the essential trace element Fe in the arsenic exposure group is higher than that of the non-arsenic exposure group (*p <* 0.05). Also, the content of constant elements (Ca and P), probably essential trace elements (Mn and V), and essential trace elements (Co, Cu, Mo, Se, Sr, and Zn) was significantly lower than that in the non-arsenic exposure group (*p <* 0.05). Figures 1E–H show the differences in the content of 23 elements in the hair of different groups. For the arsenism group, the content of the potentially toxic elements (Al, As, and Hg), constant elements (K and Na), and essential trace element (Fe) gradually increased, and the content of constant element Ca, probably essential trace elements (Mn, Ni, and V), and essential trace elements (Co, Cu, Se, Sr, and Zn) gradually decreased compared with that in the control group (*p <* 0.05). However, there is no significant difference among other elements in the different groups (*p >* 0.05).

#### Association and Risk Between the Element Imbalances and Arsenism

**FIGURE 2:**
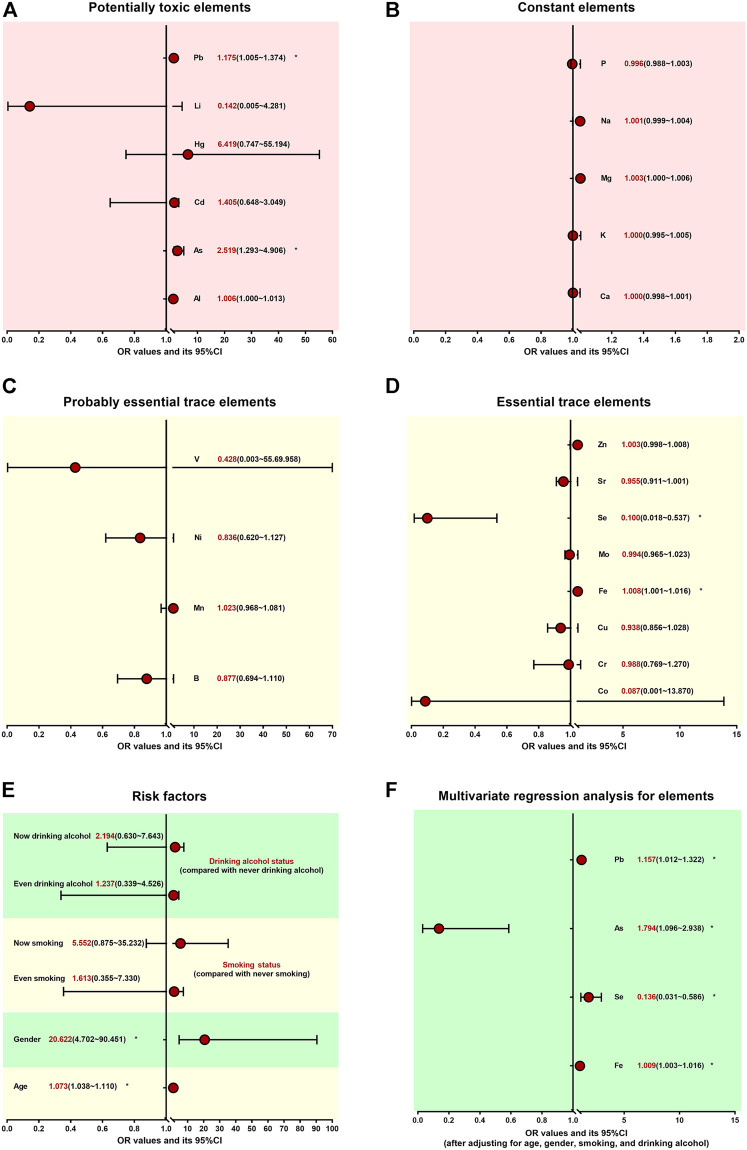
Association and risk between the element’s disorder and arsenism *p < 0.05. The univariate logistic regression analysis **(A–E)** shows the association and risk between the potentially toxic elements, constant elements, probably essential trace elements, essential trace elements, other factors, and arsenism, respectively. After adjusting for age, gender, smoking status, and drinking alcohol status, further multivariate logistic regression analysis results between the element’s disorder and arsenism are clearly shown **(F)**.

To study the association and risk between the element imbalances and arsenism, we divided all participants into control and arsenism. The univariate logistic regression analysis in Figures 2A–E and Supplementary Tables S1, S2 clearly shows that there was a significant correlation between age, gender, As, Pb, Fe, and Se, and arsenism (*p <* 0.05). The increasing age, As, Pb, and Fe content, and female gender can increase the risk of arsenism (*OR =* 1.073, 2.519, 1.176, 1.008, and 20.622). However, high Se is a protective factor and will reduce the risk of arsenism (*OR =* 0.100), which is presented in Figure 2E.

After adjusting for age, gender, smoking status, and drinking alcohol status, further multivariate logistic regression analysis shows that the increase in As, Fe, and Pb content is the independent risk factor for arsenism (*OR =* 1.794, 1.009, and 1.157). On the contrary, the high level of Se is the only independent protective factor for arsenism (*OR =* 0.136). These results are presented in Figure 2F and Supplementary Table S3.

**FIGURE 3:**
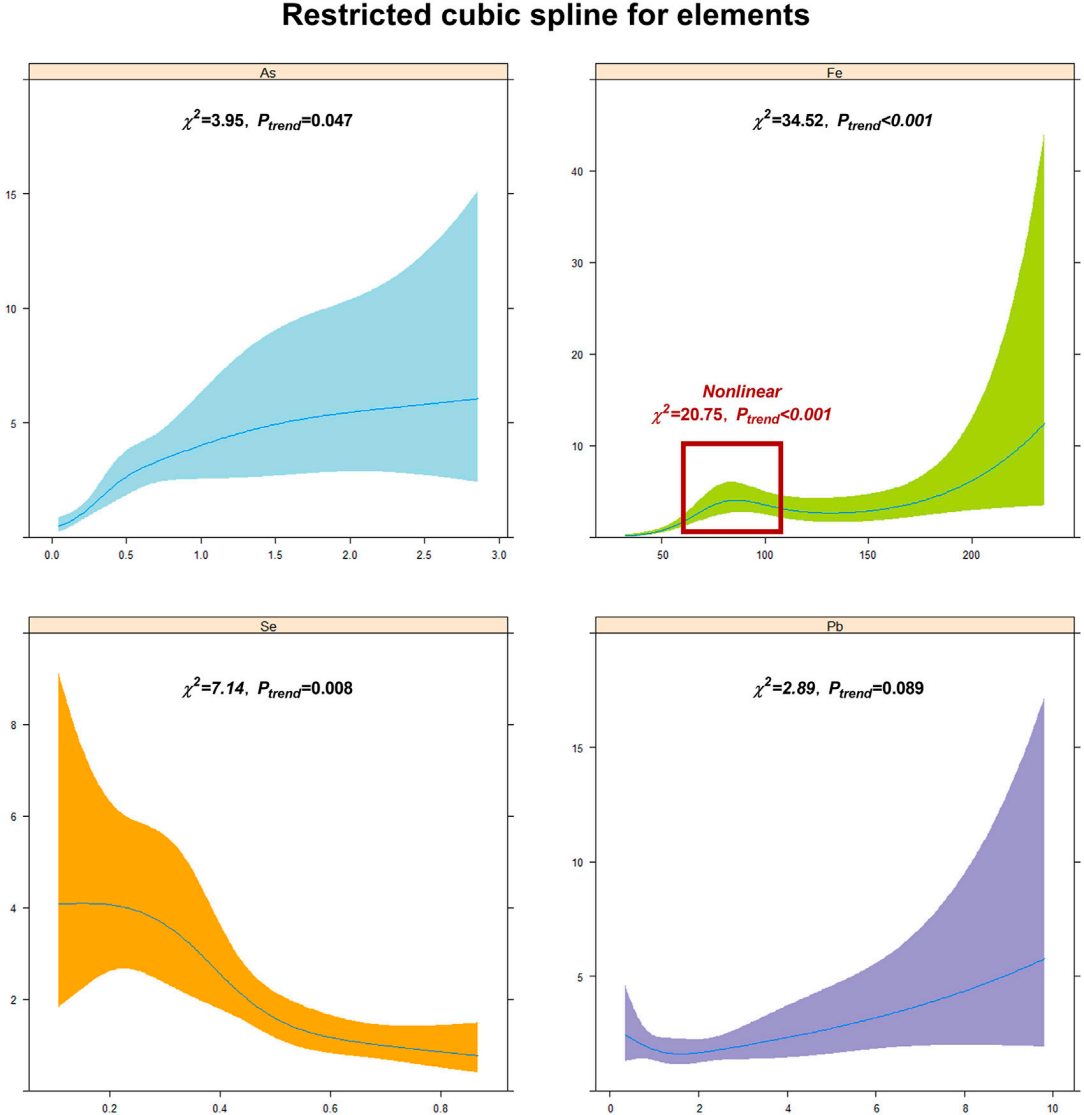
The linear and non-linear dose–response relationships between the content of As, Fe, Pb, Se, and arsenism. Restricted cubic splines are used to show the linear and non-linear dose–response relationships between the content of As, Fe, Pb, and Se and arsenism. The red box clearly shows that there is a significant non-linear dose–effect relationship between Fe and arsenism.

To further study the association and risk of the aforementioned element imbalances with arsenism, we analyzed the linear and non-linear dose–response relationships between the content of As, Fe, Pb, and Se in the hair and arsenism. The results of the linear and non-linear dose–response relationship analysis are shown in Figure 3. As illustrated in the figure, we can see that the content of As and Fe in the hair show a significant linear positive correlation with arsenism (*χ* ^*2*^_*linear*_ *=* 3.95, 34.52; *P*_*trend*_ *=* 0.047, <0.001) and has a significant linear negative correlation between the Se and arsenism (*χ* ^*2*^_*linear*_ *=* 7.14, *P*_*trend*_ *=* 0.008). In particular, the Fe content in the hair exhibits a non-linear correlation with arsenism in the range of approximately 75–125 mg/kg (*χ* ^*2*^_*non-linear*_ *=* 20.75, *P*_*trend*_ *<*0.001). No significant difference in the linear dose–response relationship was seen between the content of Pb with arsenism (*χ* ^*2*^_*linear*_ *=* 2.89, *P*_*trend*_ *=* 0.089).

**FIGURE 4:**
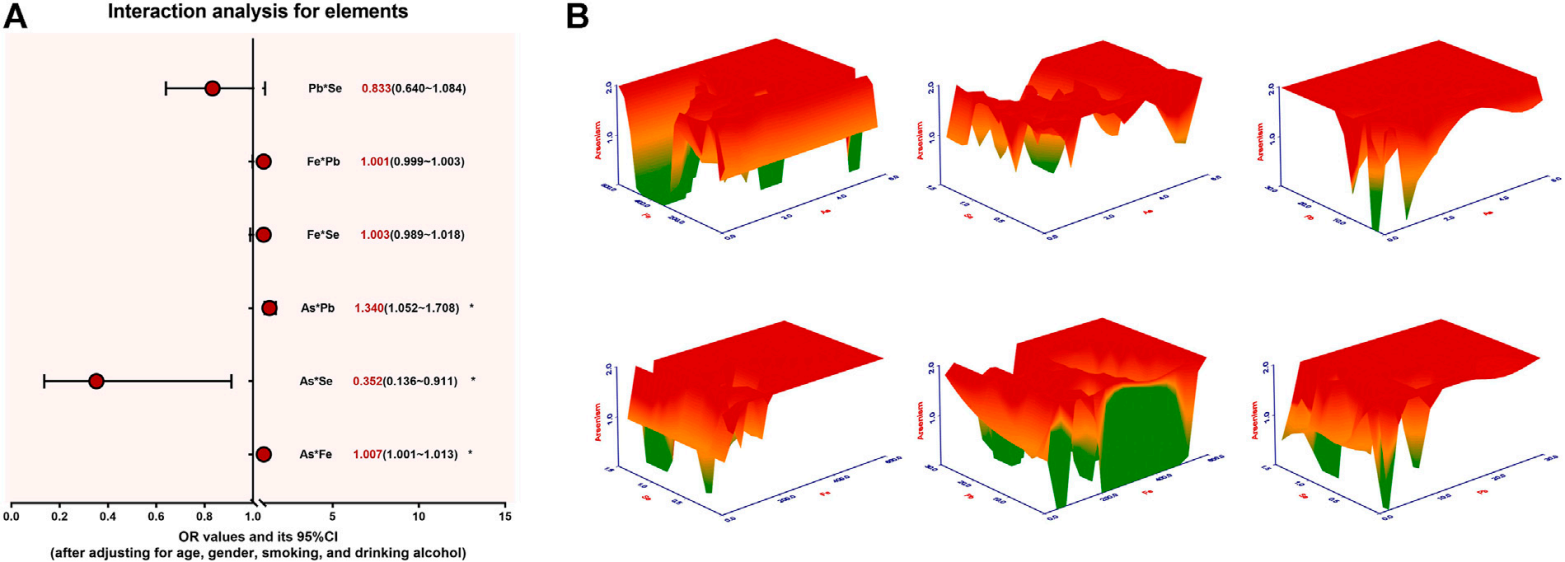
The interaction between the content of As, Fe, Pb, and Se for arsenism, *p < 0.05. **(A)** The interaction analysis results of As, Fe, Pb, and Se and arsenism. **(B)** The dose–response relationship between the effects of As, Fe, Pb, and Se alone and their combination on the pathogenicity of arsenism. As the color changes from green to yellow and then to red, the pathogenicity of arsenism gradually increases.

To further clarify the relationship between arsenic exposure-related element imbalances and arsenism, we conducted an interaction analysis. After adjusting for age, gender, smoking status, and drinking alcohol status, the results of the interaction analysis are shown in Figure 4 and Supplementary Table S4. For arsenism, it can be seen from the figure that there is a significant interaction between As and Pb (*OR =* 1.340), As and Fe (*OR =* 1.007), and As and Se (*OR =* 0.352). The combined exposure of Fe–As and Pb–As can increase the risk of arsenism, but the combined exposure of Se–As can reduce the risk of arsenism (*p <* 0.05). There are no significant differences in the interactions between Pb and Se, Fe and Pb, and Fe and Se.

### Potential Application Value of RRT on the Element Imbalances in the Population With Arsenism

**FIGURE 5:**
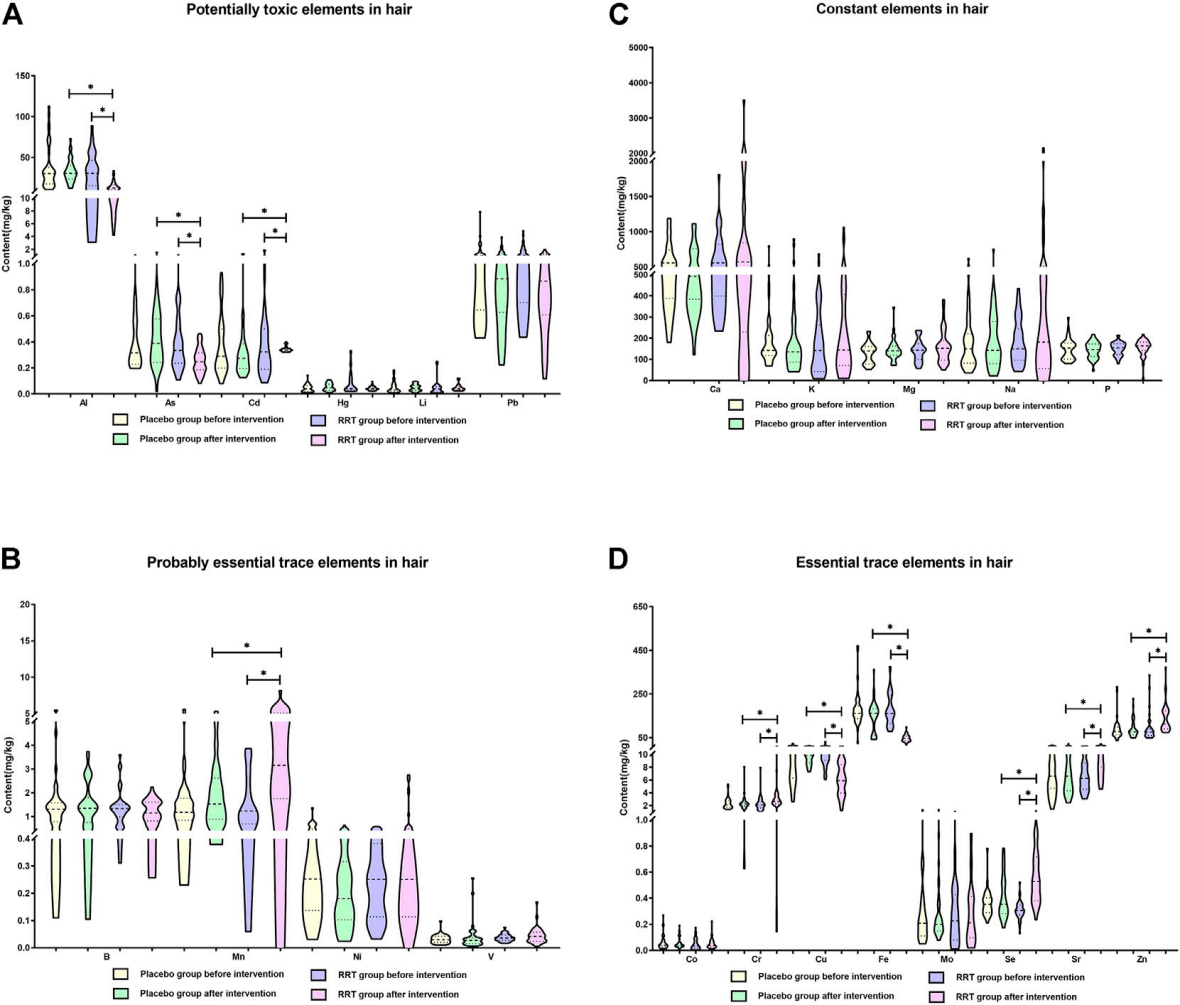
Potential application value of RRT on the element imbalances in the population with arsenism. In this study, the median and interquartile range were expressed in the results, *p < 0.05. **(A)** The changes in the content of potentially toxic elements. **(B)** The changes in the content of constant elements. **(C)** The changes in the content of probably essential trace elements. **(D)** The changes in the content of essential trace elements.

To assess the effect of *RRT* on the element imbalances in the population with arsenism, a randomized, controlled, double-blind intervention study of *RRT* lasting 3 months was designed. Figures 5A–D clearly show that *RRT* can improve the element imbalances in the population with arsenism (*p <* 0.05). Although the content of elements tends to change before and after the placebo intervention, the difference is not statistically significant (*p >* 0.05). Compared with the *RRT* group before intervention and the placebo group after intervention, the content of potentially toxic elements (Al, As, and Cd) and essential trace elements (Cu and Fe) in the *RRT* group after intervention gradually decreased (*p <* 0.05). Moreover, the content of probably essential trace element (Mn) and essential trace elements (Cr, Se, and Sr) in the *RRT* group after the intervention is higher than that in the *RRT* group before intervention and the placebo group after intervention (*p <* 0.05). However, there is no significant difference among other elements in the different groups (*p >* 0.05).

## Discussion

Trace elements are essential substances for the normal life of the human body, which play a very important role in the composition and physiological process of the body (Xiu, 1996). However, excessive concentrations of trace elements can also be toxic to the body (Zeng et al., 2021; Zheng et al., 2021), such as arsenic, which is an essential trace element, and the residents living under certain geographical environmental conditions can also be affected by chronic arsenism by ingesting excessive amounts of inorganic arsenic through drinking water, air, or food for a long time (Wei et al., 2018a). Hyperkeratosis, hyperpigmentation, and carcinogenesis of the skin are the main hallmarks of chronic arsenism (Wei et al., 2018a). Previous studies (Wei et al., 2018a; Wei et al., 2018b; Li et al., 2019a; Hu et al., 2020; Outa et al., 2020; Hu et al., 2021; Çiner et al., 2021) demonstrate that arsenism is a disease with disorder of various elements. The increase or decrease of elements in the body will induce different health problems, and the elements will also interact with each other. Therefore, from the perspectives of multi-element interaction and linear and non-linear dose–response relationships to explore the effects of element imbalances in the pathogenesis of arsenism, it is expected to deepen the understanding of the pathogenesis of arsenism from a new perspective.

The hair is recommended by the World Health Organization, Environmental Protection Agency, and International Atomic Energy Agency as an important biological material for global environmental monitoring (Druyan et al., 1998; Morton et al., 2002). Based on exposure and disease grouping, our results demonstrated that arsenic can increase the content of Al, As, Hg, and Fe in the hair while reducing the content of Co, Cu, Se, Sr, and Zn. It is suggested that the changes of the aforementioned elements should be dynamically observed in the health monitoring of the exposed population to take targeted preventive and control measures. Several studies (Samanta et al., 2004; Spallholz et al., 2005; Agusa et al., 2006; Barati et al., 2010; Wei et al., 2018b; Hu et al., 2021) observed an increase in Mn, Ni, V, and Pb content in the hair collected from people with arsenism. Our research did not indicate a similar conclusion, which may be related to the difference in the survey area, because there is a significant correlation between the uneven distribution of elements in the environment and the trace element imbalances in individuals exposed to arsenic (Barati et al., 2010). These findings provide limited evidence for the link between the element imbalances and arsenism.

Hair arsenic is useful as an exposure biomarker, reflecting the arsenic intake of the chronic arsenism population (Hindmarsh, 2002). Our results revealed that the content of As in the arsenic exposure group and arsenism group was higher than that in the non-arsenic exposure group and control group. These results are consistent with previous studies on different types of arsenism (such as drinking water type and coal burning type of arsenism) in India (Samanta et al., 2004), Iran (Barati et al., 2010), Vietnam (Agusa et al., 2006), and China (Wei et al., 2018b; Hu et al., 2021). They provide further support for the association between chronic arsenic exposure and arsenism. Furthermore, the logistic regression analysis results demonstrate that the high levels of As content in the hair were a risk factor for arsenism. This further provides more evidence to support the theory that chronic arsenic exposure is the root cause of arsenism.

Fe is a very important essential trace element and participates in many physiological processes of the body (Dev and Babitt, 2017). Fe supplementation is an effective treatment for many patients with anemia, but excessive iron is also toxic (Dev and Babitt, 2017). In this study, our results indicated that arsenic could upregulate the content of Fe in the hair, which is consistent with our previous finding (Hu et al., 2020). It is suggested that arsenic can promote Fe accumulation in the body by regulating Fe metabolism. Cu, Se, and Zn are components of many antioxidant enzymes, such as superoxide dismutase, glutathione peroxidase, and reduced glutathione. Several studies (Pimparkar and Bhave, 2010; Hunt et al., 2014) have shown that oxidative stress is one of the main mechanisms of arsenic pathogenicity. Also, oxidative stress consumes a lot of antioxidant enzymes, thereby reducing the content of Cu, Se, and Zn in the body. Our research has reached a similar conclusion; that is, the Cu, Se, and Zn levels in the hair of people exposed to arsenic are reduced. A previous study (Singh and Kanwar, 1981) has also found that low levels of Cu, Zn, and Cu can promote Fe metabolism, thereby increasing the accumulation of Fe in the main organs of rats. These results provide a possible hypothesis that arsenic may reduce the levels of Cu, Se, and Zn through oxidative stress, thereby promoting the accumulation of Fe in the body. In view of the logistic regression analysis results, it is shown that high levels of Fe and reduced Se will increase the risk of arsenism; therefore, we recommend adding Cu, Se, and Zn to the diet to better protect the health of people exposed to arsenic. Although we have observed an association between high levels of Fe and arsenism, it is surprising that this association is not a simple linear dose–response relationship. On the contrary, the Fe content in the hair exhibits a non-linear correlation with arsenism in the range of approximately 75–125 mg/kg. Whether this is related to the low-dose hormesis effect of iron requires further study. Overall, current findings suggest that controlling the Fe content in the body within an appropriate range can reduce the health hazards caused by arsenic exposure.

Pb is a common accumulated poison in the body. A previous study (Pham et al., 2017) has shown that the content of Pb in the biological tissues (hair, nails, and skin scales) of people with arsenism is higher than that of the control group, but this study did not get similar results. The linear dose–response relationship between Pb and arsenism is not obvious. Whether this is related to the different survey locations is worthy of further study. Nevertheless, both univariate and multivariate logistic regression models indicate that the increased level of lead in the hair was an independent risk factor for arsenism. This result suggests that we cannot ignore the impact of Pb in the arsenism population. An existing study (Mazumdar et al., 2017) has shown that Ca can competitively prevent the absorption of Pb. Therefore, we can also reduce the health hazards of Pb on arsenism by supplementing Ca and reducing lead intake.

Subsequently, we analyzed the interaction between the elements to further clarify the relationship between arsenic exposure-related element disorders and arsenism. Se is a strong antioxidant that exerts antioxidant effects by enhancing GSH-Px activity (Spallholz et al., 2005). Our results indicate that the combined exposure of high content of Se and As could significantly reduce the risk of diseases caused by arsenic exposure alone. Combined with oxidative stress as one of the key mechanisms of arsenic pathogenesis, it is suggested that Se mainly exerts its detoxification effect on arsenic poisoning through the antioxidant effect. The Fe element is also one of the essential elements present in the human body. A previous study (Kordas et al., 2017) has found that Fe can promote the excretion of As. Our research shows that the Fe content in the hair has a non-linear relationship with arsenic poisoning; that is, the low content of Fe is the risk factor of arsenism. Second, the risk of arsenism gradually decreased with the increase in Fe content (ranging from approximately 75–125 mg/kg). Finally, the risk of arsenism gradually increased with the content of Fe above 125 mg/kg. These results are reasonable. At low Fe levels, the excretion of Fe on As cannot offset the toxic effects of arsenic; with the increase in iron content, the level of arsenic gradually decreases, thus exerting its protective effect on patients with arsenism. When Fe content is further increased, the iron overload will induce ferroptosis in body cells, thereby aggravating arsenism. Several studies have shown that Fe overload is the central link in ferroptosis (Dixon and Stockwell, 2014) and also participated in arsenic-induced male reproductive toxicity (Meng et al., 2020), pancreatic dysfunction (Xia et al., 2020), and neurodegenerative diseases (Tang et al., 2018). Animal experiments have found that Pb can antagonize the toxicity of arsenic (Agrawal et al., 2015; Aktar et al., 2017). Our research did not support this view because the combined exposure of Pb–As can increase the risk of arsenism, and the reason remains to be further explored. These results suggest that the combined exposure of Fe–As and Pb–As can increase the risk of arsenism, but the combined exposure of Se–As can reduce the risk of arsenism. This finding represents another important issue, which is to find some natural medicinal plants and fruits rich in trace element such as Se to effectively detoxify arsenism.

Diet is a good way to treat metabolic diseases and aging (Le Couteur et al., 2021). Some studies (Chen et al., 2007; Mahata et al., 2008; Zablotska et al., 2008; Xu et al., 2016; Xia et al., 2020; Xu et al., 2021b) have shown that vitamins, trace elements, and natural medicinal plants can be used to prevent and treat endemic arsenism. *RRT*, a traditional Chinese health food that is unique to the mountainous area of southwest China (An et al., 2011; Shi et al., 2020), contains a variety of biologically active metabolites (such as pentacyclic triterpenoids and flavonoids) and rich nutrients (including trace elements, vitamins, polysaccharides, dietary fiber, unsaturated fatty acids, and superoxide dismutase) (Wu et al., 2020). Our previous animal study found that *RRT* can attenuate liver damage in arsenic-poisoned rats by regulating element balance and oxidative stress (Xu et al., 2021b). In this study, our results show that *RRT* could increase the essential trace elements (Cr, Se, and Sr) and reduce the potentially toxic elements (Al, As, and Cd) and harmful element (Fe). These findings suggest that the detoxification effect of *RRT* on arsenism is mainly achieved by alleviating the element imbalances. In addition, Se is a major component of many antioxidant enzymes, such as superoxide dismutase, glutathione peroxidase, and reduced glutathione. This also provides some evidence that *RRT* can also exert its anti-oxidative effect on arsenism by supplementing the Se that is needed to produce antioxidant enzymes, thereby detoxifying arsenism.

The strengths of our study include the perspective of multi-element interaction to explore the role of element disorders in the pathogenesis of arsenic. At the same time, a randomized, controlled, double-blind intervention study of *RRT* lasting 3 months was designed to explore the potential application value of *RRT*. However, the health hazards in endemic arsenism have the characteristics of accumulation and irreversibility, and there is no direct population evidence that *RRT* can improve the skin, lung, and liver lesions caused by arsenism. Therefore, it is necessary to conduct a more in-depth study on *RRT* from the arsenic exposure stage to prevent the occurrence of arsenism. In addition, the regulation mechanism of RRT involves antioxidant, immune regulation, anti-inflammatory, trace element regulation, sleep improvement, and so on. This study aimed to explore how *RRT* can detoxify arsenism by alleviating the element imbalances and did not evaluate the role of other mechanisms of *RRT* to prevent arsenism. Furthermore, given the limitations of epidemiological studies, the toxicant interactions observed in this study remain to be verified by experimental studies.

## Conclusion

**FIGURE 6:**
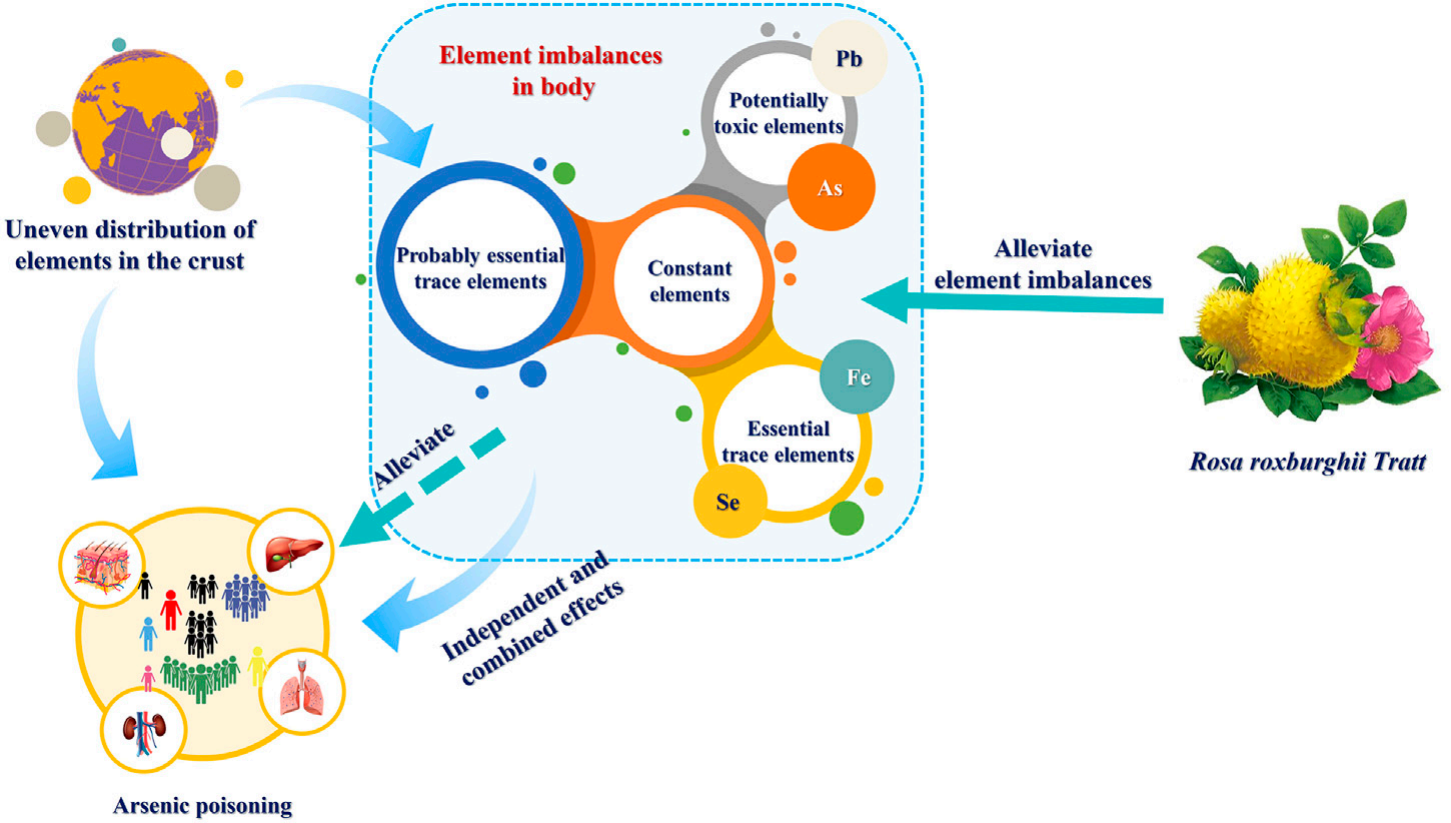
Association of element imbalances with arsenism and the potential application value of *RRT*.

Overall, our study provides some limited evidence that the element imbalances (the high level of As, Fe, and Pb and the low level of Se) are the risk factors for the occurrences of arsenism. The second major finding was that *RRT* can regulate the element imbalances, which is expected to improve the arsenism (Figure 6). This study provides a scientific basis for further understanding a possible traditional Chinese health food, *RRT*, as a more effective detoxication of arsenism.

## Data Availability

The original contributions presented in the study are included in the article/Supplementary Material, further inquiries can be directed to the corresponding author.

## Abbreviations

Al, aluminum; As, arsenic; B, boron; Ca, calcium; Cd, cadmium; Co, cobalt; Cr, chromium; Cu, copper; Fe, iron; Hg, mercury; K, kalium; Li, lithium; Mg, magnesium; Mn, manganese; Mo, molybdenum; Na, natrium; Ni, nickel; *RRT*, *Rosa roxburghii* Tratt juice; Se, selenium; Sr, strontium; V, vanadium; and Zn, zinc.
