# Supplementary material for: Assessing the Association of Element Imbalances With Arsenism and the Potential Application Value of Rosa roxburghii Tratt Juice
Source: Front Pharmacol. 2022 Apr 25;13:819472. doi: 10.3389/fphar.2022.819472 (PMC9082068; doi:10.3389/fphar.2022.819472)
Supplement: Supplementary file 1 [file DataSheet2.PDF]

Table S1 Variable assignment in the study

Variables	Variable assignment
**Arsenism**	
No	0
Yes	1
**Age**	Original value
**Gender**	
Male	1
Female	2
**Smoking status**	
Never smoking	0
Even smoking	1
Now smoking	2
**Dring alcohol status**	
Never drinking	0
Even drinking	1
Now drinking	2
**Potentially toxic elements**	
Al	Original value
As	Original value
Cd	Original value
Hg	Original value
Li	Original value
Pb	Original value
**Constant elements**	
Ca	Original value
K	Original value
Mg	Original value
Na	Original value
P	Original value
**Probably essential trace elements**	
B	Original value
Mn	Original value
Ni	Original value
V	Original value
**Essential trace elements**	
Co	Original value
Cr	Original value
Cu	Original value
Fe	Original value
Mo	Original value
Se	Original value
Sr	Original value
Zn	Original value

Table S2 Univariate logistic regression analysis of the elements and arsenism

Variables	β	Wald	*P values*	OR values	95%CI for OR
**Age**	0.071	16.813	<0.001	1.073	1.038~1.110
**Gender**	3.026	16.096	<0.001	20.622	4.702~90.451
**Smoking status**		4.061	0.131		
Even smoking	1.714	3.306	0.069	5.552	0.875~35.232
Now smoking	0.478	0.383	0.536	1.613	0.355~7.330
**Drinking alcohol status**		2.101	0.350		
Even drinking	0.786	1.523	0.217	2.194	0.630~7.643
Now drinking	0.213	0.104	0.747	1.237	0.339~4.516
**Potentially toxic elements**					
Al	0.006	3.457	0.063	1.006	1.000~1.013
As	0.924	7.379	0.007	2.519	1.293~4.906
Cd	0.340	0.740	0.390	1.405	0.648~3.049
Hg	1.859	2.869	0.090	6.419	0.747~55.194
Li	-1.948	1.260	0.262	0.142	0.005~4.281
Pb	0.161	4.084	0.043	1.175	1.005~1.374
**Constant elements**					
Ca	0.000	0.319	0.572	1.000	0.998~1.001
K	0.000	0.000	0.985	1.000	0.995~1.005
Mg	0.003	3.433	0.064	1.003	1.000~1.006
Na	0.001	1.848	0.174	1.001	0.999~1.004
P	-0.004	1.324	0.250	0.996	0.988~1.003
**Probably essential trace elements**					
B	-0.131	1.186	0.276	0.877	0.694~1.110
Mn	0.023	0.664	0.415	1.023	0.968~1.081
Ni	-0.179	1.384	0.239	0.836	0.620~1.127
V	-0.849	0.107	0.744	0.428	0.003~69.958

**Variables**	**β**	**Wald**	***P values***	**OR values**	**95%CI for OR**
**Essential trace elements**					~
Co	-2.447	0.893	0.345	0.087	0.001~13.870
Cr	-0.012	0.009	0.924	0.988	0.769~1.270
Cu	-0.064	1.873	0.171	0.938	0.856~1.028
Fe	0.008	4.951	0.026	1.008	1.001~1.016
Mo	-0.006	0.180	0.671	0.994	0.965~1.023
Se	-2.307	7.197	0.007	0.100	0.018~0.537
Sr	-0.046	3.689	0.055	0.955	0.911~1.001
Zn	0.003	1.297	0.255	1.003	0.998~1.008

Model 1 was used to determine the relationship between 23 elements, effect factors (including age, gender, smoking, drinking) and arsenism.

Table S3 Multivariable logistic regression analysis of the elements and arsenism

Variables	β	Wald	*P values*	OR values	95%CI for OR
**As**	0.585	5.399	0.020	1.794	1.096~2.938
**Fe**	0.009	8.851	0.003	1.009	1.003~1.016
**Pb**	0.146	4.560	0.033	1.157	1.012~1.322
**Se**	-1.998	7.159	0.007	0.136	0.031~0.586

The statistically significant elements in model 1 analysis along with age, gender, smoking status and drinking alcohol status are placed into the model together. After adjusting for age, gender, smoking status and drinking alcohol status, model 2 was applied to analyze the independent factors associated with arsenism and estimates its risks.

Table S4 Interaction analysis of the elements and arsenism

Variables	β	Wald	*P values*	OR values	95%CI for OR
As*Fe	0.007	5.193	0.023	1.007	1.001~1.013
As*Pb	0.293	5.623	0.018	1.340	1.052~1.708
As*Se	-1.043	4.636	0.031	0.352	0.136~0.911
Fe*Pb	0.001	0.843	0.358	1.001	0.999~1.003
Fe*Se	0.003	0.212	0.645	1.003	0.989~1.018
Pb*Se	-0.183	1.844	0.175	0.833	0.640~1.084

The statistically significant elements in model 2 analysis are placed into the model together, after adjusting for age, gender, smoking and drinking, model 3 was used to analyze the interaction between the elements and arsenism, and estimates its risks.
