# Supplementary material for: Assessing the Association of Element Imbalances With Arsenism and the Potential Application Value of Rosa roxburghii Tratt Juice
Source: Front Pharmacol. 2022 Apr 25;13:819472. doi: 10.3389/fphar.2022.819472 (PMC9082068; doi:10.3389/fphar.2022.819472)
Supplement: Supplementary file 2 [file DataSheet1.PDF]

No.	Group	Al	As	Cd	Hg	Li	Pb	Ca	K	Mg
3	2	70.7492	5.1068	0.0020	0.1165	0.0174	0.9383	366.6465	209.0589	156.5452
6	2	128.2803	3.9016	0.0262	0.3615	0.0481	3.2944	684.0528	388.2854	241.0873
7	2	168.5863	3.8747	0.1447	0.0717	0.0212	8.5351	456.9369	393.0742	178.1493
8	2	89.5163	0.9693	0.0020	0.0255	0.0236	1.1817	956.6458	130.9495	519.1929
10	2	129.4199	3.6199	0.1181	0.0135	0.0092	2.7270	535.1714	187.4904	181.6752
13	2	56.7818	3.5581	0.8711	0.1958	0.0059	1.4978	1352.7209	20.1627	314.9615
15	2	50.3036	0.0020	0.0020	0.2032	0.0109	1.3005	1166.2727	139.3745	313.0579
16	2	224.3970	0.0023	2.1071	0.7135	0.0564	4.3547	1224.9474	124.0806	619.6146
17	2	151.2601	2.9927	0.0020	0.0955	0.0335	2.6859	1864.9676	207.0777	791.1270
20	2	23.5345	2.5783	2.3642	0.3265	0.4581	0.6787	641.4767	75.2657	92.2106
21	2	71.1541	2.3885	0.0767	0.2355	0.0062	6.5329	501.7567	408.9364	158.7727
22	2	68.4538	0.0105	0.0020	0.1236	0.0017	0.7658	378.0617	126.2942	166.1145
23	2	106.5505	2.3821	0.0020	0.0932	0.0065	1.5225	588.3933	161.3235	218.6249
24	2	223.6665	1.8805	0.0020	0.0123	0.0265	1.2938	543.5707	188.2507	199.0196
26	2	201.1118	0.6316	0.0020	0.0255	0.0166	1.0156	537.0151	132.5532	211.7554
27	2	630.1739	1.7749	0.0020	0.0136	0.0693	0.8351	1705.8353	180.6246	314.8285
28	2	133.6751	1.1701	0.1893	0.3127	0.0401	6.4163	700.4523	131.9354	229.3733
29	2	125.6345	1.5165	0.5044	0.6095	0.0105	20.1443	492.1648	176.8256	166.3901
31	2	561.2968	1.4321	0.0020	0.7122	0.0043	0.8363	1530.4504	159.4201	281.9638
32	2	35.1392	1.4125	0.0228	0.2002	0.0221	1.9088	607.4073	274.4906	215.7681
34	2	411.3506	2.0824	0.0221	0.1471	0.0034	2.8301	529.2244	172.7919	195.6748
37	2	165.5717	1.0772	0.0280	0.6355	0.0038	4.2089	508.4913	183.0340	138.2645
39	2	35.5379	0.4927	0.0020	0.2020	0.1054	1.2702	441.0998	285.8634	178.8397
40	2	80.1062	0.0175	0.0020	0.0303	0.0375	1.4359	1522.3649	178.4321	725.5145
41	2	82.9477	0.5456	0.0020	0.1124	0.0051	1.9425	1635.7976	141.1626	720.2405
42	2	50.5550	0.4878	0.0044	0.1524	0.0410	2.3992	660.6863	214.7874	197.1602
44	2	88.5684	0.4795	0.0130	0.0211	0.1241	3.2147	644.5425	309.7143	245.5126
46	2	407.7898	0.7842	0.0020	0.1476	0.0113	1.4067	518.6055	118.6929	209.7832
47	2	37.6818	0.4617	0.0020	0.2365	0.0068	1.8350	372.4300	206.4749	154.0872
49	2	263.8414	0.4531	0.0020	0.6315	0.0042	4.1051	1468.7254	142.5074	534.3433
50	2	174.8575	2.5974	0.0020	0.0321	0.0059	1.7359	873.2790	181.1607	550.0897
51	2	336.8693	0.0175	0.0020	0.1632	0.0134	2.1760	449.0336	240.4917	166.4997
53	2	146.1011	0.4471	0.0151	0.4365	0.0237	2.1287	1027.0945	134.0578	475.3118
54	2	20.6668	0.4461	0.0236	0.3544	0.0221	1.0909	1270.8311	89.0590	158.1785
56	2	109.2264	0.7455	0.0033	0.2456	0.0058	3.5785	863.8069	202.9970	232.6187
57	2	140.2361	0.4438	0.0020	0.0355	0.1501	2.5695	527.9119	331.0701	146.5575
59	2	318.3259	0.4546	0.0020	0.1525	0.1327	1.4409	458.6909	124.2435	152.6806
62	2	178.6444	0.4327	0.0020	0.2325	0.0525	1.0394	401.5902	139.8179	176.5607

No.	Group	Al	As	Cd	Hg	Li	Pb	Ca	K	Mg
64	2	30.7529	0.4260	0.0020	0.0196	0.0030	0.4636	409.2079	135.3082	192.4774
66	2	141.5604	0.4240	0.0768	0.0896	0.0744	6.6540	962.5186	158.7189	348.3361
67	2	226.0719	0.4184	0.0020	0.0064	0.0042	1.2789	596.7029	182.8659	224.2517
68	2	56.0963	0.4119	0.0574	0.0193	0.0008	3.6692	570.7101	180.0267	264.4588
69	2	111.1604	0.9530	0.0226	0.0302	0.0070	3.9286	927.0864	296.7436	256.2322
71	2	47.4308	0.4096	0.0020	0.0232	0.0193	1.7932	460.3657	270.4567	208.8923
72	2	208.5969	0.8871	0.0360	0.0668	0.0048	4.8105	1182.4617	479.1764	419.5225
73	2	166.4664	0.8770	0.3273	0.0175	0.0038	8.7761	442.9259	174.9455	132.1907
74	2	93.5396	0.8382	0.0020	0.1253	0.0115	2.7370	450.7917	183.1055	198.3895
75	2	51.6016	0.8116	0.0020	0.0920	0.0210	1.6500	647.6458	227.5477	352.9066
76	2	174.7203	0.8102	0.0020	0.0522	0.0042	1.2293	506.9157	87.8955	174.5358
78	2	209.6828	0.8044	0.0020	0.0235	0.0048	2.1569	700.3191	170.3941	361.7744
79	2	164.3720	1.3934	0.0020	0.1399	0.0167	1.7565	850.9876	181.0427	259.1448
81	2	95.8127	1.6097	0.0020	0.2365	0.0064	2.6683	441.1199	168.6466	134.5013
82	2	89.1652	0.7845	0.0020	0.1654	0.0036	3.0025	1012.8441	195.5120	485.8973
83	2	116.8042	0.7829	0.0020	0.0782	0.0079	2.5044	1106.8892	183.1092	588.6049
84	2	111.6910	0.7812	0.0020	0.1633	0.0237	0.7935	381.9075	93.8435	130.1782
85	2	53.5262	0.7626	0.0020	0.2032	0.0057	3.1054	562.3487	448.4217	257.4028
86	2	305.8541	0.6207	0.0020	0.3042	0.0133	5.8562	1330.8262	172.4775	711.3912
89	2	135.5012	0.7545	0.0113	0.2053	0.1623	3.6363	1036.3155	677.3675	390.3903
90	2	206.9802	0.7210	0.0020	0.0688	0.0037	3.1664	1456.6166	194.2593	671.1574
92	2	93.0326	0.7008	0.0020	0.1254	0.0094	2.5083	408.7893	240.7519	189.2733
93	2	148.5167	0.5527	0.0020	0.0230	0.0068	5.0822	459.3531	256.8071	166.7139
94	2	248.9811	0.5248	0.0020	0.0192	0.0113	1.8493	995.8808	96.9522	374.6274
95	2	101.8566	0.6980	0.0020	0.1235	0.0109	5.0482	977.2146	132.7414	431.8851
96	2	107.9363	2.0428	2.8514	0.0963	0.0143	8.2862	1909.6699	2284.0941	670.7609
97	2	21.6717	0.6931	0.0020	0.0363	0.0201	0.7599	450.7601	400.2049	184.5997
98	2	118.8342	0.6898	0.0443	0.1263	0.0173	5.4954	675.3560	218.9332	218.7355
100	2	102.4263	0.6870	0.0851	0.0445	0.0071	4.6157	775.1748	147.1345	167.4811
102	2	37.0687	0.7951	0.1999	0.0624	0.0051	0.9897	249.8877	103.9026	69.8913
103	2	71.5113	0.6418	0.1407	0.0585	0.0178	2.4930	1657.3934	121.5659	694.1639
105	2	33.4247	0.6013	0.0187	0.0140	0.0126	1.7598	505.0361	161.0197	160.8183
107	2	59.3240	0.6265	0.0020	0.0296	0.0087	4.8391	766.8527	131.7573	272.9769
109	2	21.9591	0.6216	0.0090	0.0334	0.0264	1.3754	534.8097	182.2345	193.8999
111	2	149.0497	0.4020	0.0020	0.0732	0.0053	1.1544	372.3750	95.3020	169.5119
119	2	201.3498	0.6190	0.0020	0.0635	0.0041	1.4901	446.7115	138.1880	209.7797
124	2	89.8952	0.7077	0.0020	0.1037	0.0013	2.0024	498.0934	483.6591	208.3699
126	2	93.7930	1.2250	0.0151	0.0896	0.0357	2.3077	1040.8264	355.4709	391.3324

No.	Group	Al	As	Cd	Hg	Li	Pb	Ca	K	Mg
127	2	113.6623	0.6123	0.0020	0.0358	0.0785	0.9442	332.8798	95.6113	152.5521
128	2	26.9256	0.6101	0.1946	0.0762	0.0167	0.2285	190.0449	49.8796	43.8680
129	2	87.4297	0.6073	0.1590	0.1225	0.0524	30.9620	869.9886	199.6727	208.4239
130	2	179.3578	0.5810	0.0020	0.2347	0.0713	1.0215	604.7386	339.6268	263.3062
131	2	26.1523	0.5734	0.0020	0.1574	0.0036	0.9162	459.1775	253.0421	183.9502
132	2	101.2988	0.5720	0.0020	0.6357	0.0124	0.9824	1566.1250	136.0920	619.6430
136	2	412.2093	0.5349	0.0020	0.2106	0.0053	1.8612	504.8295	72.6708	266.0452
138	2	79.4919	0.5233	0.0020	0.1082	0.0048	0.4152	321.0596	118.8685	156.7239
139	2	60.8373	0.0283	0.6353	0.0365	0.0075	3.7158	465.1842	203.3242	220.1544
140	2	56.9410	1.2426	0.0442	0.0890	0.0056	9.5129	1078.9794	212.5098	433.4437
145	2	67.3912	0.5212	0.0479	0.0695	0.0013	3.0513	394.7701	334.8247	162.4469
148	2	25.6173	0.5202	0.0285	0.3578	0.0159	2.1569	260.4845	100.4195	60.5172
150	2	52.4621	0.9087	0.0342	0.4664	0.0011	1.7612	439.1321	124.4292	160.1739
152	2	79.3372	0.5144	0.0020	0.7117	0.0332	1.2572	426.0901	111.7636	199.0676
155	2	43.2779	1.3981	0.0020	0.0639	0.0005	0.9496	450.9831	164.9914	212.5603
158	2	71.4358	1.3803	0.0144	0.0492	0.0723	4.4349	1082.7284	145.1886	502.2231
159	2	64.9277	1.3009	0.0020	0.0803	0.0040	1.8992	848.7642	365.9186	253.2321
163	2	86.0312	1.4560	0.0201	0.1886	0.0069	2.0137	1061.8338	239.5016	512.5224
165	2	45.7143	0.8518	0.0020	0.2549	0.1915	4.8757	387.6934	214.8868	194.6961
167	2	151.8745	1.2479	0.0121	0.3144	0.5912	3.8699	1043.2410	3086.3397	340.5723
169	2	40.6215	1.2472	0.0710	0.5346	0.0132	2.8667	636.2071	252.8770	94.4274
170	2	34.7944	1.2394	0.3129	0.9216	0.0052	0.4550	269.1397	86.7366	70.2356
173	2	85.2762	1.1819	0.0020	0.0653	0.0061	2.3938	965.5562	129.8433	436.3776
174	2	70.0962	0.6445	0.0020	0.0703	0.0607	0.6944	674.3225	105.9015	369.3082
175	2	563.8700	0.4479	0.0020	0.0836	0.0299	3.3923	478.2358	106.3124	238.0860
177	2	40.4561	0.5635	0.0567	0.4104	0.0145	2.5054	516.7110	255.1405	166.0514
178	2	51.6438	1.0246	0.0020	0.1566	0.1719	3.1387	312.5931	294.5411	123.5693
183	2	58.5187	1.0221	0.0020	0.2487	0.0162	2.8031	509.4386	306.7362	174.7656
187	2	66.9707	0.0386	0.0066	0.1249	0.7190	2.4476	1851.2357	146.4055	570.1990
189	2	32.7622	0.6662	0.0020	0.0855	0.0202	1.2657	484.0362	190.5260	216.4999
191	2	46.4934	2.7282	0.0441	0.1555	0.0347	5.1061	465.0765	1768.5737	222.1950
192	2	98.1768	0.9997	0.0380	0.0935	0.0022	3.9994	629.6662	466.6527	240.3273
194	2	54.1605	0.9584	0.0020	0.0815	0.0262	2.4200	446.9389	105.5667	172.5723
196	2	28.3305	0.9234	0.0276	0.0632	0.0364	1.6539	575.4055	125.4799	235.5120
199	2	20.6241	0.9192	0.1916	0.0433	0.0095	4.3028	436.2396	162.3536	204.2093
200	2	70.5708	0.9068	0.1046	0.1508	0.0933	2.3754	957.9115	188.9739	315.4854
201	2	257.8727	0.3995	0.4996	0.0125	0.1316	2.5406	404.4723	45.5462	70.5382
203	2	22.6936	0.7603	0.2337	0.1247	0.0151	1.5039	216.2190	209.9414	56.0735

No.	Group	Al	As	Cd	Hg	Li	Pb	Ca	K	Mg
204	2	28.3839	0.4665	0.2757	0.0316	0.0364	1.0450	670.6765	19.3869	296.6716
205	2	18.9565	0.5605	0.2096	0.0216	0.0939	0.5431	263.8193	56.2312	66.7333
206	2	32.1979	0.5490	0.2205	0.0913	0.0032	4.2575	439.9068	28.6106	138.2207
209	2	33.2434	0.3837	0.2286	0.0822	0.0472	1.3576	517.2455	93.0545	68.7080
211	2	86.0187	0.3750	0.0020	0.0735	0.0096	2.6994	533.0963	195.5769	221.8677
213	2	43.8793	0.3746	0.2049	0.0666	0.1791	0.8257	181.1210	34.1630	39.2121
214	2	35.0737	0.0388	0.2226	0.1016	0.0148	0.3221	374.0318	118.8789	126.8942
215	2	27.0575	0.7568	0.2164	0.1346	0.0250	0.7541	204.7863	101.2432	58.4227
217	2	67.9044	0.3543	0.4319	0.7566	0.0428	1.0239	874.4144	42.2814	237.9949
219	2	35.9136	0.3419	0.2446	0.1248	0.0040	1.0232	272.1536	25.3593	45.7659
220	2	11.1445	0.4020	0.0020	0.0963	0.0142	0.0200	404.0625	11.8505	9.2444
221	2	28.6402	0.3363	0.4916	0.1525	0.0232	3.0833	327.8415	193.6017	98.3923
224	2	22.2708	0.9193	0.2367	0.3556	0.0293	1.0382	193.3555	89.6164	46.9114
226	2	32.3096	0.7188	0.4494	0.0632	0.0412	1.1790	328.1741	110.4684	87.9403
227	2	19.4918	0.0454	0.1961	0.0732	0.0179	0.7536	709.3237	29.3493	212.2918
229	2	79.2821	0.3355	0.4498	0.0932	0.0261	0.9919	902.8152	38.3373	297.4534
231	2	21.7556	0.3335	0.2198	0.1091	0.0055	0.5723	232.5356	63.4235	59.9294
232	2	20.4029	0.5904	0.1864	0.0763	0.0051	0.7470	149.8521	45.5316	38.5089
233	2	30.4389	0.3321	0.3845	0.0954	0.3058	1.2201	305.8588	79.5464	84.3783
234	2	109.4423	0.6342	0.6361	0.3221	0.0480	4.7812	382.5807	162.1244	80.5556
235	2	41.7990	0.3311	0.2453	0.1725	0.0072	0.8916	245.9024	72.7918	67.4174
236	2	37.6281	0.3301	0.2994	0.0963	0.0224	1.9067	652.8843	23.6675	174.3531
238	2	26.1739	0.0558	0.2215	0.4316	0.0175	1.5160	590.2085	99.3182	267.5030
239	2	32.5887	0.2382	0.2270	0.0595	0.0029	0.8434	326.6206	96.6949	75.9136
240	2	172.1818	0.3287	0.5553	0.0555	0.0089	2.8546	354.0572	78.9340	69.3693
241	2	34.4492	0.3246	0.5128	0.3649	0.0041	1.9016	312.5236	91.0410	69.0187
242	2	23.6465	0.5383	0.2209	0.0469	0.0491	0.7523	261.3106	79.1554	55.8981
243	2	50.1730	0.3198	0.8722	0.0788	0.0059	2.8586	823.0818	259.9802	319.9480
244	2	30.9127	0.7844	0.2879	0.2117	0.0129	2.2200	648.3098	195.4787	261.4897
245	2	35.3640	0.8678	0.2171	0.3318	0.4830	1.2330	383.5766	79.0273	143.7148
246	2	18.2697	0.9063	0.2588	0.4135	0.0190	1.0012	246.0046	65.5198	57.8245
248	2	21.5289	0.5797	0.2641	0.3545	0.0027	1.5142	670.0979	159.3961	90.0407
251	2	30.6306	0.3166	0.1817	0.3090	0.1278	0.1467	167.2602	26.1739	47.0153
254	2	53.3826	1.0542	0.4149	0.5177	0.2321	0.5762	289.0600	79.5473	53.7247
255	2	20.6708	0.5897	0.2083	0.0532	0.0226	1.0579	188.0984	105.7137	43.4248
256	2	21.3559	0.3152	0.4154	0.2473	0.0783	0.6927	295.4452	84.9085	73.0218
257	2	161.9847	0.6013	0.5188	0.1543	0.0048	2.2048	750.4797	24.7698	138.9557
259	2	21.4150	0.0455	0.2363	0.0461	0.0065	0.6409	357.4830	15.0489	76.3798

No.	Group	Al	As	Cd	Hg	Li	Pb	Ca	K	Mg
260	2	37.7342	0.3127	0.6008	0.0409	0.0021	2.4470	1203.9362	43.8878	511.8790
261	2	62.8763	0.3108	0.2639	0.8330	0.0292	1.3226	816.8061	161.2399	372.7324
264	2	72.8070	0.6018	0.5527	0.1885	0.0037	3.3617	393.9715	78.8747	74.6836
265	2	55.1864	0.3068	0.4423	0.4474	0.0178	1.6405	491.0102	89.2439	125.5575
269	2	47.7241	0.0574	0.2892	0.0334	0.0082	1.6444	707.5204	51.8233	136.3958
270	2	19.3065	0.3064	0.2541	0.8009	0.0018	2.0687	265.7193	52.7116	41.5165
272	2	88.5230	0.0618	0.4506	0.0444	0.0173	1.0062	1153.7100	33.7711	244.9834
273	2	67.1542	0.0690	0.4940	0.4363	0.0295	1.2418	267.3401	318.0791	47.8176
275	2	33.6780	0.3025	0.2054	0.4775	0.0100	0.8080	272.2858	55.2081	67.8765
276	2	212.1786	0.0728	0.6499	0.3605	0.0110	1.3634	742.7487	147.8512	250.3068
277	2	12.2361	0.0757	0.3706	0.3948	0.4803	0.5400	458.3539	5.8139	153.0921
279	2	36.4825	0.2987	0.5484	0.2783	0.0001	2.1432	815.9710	122.7777	324.4672
281	2	98.0526	0.8363	0.4358	0.2404	0.0081	1.4669	478.8057	60.0889	72.1802
282	2	54.3851	0.0784	1.1460	0.2959	0.0584	0.5572	265.5870	80.9295	56.0999
283	2	23.0722	0.5619	0.2274	0.1972	0.0146	0.4876	243.9091	118.2531	53.5884
284	2	45.1630	0.6456	0.2059	0.0773	0.0048	0.6417	342.6282	117.7941	91.6674
288	2	57.7600	0.2963	0.6562	0.5100	0.0767	1.1672	501.6407	216.8219	124.4214
289	2	37.7269	0.0800	0.3784	0.5659	0.0313	4.1304	807.0644	31.3390	126.9507
290	2	82.6069	0.2906	0.5321	0.2633	0.0215	1.2093	635.1325	51.8636	98.3577
292	2	29.1044	0.6022	0.2103	0.4874	0.0176	1.1905	318.4755	138.0783	119.1253
295	2	29.1681	0.0809	0.2405	0.2055	0.0930	1.3293	516.5748	68.3075	80.4620
297	2	46.9942	0.2899	0.5909	0.2778	0.0566	1.1003	380.7229	115.9041	81.3881
299	2	29.8698	1.2389	0.4307	0.1777	0.0060	0.4952	283.7432	55.4040	62.3481
301	2	115.3384	0.6257	0.0020	0.1106	0.0807	0.9965	785.3084	134.7449	197.6105
303	2	27.8230	1.0196	0.0020	0.2002	0.0797	1.5203	400.3930	146.9808	175.7886
305	2	29.6773	0.2791	0.2693	0.3002	0.0955	3.5051	257.3552	116.2486	62.3315
307	2	41.2203	0.2759	0.0020	0.2272	0.0611	2.5332	289.4778	143.6178	128.8723
310	2	41.9653	0.0844	0.8870	0.0482	0.0729	1.4192	616.5500	126.4058	257.6134
311	2	93.3628	0.9901	0.8863	0.0521	0.0324	2.2335	315.3200	97.8797	65.1130
313	2	35.7911	0.0981	2.2914	0.0577	0.0562	1.4558	670.5613	79.3805	149.6766
314	2	28.6306	0.9526	0.2379	0.0300	0.1014	4.0042	309.0744	179.8912	71.4543
315	2	53.3158	0.2729	2.2695	0.2594	0.0606	3.1246	674.9833	131.7623	140.7602
317	2	102.2946	1.3672	0.0020	0.0460	0.0741	2.8168	767.0599	176.5439	318.3641
318	2	25.5243	0.2727	0.3265	0.2307	0.0666	17.0699	195.1660	160.0727	34.7362
319	2	33.7552	0.2694	2.2197	0.0429	0.1012	0.4439	629.5433	46.1568	123.1425
320	2	99.4101	0.1001	1.1375	0.2427	0.1287	2.6981	480.2080	286.9859	89.8462
321	2	40.8375	0.1007	0.8524	0.3626	0.0112	1.0531	772.5304	46.8394	128.8656
326	2	63.5800	0.1009	0.0020	0.0583	0.0635	1.7528	419.5851	177.0083	163.2508

No.	Group	Al	As	Cd	Hg	Li	Pb	Ca	K	Mg
327	2	97.7986	0.1374	0.8741	0.3009	0.1605	1.3063	718.3404	21.4716	212.1357
328	2	69.3674	0.6585	0.9498	0.0520	0.0657	1.7487	568.2615	69.1886	114.6441
331	2	48.2755	0.2685	0.8645	0.0304	0.0686	1.5437	417.5492	120.8681	70.8141
333	2	39.0344	0.1694	0.8728	0.0413	0.0805	2.3283	362.1810	91.9027	62.7081
334	2	57.5097	0.4738	0.9161	0.1310	0.0524	0.5461	619.3439	40.4087	130.5427
335	2	38.3804	0.2664	2.4073	0.0407	0.0715	2.0892	713.9125	124.6143	140.1779
336	2	130.6020	0.5541	0.9328	0.1328	0.0767	2.2229	582.7192	139.9729	126.7294
338	2	63.6878	0.2659	1.0334	0.2216	0.0916	3.2170	754.5018	159.5616	170.5405
339	2	22.9514	0.6614	0.0055	0.0599	0.0599	0.9664	544.2800	89.5167	297.5086
343	2	39.9930	0.1056	2.2191	0.0509	0.0650	1.7067	740.6637	46.3898	108.4567
346	2	119.0088	0.1146	0.0020	0.0570	0.0672	3.5081	1240.4954	137.7780	480.0030
348	2	147.3113	0.2643	0.0020	0.0597	0.0588	0.9341	591.6623	142.8859	198.5249
349	2	36.1085	0.1215	0.9814	0.0610	0.0857	0.6178	464.0440	43.1397	81.0963
352	2	54.2411	0.1261	0.8703	0.2841	0.0829	0.7452	694.0121	9.4323	321.4517
354	2	133.7433	0.7280	0.0020	0.0553	0.0861	0.8252	387.3191	217.2396	152.2768
357	2	26.9596	0.7316	0.8975	0.0632	0.0470	0.6987	392.5777	110.8575	97.8846
358	2	23.5596	0.7448	0.8887	0.0354	0.0681	1.8656	278.4385	229.9043	47.4884
359	2	46.6559	0.2633	0.0020	0.0548	0.0925	2.6291	693.1568	307.6772	169.7094
360	2	43.5917	0.2633	2.3121	0.0485	0.0309	0.6692	661.6596	150.0894	116.0784
363	2	21.8395	0.2602	0.8563	0.0407	0.6689	0.5868	353.9612	41.9577	80.5342
364	2	37.9212	1.4335	0.7701	0.0667	0.3184	0.3418	290.2941	55.1095	61.4864
365	2	50.3223	2.9838	2.3530	0.1245	0.0591	0.8984	737.1937	41.0970	137.7428
369	2	24.0665	0.6628	0.1733	0.1882	0.0193	0.1326	200.6753	36.9890	63.6923
373	2	25.3028	0.2512	0.0020	0.3737	0.0590	0.5527	320.2176	124.0248	55.2791
374	2	109.4991	6.8867	0.9274	0.1239	0.0615	0.4731	476.7794	106.2510	112.4585
375	2	23.2381	0.2435	0.8126	0.3648	0.1178	0.1655	282.0744	14.9100	57.9262
380	2	31.6346	0.6868	0.0752	0.0494	0.0858	16.5893	221.3809	95.8629	42.2828
381	2	34.3686	1.5342	2.2586	0.3705	0.0634	1.0104	609.3901	106.0424	99.8476
384	2	31.0648	0.9281	2.1713	0.5242	0.0706	5.1700	643.4517	80.0073	111.9690
388	2	129.7780	0.8611	0.0020	0.4326	0.0633	1.7545	914.3150	313.2107	256.1091
390	2	41.6186	1.0817	0.8672	0.0580	0.0905	1.7688	563.7948	50.9084	221.8070
391	2	25.6192	0.2333	0.8739	0.2927	0.0575	0.7587	429.5526	155.6813	88.2077
394	2	39.3311	1.0783	0.8155	0.4744	0.0430	0.4856	283.8090	45.4467	54.2000
395	2	112.5134	0.6860	0.0020	0.2945	0.0572	2.0750	703.9208	160.6023	175.1758
396	2	25.0918	0.5447	2.1169	0.3713	0.1348	0.3728	574.9732	49.3392	99.5354
397	2	26.9387	0.2317	2.4743	0.1263	0.0911	15.8204	1120.4612	178.2681	237.8934
402	2	71.7139	0.7536	0.2175	0.0675	0.0408	9.8077	568.6336	193.1433	107.6197
403	2	72.2450	1.3205	0.0020	0.0574	0.0447	1.7641	458.2120	414.4526	130.1513

No.	Group	Al	As	Cd	Hg	Li	Pb	Ca	K	Mg
406	2	33.5593	0.2181	0.0551	0.2125	0.0656	3.3779	276.0575	92.2092	51.4397
407	2	40.6666	0.2240	2.6977	0.0498	0.0606	22.0647	462.1506	516.3364	90.0873
408	2	28.8490	0.7597	0.0020	0.0475	0.0703	0.6401	349.5659	199.5901	80.5873
409	2	176.1701	0.6715	0.2768	0.2700	0.0707	9.9978	959.2837	369.8130	271.2649
411	2	55.0437	0.6996	0.0020	0.4483	0.0861	1.6394	958.6891	157.2347	307.8734
412	2	121.0121	0.2257	0.2241	0.0489	0.0832	7.1596	1636.4753	394.8769	497.6298
417	2	46.3231	0.1282	0.0123	0.2437	0.0996	1.3208	410.5570	347.3017	78.4749
419	2	70.4234	0.7592	0.0787	0.0724	0.0743	14.2320	482.8273	202.6899	95.8610
421	2	97.0536	0.2238	0.1311	0.1964	0.0642	16.9853	442.4923	127.1050	91.2628
423	2	45.2269	0.8122	0.0020	0.1485	0.4650	4.0501	260.6840	130.3094	42.7348
428	2	63.2510	0.8231	0.0020	0.3141	0.0678	2.8793	938.8830	276.3207	247.1746
430	2	50.9469	0.1339	0.0020	0.0673	0.0922	2.2485	673.4761	166.3521	388.4113
433	2	50.4302	0.7708	0.0814	0.1026	0.0109	5.4641	1197.1023	25.7739	512.1071
437	2	46.9616	0.7754	0.0020	0.4210	0.0750	0.7444	371.5762	124.6124	126.8280
438	2	61.3872	0.8717	0.0688	0.2789	0.0704	5.3405	222.9711	187.0148	47.3250
439	2	30.3786	0.9185	0.0208	0.1610	0.0496	1.3942	268.4387	230.7289	74.5176
440	2	37.9518	0.1359	0.0273	0.1435	0.0913	2.3571	487.0233	105.1565	111.2307
441	2	37.3823	0.9256	0.8881	0.3056	0.0724	2.1321	320.1000	328.0224	56.0946
443	2	47.3576	0.7860	0.0175	0.4305	0.0836	1.6097	368.8239	88.7539	100.1788
444	2	58.6731	0.1414	0.8836	0.4688	0.0400	1.4457	551.1386	100.4850	75.7667
445	2	76.0026	0.8285	0.1314	0.3712	0.0494	5.4170	544.0097	271.1393	130.0846
446	2	327.7310	0.2138	0.2476	0.3356	0.0612	8.7790	504.1414	314.3924	179.2196
449	2	37.4755	0.1886	0.0358	0.2118	0.0632	4.4464	1460.8717	120.9225	738.9545
453	2	52.5801	0.1854	0.1094	0.5705	0.0704	1.6739	1986.0411	62.3804	259.6087
457	2	162.7107	1.5035	0.7840	0.0819	0.0511	0.8578	588.5137	50.3086	84.0360
460	2	62.3655	1.8314	0.0442	0.5749	0.0679	3.0366	659.3354	90.8216	81.5979
461	2	49.7716	2.1139	0.0020	0.4386	0.0748	1.7880	288.6844	174.9110	83.3838
463	2	76.6079	0.1815	0.9299	0.1145	0.0382	1.0432	798.8045	24.2868	116.6364
465	2	38.8969	0.1812	0.0020	0.1534	0.0760	0.8784	288.3328	156.3032	65.6285
472	2	84.6189	3.9764	0.0020	0.0476	0.0692	1.5684	926.2253	102.5982	198.1875
473	2	31.8948	1.8895	0.0020	0.3020	0.0544	1.4048	284.6229	448.0986	85.1077
475	2	32.7220	0.1495	0.0020	0.0366	0.0022	0.6879	243.0383	118.0017	65.6331
476	2	88.0918	0.9609	0.9032	0.2714	0.0052	1.1183	504.5348	61.9790	111.7271
477	2	43.6294	0.9194	0.0020	0.1306	0.0535	1.8374	314.5646	59.3754	72.1793
479	2	85.5014	0.6102	0.8435	0.1631	0.0165	1.2472	408.8445	51.1032	75.7330
480	2	15.5450	0.1705	0.8716	0.1851	0.0610	0.7561	233.3598	9.2191	44.3173
481	2	67.6653	0.9827	0.1650	0.0327	0.0673	1.7995	973.9266	87.0821	387.3439
482	2	32.6424	0.1690	0.2912	0.0489	0.2526	2.3257	321.3065	254.0547	74.7250

No.	Group	Al	As	Cd	Hg	Li	Pb	Ca	K	Mg
484	2	75.1193	2.5348	0.0958	0.0473	0.0670	7.6273	341.0645	261.5202	69.7155
489	2	21.7988	0.1579	0.0020	0.0317	0.0240	0.5057	225.4725	54.3535	54.1136
490	2	57.2818	0.1687	0.0020	0.0480	0.1185	2.2151	566.1561	236.2772	182.6486
498	2	54.3857	0.5706	0.0087	0.0181	0.0522	2.2789	676.3976	62.1026	96.0141
499	2	36.4164	0.1627	0.9265	0.0175	0.0198	1.8280	393.3539	152.9621	104.4484
500	2	77.4891	0.1611	0.1516	0.0384	0.0645	4.8133	912.6154	153.7943	371.4150
503	2	37.5808	1.0337	0.1885	0.0104	0.0705	0.5852	401.6770	44.3567	110.5934
504	2	74.2539	0.1580	0.5706	0.0797	0.0583	6.8553	449.3617	43.9624	206.0106
514	2	27.6383	0.7034	0.3861	0.0597	0.0469	0.4756	304.4211	35.3981	84.8537
515	2	51.5936	2.7816	0.8855	0.0010	0.0033	3.6258	463.6117	179.1828	85.1321
516	2	20.9859	0.8231	0.3204	0.0527	0.0686	0.9677	258.0933	107.0503	53.8088
519	2	18.0465	1.7409	0.4763	0.0500	0.0118	2.3728	357.3096	97.1314	55.7272
520	2	30.8017	0.9413	5.4400	0.0118	0.0901	2.3684	512.2580	70.2674	154.6360
521	2	28.8612	1.0877	0.4719	0.0146	0.0319	1.5388	418.8800	178.4344	89.5038
522	2	22.2669	0.1697	0.0236	0.0629	0.0806	1.5832	373.7897	65.1386	86.3190
523	2	46.7419	0.1699	0.5104	0.0196	0.0782	0.4615	669.0486	55.0604	136.0780
524	2	30.9137	0.5019	0.3505	0.0235	0.0721	0.7263	660.9707	37.8098	81.4010
525	2	47.5098	1.9682	0.4710	0.0642	0.0395	2.2379	699.6356	284.1736	228.9049
528	2	23.5944	0.1545	0.3524	0.0260	0.0558	3.3002	405.0061	137.3045	136.9083
529	2	57.1666	0.1729	0.5044	0.0415	0.0345	3.8072	712.9290	217.3274	183.5092
530	2	50.0929	0.1513	0.8834	0.0488	0.0395	1.1332	1889.3194	35.7615	613.2491
531	2	46.8497	0.1341	0.4578	0.0472	0.0229	2.6338	1044.8540	168.2283	350.6902
532	2	314.0974	0.1186	0.4896	0.0039	0.0446	0.8409	286.3597	175.9251	66.8584
533	2	61.4178	0.8981	0.4664	0.0084	0.0420	0.7670	793.2665	93.5433	228.5592
534	2	133.0934	0.0884	0.0020	0.0176	0.0769	1.2593	833.7958	204.5553	507.4893
538	2	17.2549	1.1471	0.3322	0.0506	0.0859	2.2979	285.5493	91.9422	66.4482
539	2	43.3699	1.1835	0.4678	0.0255	0.0545	1.4730	1311.9299	42.1275	531.2304
541	2	37.6969	0.1797	0.9756	0.0075	0.0065	3.3607	542.4913	105.0611	173.4305
542	2	23.5518	0.1886	0.4217	0.0419	0.0647	1.5251	562.5443	62.0843	179.0444
543	2	24.9385	0.0854	0.4522	0.0329	0.0024	4.5725	300.6953	65.8670	71.0305
545	2	37.6950	1.2596	0.4715	0.0407	1.0009	1.5814	292.8441	84.4897	60.8784
546	2	15.1711	0.0851	0.3863	0.0254	0.0062	1.2644	267.4463	109.4864	53.4463
547	2	20.3082	0.1928	0.3602	0.0130	0.0349	1.1087	613.7065	17.4063	321.0817
548	2	13.9712	1.3540	0.3398	0.0561	0.0641	0.8799	263.7822	47.9047	60.2296
549	2	50.3090	0.0825	0.3654	0.0405	0.0889	0.6719	277.2852	56.8228	75.5878
551	2	24.6464	0.0653	0.4797	0.0250	0.0038	2.9623	391.6274	88.5624	78.4851
552	2	16.1869	0.0642	0.3814	0.1776	0.0933	1.1009	264.1632	74.4147	51.9940
553	2	26.1083	0.0589	0.4433	0.0326	0.0604	0.8013	330.2714	128.8385	80.5010

No.	Group	Al	As	Cd	Hg	Li	Pb	Ca	K	Mg
554	2	13.4473	0.0548	0.3545	0.0398	0.0299	0.6218	443.5296	113.6118	125.9211
556	2	20.8039	0.9214	0.8929	0.0745	0.0216	0.4452	303.2786	42.3863	75.4918
558	2	22.3222	0.1928	0.8024	0.0577	0.1002	0.7536	380.8556	70.5999	83.6146
559	2	16.1941	0.0325	0.1698	0.0470	0.0669	0.0949	170.5809	32.7676	54.6465
560	2	17.8225	0.5927	0.3680	0.0501	0.0942	0.5300	296.9155	24.2630	79.4858
561	2	28.2433	0.0078	0.0813	0.4733	0.0668	2.2134	413.6415	202.7388	174.0461
565	2	37.7432	0.6066	1.3697	0.0434	0.0228	0.8558	784.4571	80.9820	351.7599
702	1	30.1632	1.3632	0.0020	0.0070	0.0257	0.0200	24.8571	20.9418	56.6433
703	1	37.1555	0.1966	0.0326	0.0056	0.0033	2.4456	1409.9962	142.9676	201.6493
704	1	37.3053	0.1996	0.1459	0.0123	0.0352	4.4301	809.8066	72.2589	119.1636
705	1	28.0315	0.2009	0.0021	0.0055	0.0276	1.4717	476.8380	48.2487	83.4503
706	1	160.4980	0.2106	0.0057	0.0257	0.0103	0.6931	1112.0913	117.5440	277.2839
709	1	44.4995	0.2117	0.0441	0.0371	0.0295	4.6590	1678.6231	32.3204	134.1279
710	1	25.0779	0.2202	0.0361	0.0080	0.0233	1.0976	2129.9440	56.4158	331.0284
711	1	33.8150	0.2283	0.0034	0.0167	0.0257	0.7477	404.2212	92.2793	83.5129
712	1	45.3873	0.2296	0.0652	0.0047	0.0713	2.8420	2110.4923	72.4616	240.3369
713	1	37.1836	0.2317	0.0194	0.0444	0.0446	1.0557	468.6501	177.7823	102.2073
722	1	44.9477	0.2319	0.8325	0.0203	0.1013	0.2881	505.8113	149.8828	116.0235
723	1	39.3462	0.2349	0.0499	0.0064	0.0165	2.3727	1033.6086	64.4274	105.6227
724	1	33.6388	0.2454	0.0264	0.0446	0.0558	1.6841	1342.6021	45.4418	158.9453
725	1	107.4590	0.2504	0.0673	0.0289	0.0130	3.0003	495.6484	260.2701	84.3618
726	1	58.7212	0.2512	0.0577	0.0086	0.0306	5.8410	456.8694	56.8833	61.0411
727	1	19.3399	0.2570	0.0083	0.0461	0.0296	1.5898	603.3410	119.3328	107.6008
728	1	33.4381	0.2595	0.0684	0.0151	0.0107	1.8226	2135.1122	46.3400	341.6694
729	1	33.4819	0.2609	0.0803	0.0164	0.0427	1.7211	316.0829	208.5196	61.1326
730	1	30.7287	0.2666	0.1453	0.0284	0.0453	3.4013	1036.7260	184.2884	194.0203
731	1	18.2050	1.5993	0.1574	0.0237	0.0136	1.3548	249.8353	77.6688	52.6846
734	1	26.8871	1.6973	0.0455	0.0476	0.0517	2.2169	1038.8469	194.0898	244.8571
735	1	43.5081	0.2789	0.0939	0.0503	0.0464	3.1461	1101.0212	181.6324	194.1522
739	1	50.1624	1.9481	0.0331	0.0039	0.0259	3.3713	2118.1141	143.7583	407.3467
740	1	22.1333	0.2799	0.0359	0.0289	0.0026	2.9674	334.5423	185.0030	56.4838
742	1	54.2249	2.5147	0.0085	0.0400	0.0474	1.2244	723.0266	163.1685	229.2873
744	1	17.6991	0.3018	0.0344	0.0362	0.0347	3.1190	1574.2119	92.5902	262.1883
745	1	37.1626	0.3062	0.0272	0.0469	0.0187	1.9304	351.0040	216.5768	58.6991
746	1	74.9409	0.3133	0.0254	0.0559	0.0028	2.1742	1039.8473	98.0296	243.9366
747	1	45.1081	0.3258	0.0020	0.0301	0.0307	1.9432	2307.4950	53.6918	242.1009
748	1	47.1230	2.8553	0.0254	0.0322	0.0083	1.3745	2497.9486	148.7519	717.7123
755	1	41.4079	0.3363	0.0856	0.0156	0.0196	3.2464	1019.9067	42.6366	136.4108

No.	Group	Al	As	Cd	Hg	Li	Pb	Ca	K	Mg
760	1	31.0716	0.3387	0.0566	0.0662	0.0350	2.4236	844.4782	286.5532	100.0150
761	1	41.4945	0.3430	0.0295	0.0525	0.0213	3.4900	793.6086	221.7625	199.0402
762	1	20.4255	3.4155	0.0144	0.0375	0.0490	1.2230	2508.9852	141.7800	396.3217
766	1	37.6240	0.3641	0.0083	0.0045	0.0166	1.3059	1353.2679	32.6987	230.5579
767	1	51.3163	0.3745	0.0973	0.0231	0.0226	5.7081	2222.9674	32.9051	491.6021
768	1	33.7142	0.3957	0.0304	0.0457	0.0105	1.5458	531.5730	103.6551	63.4241
769	1	28.7892	0.4020	0.0071	0.0042	0.0476	1.1312	310.5845	145.2454	66.5810
775	1	28.3017	0.4102	0.0020	0.0370	0.0586	0.0200	339.0461	53.6154	75.4644
777	1	23.6249	0.4284	0.0131	0.0488	0.0157	0.7827	262.3065	101.4334	57.6418
780	1	59.1381	0.4476	0.0020	0.0555	0.0137	1.4848	537.3551	295.6597	149.6484
781	1	39.0124	0.4698	0.3085	0.0055	0.0132	3.7612	982.3794	118.1495	416.1067
782	1	40.5615	0.4724	0.0906	0.0384	0.0456	3.1126	1432.0054	246.3312	279.0620
785	1	27.5991	0.4758	0.0222	0.0310	0.0053	1.5493	1112.8721	57.7757	121.9571
787	1	39.1935	0.4851	0.1019	0.0130	0.0305	2.4092	1352.1204	90.8290	204.7747
788	1	28.0403	0.4896	0.0171	0.0283	0.0228	1.3806	838.5122	41.1025	149.5813
789	1	22.4286	0.4920	0.0659	0.0371	0.0855	2.0228	1240.5537	137.3055	217.0126
790	1	58.6541	0.5163	0.0518	0.0182	0.0441	2.2313	917.5756	84.0015	197.3659
791	1	26.0545	0.5191	0.0128	0.0281	0.1026	1.1082	330.5851	106.9247	57.8956
794	1	25.0367	0.5207	0.0003	0.0149	0.0226	10.3081	1021.3453	32.9632	94.9539
795	1	35.7943	0.5259	0.0145	0.0434	0.0175	0.9635	1794.6340	99.8328	233.2237
799	1	15.6085	0.5261	0.0375	0.0162	0.0522	2.8756	428.7032	177.2042	146.4723
800	1	28.3826	0.5383	0.0141	0.0108	0.0090	1.4232	282.6392	174.1098	67.6509
801	1	34.2116	0.5395	0.0221	0.0563	0.0425	1.3238	1780.3981	40.1202	289.2688

No.	Na	P	B	Mn	Ni	V	Co	Cr	Cu	Fe
3	657.2065	196.2461	0.7611	2.2827	0.0080	0.0161	0.0317	2.9233	6.9830	76.3811
6	999.6106	210.2530	4.4503	12.0932	0.2090	0.0427	0.0325	3.4922	9.2273	61.1153
7	1236.8520	233.2598	1.2850	7.0887	0.3975	0.0788	0.0331	5.0617	10.8207	58.0489
8	241.8306	205.7450	2.2030	3.8759	2.7918	0.0699	0.0384	4.3892	14.2610	119.7725
10	442.7290	222.5294	0.5571	3.0867	0.2222	0.0436	0.0041	5.0138	12.3898	100.2070
13	107.5495	319.3020	1.3792	3.3070	0.1686	0.1045	0.0075	5.2633	10.9647	86.7719
15	836.7084	278.7499	0.4833	3.0311	0.6324	0.0020	0.0177	4.7834	19.8632	63.8989
16	372.3009	216.1730	2.4700	5.6126	0.6010	0.1159	0.0078	4.8299	16.8624	48.9817
17	621.3556	340.1211	1.5852	5.9853	0.4222	0.0847	0.0078	5.6536	14.3427	79.8697
20	128.8221	156.4207	2.7312	1.0495	0.0005	0.0020	0.0114	4.7755	5.0998	102.7060
21	401.4609	151.7378	1.0863	4.9545	0.0005	0.1460	0.0532	2.9298	5.9731	75.4168
22	327.8185	157.5594	0.9654	1.3520	0.0988	0.0020	0.0035	3.4778	6.1320	52.7417
23	338.1930	233.7953	0.8911	0.8490	0.1308	0.0172	0.0262	4.4330	10.6898	89.7099
24	422.0456	223.4090	1.2852	3.4142	0.1037	0.0441	0.0044	4.7864	11.7403	87.1575
26	292.9999	196.9792	0.0923	1.5878	0.0832	0.0204	0.0302	4.4495	7.2120	114.3000
27	353.6348	174.2079	2.2298	3.1333	0.0769	0.2477	0.0366	3.5994	9.3833	83.9061
28	297.6989	131.4205	0.8628	3.8284	0.0005	0.0406	0.0161	2.7142	5.6172	109.1584
29	346.1597	245.6256	0.9668	8.2796	0.4787	0.1679	0.0487	7.5147	7.9939	115.3248
31	317.3178	157.5694	0.3401	2.7750	0.0782	0.2154	0.0378	3.2023	9.1518	61.6774
32	412.2309	256.3314	1.7045	7.3053	0.3447	0.0856	0.0489	5.5965	8.3903	59.1535
34	373.3213	338.4647	1.6692	3.6323	0.0883	0.1307	0.0343	5.7077	13.9521	153.8588
37	270.0892	172.3506	0.8593	3.3106	0.0005	0.0592	0.0434	3.2127	8.0587	109.7885
39	543.4406	224.7439	6.2775	3.0844	0.2794	0.0216	0.0028	3.8775	8.5556	84.7506
40	429.0475	254.5023	0.6083	1.8057	0.4841	0.0041	0.0214	4.0354	10.0600	71.6114
41	386.1643	298.9676	4.0827	4.5033	0.3421	0.0365	0.0194	5.4773	11.8843	94.9548
42	367.2404	227.9239	0.4515	4.0202	3.0940	0.0093	0.0070	4.3230	11.6586	91.9060
44	605.9659	155.7939	5.2827	6.8530	0.2190	0.0785	0.0045	3.3868	8.8977	62.8352
46	282.5181	262.6968	1.2511	0.9754	0.2037	0.0176	0.0453	4.6611	8.3108	156.7565
47	514.2964	197.4913	0.4000	2.4247	0.1251	0.0694	0.0380	6.4287	7.5091	73.8534
49	325.8491	258.3223	1.1050	10.3766	1.9032	0.0467	0.0414	6.4517	22.2420	70.8594
50	463.9483	154.3484	3.3548	5.2240	0.1937	0.0358	0.0314	3.5500	10.0265	245.7804
51	550.5661	208.3855	0.4631	2.0530	0.3863	0.0596	0.0224	4.1991	7.3782	98.4310
53	302.1139	205.1624	0.5852	5.4835	0.4600	0.0568	0.0604	4.2486	9.9436	86.7821
54	284.5894	167.1231	0.4577	6.1738	0.3822	0.6691	0.0427	3.9073	8.9137	41.7119
56	337.2666	239.8057	0.4367	18.9718	0.1899	0.0837	0.0339	4.4858	11.4507	243.6998
57	431.5022	133.8093	1.6117	18.9238	0.0005	0.0931	0.0128	3.5337	7.7481	65.7886
59	308.5045	117.7145	0.1227	2.6733	0.1207	0.0098	0.0305	3.2440	3.2994	140.8256
62	275.3412	159.1664	1.1810	1.3285	0.1146	0.0020	0.0417	2.7614	4.7731	77.5888

No.	Na	P	B	Mn	Ni	V	Co	Cr	Cu	Fe
64	288.9021	256.5951	0.4531	0.7051	0.8879	0.0001	0.0905	4.0123	7.8132	71.5026
66	374.1112	252.8632	0.6142	22.5565	5.3043	0.5389	0.0388	5.1943	19.2982	102.6267
67	320.8752	311.7139	0.4979	0.9297	0.1274	0.0059	0.0358	4.6513	8.9190	86.5763
68	359.6113	200.7023	0.9110	4.1666	0.2634	0.0438	0.0410	3.9890	9.8464	106.2166
69	523.7697	314.7489	1.0227	10.9531	0.4751	0.1989	0.0458	6.8951	14.7511	118.7988
71	427.9740	266.5436	0.7633	1.1154	0.1534	0.0017	0.0264	4.4511	9.4909	131.3996
72	337.2456	208.7891	1.4977	5.8077	0.8052	0.1095	0.0491	3.6414	9.9967	62.8908
73	505.9184	139.3731	0.4862	4.3621	0.0005	0.1096	0.0308	2.8545	5.5627	59.3514
74	314.1076	236.5215	1.8249	2.8778	0.1748	0.0724	0.0047	5.4780	8.7510	69.0113
75	460.5328	208.5737	4.5896	2.8662	0.5328	0.0020	0.0556	4.2833	10.3457	78.6955
76	197.6378	145.6200	1.8988	1.4119	0.4023	0.0020	0.0237	2.6196	5.6459	103.2539
78	430.0464	236.4015	1.4021	11.5787	0.3032	0.0452	0.0010	4.8892	10.4062	208.4310
79	329.5086	273.0080	0.1552	6.8706	0.1299	0.1578	0.0291	6.0333	15.1052	139.3608
81	341.2054	170.1990	0.5190	1.1823	0.0005	0.0480	0.0236	3.2458	8.0935	120.2660
82	463.9732	264.0260	0.4141	5.5388	0.2433	0.0889	0.0403	6.2480	13.1325	65.2103
83	527.1511	288.1559	0.9898	10.3717	0.4585	0.0608	0.0288	6.5539	15.2967	61.1825
84	192.8996	123.1918	1.8086	1.1518	0.0005	0.0048	0.0334	2.3721	6.2833	89.4820
85	930.3890	253.3401	1.3699	3.3862	0.2479	0.0487	0.0527	4.3350	5.8086	60.6261
86	500.4941	250.1624	0.2399	4.8627	0.4073	0.0727	0.0161	5.2074	11.1147	224.1713
89	1615.5926	308.3235	1.5786	21.1966	0.3295	0.1376	0.0429	6.1278	21.3130	93.9555
90	564.6399	259.5570	0.6008	9.2851	0.3989	0.0741	0.0532	4.7781	12.6932	60.7535
92	532.4194	195.5549	1.2586	4.2557	0.1196	0.0251	0.1927	3.7880	7.1063	90.8791
93	466.6912	249.4336	1.2711	9.0412	0.1083	0.0928	0.0555	5.0994	11.7546	213.5212
94	251.1330	130.6932	1.1751	2.8316	0.3700	0.0084	0.0441	3.6141	6.0868	111.0921
95	459.1776	299.5886	1.0890	6.6809	0.3067	0.1005	0.0032	6.9085	17.1438	66.9582
96	2432.8630	258.5320	1.0534	68.1624	4.7524	0.2454	0.0488	6.3431	99.7464	266.4715
97	797.9090	231.8413	12.5338	1.0585	0.0474	0.0221	0.0147	3.9985	7.5373	73.5212
98	435.6966	274.3388	1.4603	19.3784	0.2327	0.1965	0.0063	6.8517	14.6414	83.4412
100	378.0350	270.7045	2.3131	9.3880	1.4687	0.1517	0.0133	5.9534	15.1885	99.5865
102	138.1776	100.7849	0.3565	1.6647	0.0005	0.0020	0.0026	5.1412	3.6024	197.0777
103	285.0398	283.1455	0.0185	7.7127	0.4803	0.1097	0.0546	4.4710	12.0651	96.5635
105	243.8331	145.3850	1.6702	5.6180	0.2786	0.0599	0.0056	2.8777	8.4001	232.7592
107	268.2122	231.4859	0.8888	5.6017	0.2044	0.0020	0.0137	5.2384	12.4620	88.8602
109	345.0123	153.0202	3.9250	2.4332	3.9445	0.0380	0.0282	3.3106	6.6362	75.1266
111	223.5244	81.0091	0.6726	3.2659	0.0005	0.0020	0.0123	1.8130	2.5885	205.1209
119	317.7661	105.6981	0.5406	3.1710	0.0005	0.0020	0.0211	2.3091	3.7276	77.3630
124	832.2145	284.0828	1.0889	1.4704	0.2764	0.0696	0.0347	5.3878	11.1180	289.0219
126	671.3465	201.6445	0.6192	15.8774	0.0005	0.1443	0.0382	4.2063	8.4528	282.1140

No.	Na	P	B	Mn	Ni	V	Co	Cr	Cu	Fe
127	198.2212	62.6206	1.4764	0.4070	0.0005	0.0020	0.0020	1.5470	2.0124	96.5421
128	148.5685	123.1007	1.1894	0.6760	0.0005	0.0020	0.0061	2.7008	4.7189	109.0206
129	382.6885	202.8747	2.3749	18.4894	0.5973	0.1076	0.0308	4.7597	15.5938	57.3859
130	1585.1269	284.4744	0.4323	1.3105	0.2453	0.0167	0.0038	3.9306	10.5302	45.7035
131	491.3793	215.0107	2.6669	3.6861	0.3039	0.0020	0.0242	3.4797	9.0856	149.5650
132	420.8026	283.4772	2.5190	1.4612	0.6132	0.0020	0.0448	5.8615	11.5272	59.4229
136	168.7593	87.8160	1.5713	0.7755	0.0005	0.0020	0.0040	1.7344	2.4179	41.8872
138	262.6702	72.2767	0.9235	1.0709	0.0005	0.0020	0.0269	1.3629	2.2480	84.2877
139	391.2631	178.9979	0.0728	3.6994	1.0755	0.0147	0.0389	3.3547	7.3548	89.5778
140	314.1015	253.4872	1.1702	5.3477	0.7231	0.1535	0.0339	4.5655	26.5130	273.2277
145	970.6858	223.2886	2.8320	3.7787	0.0442	0.0301	0.0116	3.6251	8.0199	109.3047
148	330.5566	208.9415	0.1188	5.6807	0.5866	0.0732	0.0192	15.5832	12.4765	87.6487
150	297.0376	233.7935	1.1051	4.9832	0.9629	0.0513	0.1160	6.2014	13.7376	206.8300
152	303.3234	178.7918	4.3931	0.9556	1.0253	0.0020	0.1060	3.1942	10.1726	92.5451
155	327.5127	196.5358	0.4081	1.2881	0.6862	0.0048	0.0459	3.6716	10.3340	57.4535
158	367.4888	236.7121	2.0483	7.1180	0.6633	0.1211	0.0192	5.4779	22.7320	60.5229
159	641.8414	309.6596	0.5219	8.1977	0.5084	0.0709	0.0442	6.0550	13.7568	75.8677
163	432.2693	230.8835	0.9234	2.9313	0.3921	0.0654	0.0531	5.2435	12.5400	253.5651
165	428.4993	124.0888	2.1949	1.0993	0.3841	0.0020	0.0408	2.4211	8.1084	200.1920
167	5297.8004	378.4934	0.6278	12.8741	0.5107	0.3878	0.0653	6.4532	18.9755	127.3177
169	335.4072	295.6968	2.0417	11.8157	1.0461	0.0952	0.1698	6.3435	12.4127	98.4997
170	294.1655	197.7159	2.5176	1.3467	0.0005	0.0020	0.0394	2.9646	7.5799	71.0879
173	301.3388	240.1794	0.1269	3.0588	0.4033	0.0244	0.0085	4.7355	14.5279	102.7342
174	310.3466	117.1347	3.9701	2.3821	0.0005	0.0020	0.1313	2.4524	4.8444	138.0957
175	203.2551	104.0285	1.3506	3.8467	0.4293	0.0099	0.0542	5.1986	3.0798	103.3631
177	911.9820	272.2028	0.8262	4.1090	0.3278	0.0853	0.0072	5.8368	12.1370	182.3052
178	664.2754	236.6554	1.0660	1.9369	0.2000	0.0190	0.0309	3.3747	12.1746	61.4458
183	577.1506	173.7235	0.5002	3.8881	0.0005	0.0590	0.0055	3.6322	6.7068	82.0583
187	346.8381	241.2425	1.5902	3.4375	2.1687	0.0264	0.0526	5.4937	16.5886	37.2151
189	351.6646	275.6968	2.5290	2.1876	2.3598	0.0207	0.1377	12.5676	7.8541	121.2450
191	2284.3795	259.4486	0.4998	9.0001	0.5054	0.1313	0.0065	5.9948	13.5131	191.3976
192	939.3099	412.7789	0.6539	5.3348	1.3317	0.1151	0.0145	7.1029	15.7837	69.9472
194	348.1406	160.3738	2.3420	5.7860	1.5896	0.0533	0.0486	3.1512	9.8612	60.3321
196	227.4944	194.5392	1.0005	5.2043	0.3592	0.0606	0.0271	3.3214	6.5528	46.8432
199	319.8560	217.5915	2.3331	4.8132	0.2048	0.0589	0.0194	3.6680	9.4634	61.8700
200	472.8611	204.4912	2.7513	32.8603	0.3335	0.1532	0.0056	3.5472	9.4135	77.3019
201	79.3306	165.7895	2.5641	6.2720	0.0611	0.0363	0.0268	4.2246	6.8425	106.1528
203	576.5272	183.1263	0.2429	1.3514	0.0005	0.0059	0.0597	2.8482	5.0068	201.0647

No.	Na	P	B	Mn	Ni	V	Co	Cr	Cu	Fe
204	126.7525	185.5725	0.7054	2.1822	0.0862	0.0020	0.0305	3.4655	5.1823	188.2076
205	90.9916	214.9464	2.2182	0.9003	0.8390	0.0004	0.0349	6.8963	4.2241	222.6587
206	106.9078	134.5403	3.9020	4.3818	0.0005	0.0427	0.1303	2.9310	14.5787	202.0174
209	92.7999	149.8692	0.7234	4.3979	0.0005	0.0941	0.0512	2.1804	5.6787	78.9363
211	427.5905	203.8173	0.5383	6.8434	1.4708	0.0452	0.0365	3.8830	6.7386	52.4785
213	100.0535	150.6704	0.7089	0.8597	0.0005	0.0020	0.0349	2.2484	4.0263	40.7359
214	269.9685	178.7026	0.9884	4.8931	0.0447	0.0020	0.0132	6.8137	6.6456	37.4902
215	172.8820	141.2103	2.2427	1.8803	0.0005	0.0020	0.0305	2.6736	5.8858	117.4588
217	128.7846	166.9444	1.0997	2.2108	0.1889	0.0020	0.0222	3.4915	7.0470	129.2297
219	78.5759	107.5665	0.4255	2.5328	0.0005	0.0020	0.0506	2.2299	9.4313	51.6713
220	98.2965	289.0464	1.1929	0.1234	0.0005	0.0020	0.0287	0.0522	5.2067	135.0193
221	847.6003	297.4810	1.5918	5.6830	1.3114	0.0466	0.0794	12.2457	7.9110	68.0513
224	267.8021	220.0674	0.3624	2.4162	0.0005	0.0084	0.0381	2.4561	5.4599	113.3807
226	126.1327	261.5115	3.1801	1.6352	0.0264	0.0020	0.0219	4.5608	6.9966	168.6650
227	98.4802	202.7253	0.7202	1.6369	0.0005	0.0682	0.0354	2.7071	10.1716	79.6443
229	161.8359	203.9953	2.8186	2.3809	0.2788	0.0020	0.0328	8.1291	6.5828	74.9308
231	106.5039	159.0547	0.6102	1.0645	0.0005	0.0020	0.0353	2.6178	4.5155	45.2750
232	112.9466	128.6815	1.0474	1.2841	0.0005	0.0020	0.0122	2.0087	4.8674	132.7093
233	156.9144	187.7064	3.8075	1.4797	0.0005	0.0020	0.0449	3.0441	6.5545	47.8578
234	176.9823	160.6471	0.5674	23.6045	0.3007	0.1745	0.0754	3.7265	20.0960	171.7996
235	110.8972	198.2882	3.8247	1.1809	0.0005	0.0289	0.0786	4.0749	6.6369	57.3340
236	115.9689	187.1756	2.2999	3.3721	0.3420	0.0367	0.0586	2.5845	15.0412	79.9464
238	376.8644	131.3470	0.2306	5.7429	0.0005	0.0145	0.0080	2.3884	4.6698	97.0681
239	303.2999	125.6553	0.0041	5.3942	0.0005	0.0111	0.0449	2.3083	4.8431	204.4560
240	304.2279	215.3539	1.6261	2.2140	0.0076	0.0013	0.0003	3.8996	7.1607	79.7515
241	263.9712	253.1903	0.2267	1.6265	0.0005	0.0020	0.0146	3.9002	8.5868	50.4240
242	271.1263	171.0805	3.9247	1.3123	0.0005	0.0020	0.0310	2.7707	6.3700	211.8989
243	635.9333	161.7905	1.4060	10.1384	0.2820	0.0726	0.0301	4.2580	7.9652	66.0094
244	791.8545	204.2950	0.8565	4.3945	0.0846	0.0421	0.0495	4.2774	6.4856	179.4067
245	119.8725	140.5676	0.5443	3.3520	0.1739	0.0020	0.0520	2.3958	4.5147	207.2470
246	96.2710	172.0587	0.9841	0.8473	0.0005	0.0020	0.0558	2.9012	5.0099	88.4302
248	599.2159	184.2462	1.7645	10.7511	0.0005	0.0425	0.0534	3.2589	7.6236	164.4354
251	80.2555	118.6808	2.6409	0.4095	0.0005	0.0020	0.0349	1.9085	3.9932	92.1423
254	233.5703	179.6866	4.1701	0.9076	0.0005	0.0020	0.0029	3.2156	7.2425	201.7403
255	414.7904	182.1457	2.6997	1.1679	0.1254	0.0024	0.1014	5.8108	5.4334	120.9575
256	273.7960	184.9599	2.2871	0.6680	0.0005	0.0020	0.0468	2.3803	5.5945	59.4010
257	131.8743	225.5225	3.4989	1.5270	0.2782	0.0394	0.0201	4.0815	9.7493	162.6811
259	56.6043	104.7259	4.4521	0.5809	0.0005	0.0020	0.0378	1.7082	11.1067	86.1855

No.	Na	P	B	Mn	Ni	V	Co	Cr	Cu	Fe
260	392.0342	229.6901	0.2809	3.2783	0.4167	0.0461	0.0289	4.3065	6.2427	54.1085
261	539.3166	161.2685	1.1097	8.5127	0.0005	0.0349	0.0218	3.1382	14.6955	78.5800
264	166.9045	163.6298	0.6724	7.9494	0.9064	0.1063	0.0189	4.9234	8.1013	235.2557
265	159.2561	179.8851	1.0832	1.8474	0.0592	0.0020	0.0922	3.5107	10.5674	41.3529
269	111.6934	131.1559	2.4534	15.4027	0.4805	0.0020	0.0507	4.8902	10.8389	30.7819
270	145.5383	148.4244	0.5174	2.0439	0.0005	0.0144	0.0028	2.6051	6.0087	95.5870
272	164.0199	215.9712	3.2754	4.5107	0.3650	0.0020	0.0353	4.0710	12.3631	46.1447
273	593.2854	207.9745	1.2745	1.0227	0.0005	0.0020	0.0763	3.5709	7.6989	56.4177
275	209.0316	144.1308	1.5048	1.6222	0.0005	0.0020	0.0235	2.1855	6.1539	133.9985
276	285.1865	250.0809	0.6517	3.8410	0.4220	0.0858	0.0355	4.6079	11.6686	51.1770
277	67.5173	147.2992	0.2400	1.1180	2.3619	0.0020	0.0450	2.1745	6.7365	74.9508
279	541.3127	206.7533	0.4276	3.0555	0.1620	0.0020	0.0179	4.3008	10.2929	44.0631
281	124.3893	190.2319	1.1998	2.5446	1.1514	0.0020	0.0429	4.1049	12.3163	229.2661
282	126.6814	161.9669	9.0594	0.7127	0.0005	0.0020	0.0112	2.3781	3.4763	29.0316
283	260.8170	185.0391	7.6080	0.7557	0.0005	0.0020	0.0399	2.6729	5.5737	163.4544
284	207.5443	131.2495	0.2724	1.6231	0.0005	0.0020	0.0018	2.2923	4.6107	169.8367
288	1033.8372	231.5521	0.3017	2.1884	0.0327	0.0020	0.0240	3.5660	6.2918	49.7367
289	237.4737	165.4542	1.8088	6.5681	0.0309	0.0326	0.0824	2.9371	11.5732	66.8942
290	229.3210	207.9616	3.6704	3.1015	0.3359	0.0520	0.0620	3.9841	17.4567	48.4385
292	283.5333	169.9439	2.5153	8.2281	0.0005	0.0078	0.0295	2.9873	7.1586	163.1985
295	158.3894	152.6035	3.5203	2.0124	0.0005	0.0020	0.0844	3.2198	13.3192	95.5078
297	172.2863	194.7568	0.9904	3.3832	0.2232	0.0387	0.0008	4.0735	6.9368	59.6621
299	334.8101	193.7904	1.8043	3.4713	0.0017	0.0020	0.0280	3.1826	6.4036	169.8367
301	274.0511	108.5133	0.6185	1.5340	0.0005	0.0083	0.0431	3.2914	5.9768	86.7542
303	530.3843	194.2182	2.4693	0.8522	0.0005	0.0148	0.0044	3.8077	6.0536	132.8612
305	254.2017	228.8770	2.6222	1.9615	0.0005	0.0005	0.0538	3.1479	6.5793	68.4003
307	386.5644	212.7893	3.5179	1.9790	0.0005	0.0296	0.0456	3.9497	6.7666	103.7019
310	279.2344	181.9982	1.5771	5.6720	0.0005	0.0578	0.0340	4.8745	6.9062	49.4629
311	329.5324	137.5178	2.1669	0.7751	0.0005	0.0001	0.0302	3.6693	6.5584	199.8658
313	240.2434	182.2425	2.5076	7.7847	0.0005	0.0359	0.0505	4.7043	5.7791	52.4159
314	422.4864	198.1867	2.3130	3.2762	0.2929	0.0654	0.0505	2.7918	8.9948	109.8091
315	281.3281	242.6433	2.4846	3.1536	0.0005	0.0432	0.0177	5.5644	6.8840	67.1940
317	397.6414	138.8224	1.9261	6.9318	0.0005	0.1311	0.0358	3.8416	5.7203	120.9508
318	202.5846	194.2525	0.0293	7.1979	0.1478	0.0830	0.0185	3.2477	5.6239	109.1556
319	122.0659	211.3807	2.8547	0.8374	0.0005	0.0020	0.0268	4.7731	6.4854	86.9921
320	503.5917	318.2127	3.0571	5.0556	0.0005	0.0586	0.0894	5.7978	10.4189	51.7165
321	190.1440	149.8442	0.6337	2.8232	0.0005	0.0170	0.0135	4.8338	5.7109	98.2688
326	258.6025	228.8341	0.8054	1.7683	0.0005	0.0162	0.0363	3.8942	7.3405	51.9247

No.	Na	P	B	Mn	Ni	V	Co	Cr	Cu	Fe
327	93.3664	202.3120	2.2200	3.0848	0.0005	0.0244	0.0399	5.8712	5.6106	205.3021
328	131.8837	200.4259	2.9968	10.9644	0.0005	0.1225	0.0320	5.4720	11.2670	156.3502
331	346.2487	157.5254	0.2633	4.3186	0.0005	0.0264	0.0612	4.5110	5.0870	57.7116
333	151.2727	168.7988	3.0931	2.6148	0.0005	0.0052	0.0388	4.4775	6.8229	236.3209
334	200.4839	297.7339	1.0517	0.9788	0.0005	0.0020	0.0390	4.3421	12.3836	166.7279
335	355.1494	265.4834	0.3995	9.2760	0.3147	0.0506	0.0369	6.8470	6.3357	82.6415
336	433.7177	274.0409	2.2795	12.1523	0.0005	0.0619	0.0473	5.8439	10.0603	201.3317
338	1204.3922	204.5062	2.7099	14.3610	0.0005	0.1625	0.0426	5.3813	7.4161	57.5670
339	256.0354	182.2628	2.0363	3.5850	0.5795	0.0596	0.0320	5.7017	17.4840	99.2870
343	188.6857	137.4211	3.6998	5.2322	0.0005	0.0127	0.0764	4.1958	6.8281	49.1623
346	374.2970	264.3693	2.3265	9.2197	0.3861	0.0698	0.0304	4.9861	10.7167	59.7115
348	313.4589	212.0003	3.0695	1.3409	0.0005	0.0156	0.3303	3.2190	7.1535	66.1175
349	91.4563	140.5952	2.6499	2.1417	0.0005	0.0020	0.0323	3.3334	5.3435	52.7901
352	102.3863	113.3650	2.5312	2.7447	0.0005	0.0020	0.0220	3.2050	4.0122	99.7813
354	462.7392	157.0057	2.2232	2.7139	0.0005	0.0119	0.0444	2.5058	4.6388	141.1489
357	184.2122	164.6210	0.3613	2.1465	0.0005	0.0003	0.0216	5.1981	4.1585	169.5851
358	522.0521	200.0303	0.2175	3.1629	0.0005	0.0222	0.0548	4.3474	6.7986	204.2253
359	862.1913	353.3774	2.3118	20.3446	0.1761	0.1165	0.0394	5.7361	11.5661	53.1307
360	297.6425	169.9592	3.8212	1.6515	0.0005	0.0002	0.0577	4.5047	4.4807	88.3149
363	123.3911	182.0666	0.7065	1.0016	0.2722	0.0008	0.0175	7.8908	5.2753	64.4858
364	115.1331	144.9292	2.0856	0.4441	0.0005	0.0020	0.0286	3.3228	5.2180	185.4669
365	125.8806	184.5192	1.4793	6.2723	2.0836	0.1103	0.0385	5.8061	8.2127	587.6596
369	90.6322	127.6946	0.4519	0.4646	0.0005	0.0020	0.0423	2.2630	4.0949	116.0312
373	165.8387	226.5091	2.3160	3.7331	0.0005	0.0020	0.0165	2.5536	5.7475	45.0733
374	171.1768	277.9172	2.1222	0.8371	0.0005	0.0019	0.0059	5.9573	7.6949	182.3362
375	69.8656	122.4315	2.8114	0.4761	0.0005	0.0020	0.0548	3.3368	4.7792	97.0237
380	123.6871	211.0437	0.9278	4.3003	0.0358	0.0058	0.0268	3.1063	8.8590	144.5952
381	443.7583	157.9496	4.6807	2.5599	0.0005	0.0307	0.0329	4.4932	4.7945	190.9579
384	237.6001	255.5959	0.8106	2.4188	0.0005	0.0266	0.0441	4.9892	7.5412	108.0243
388	807.1641	345.5674	1.9407	3.1146	0.1128	0.0209	0.0279	4.7941	8.0624	178.3863
390	196.5050	157.3788	2.0724	5.3073	0.0005	0.0211	0.0258	4.6438	6.2595	113.8235
391	271.1498	202.2973	2.5945	2.3479	0.0005	0.0119	0.0429	4.7563	5.3387	91.6263
394	125.9283	161.3040	4.2879	0.7665	0.0005	0.0020	0.0535	3.5776	9.4295	178.3863
395	298.0418	193.5545	0.5848	7.2198	0.2911	0.1360	0.0183	3.7875	8.5166	99.9397
396	113.8457	171.7417	3.2217	0.8256	0.0005	0.0020	0.0893	3.8999	3.7917	153.8628
397	588.3423	211.4231	2.9391	9.1267	0.0100	0.0845	0.1539	5.6511	7.0537	67.2319
402	439.3346	246.6265	4.0960	11.9155	0.2774	0.2524	0.0787	3.8116	9.3515	112.9529
403	444.5586	399.7872	1.9413	9.2273	0.5525	0.0525	0.0189	8.7617	12.1586	130.0340

No.	Na	P	B	Mn	Ni	V	Co	Cr	Cu	Fe
406	177.3173	223.4433	0.5149	8.3313	0.0645	0.0525	0.0168	3.4573	7.9969	215.3538
407	1169.8365	286.6146	1.9546	14.7027	0.1426	0.1401	0.0585	4.5546	15.3598	251.2321
408	387.7579	262.9510	0.5719	2.2368	0.4461	0.0020	0.0461	3.0573	9.9366	161.4748
409	323.7340	270.7206	0.2391	16.6270	0.2685	0.3502	0.0398	5.5203	13.2343	183.2999
411	759.8630	241.2080	2.5783	11.5313	0.2047	0.0201	0.3374	4.0040	11.4955	141.7883
412	427.1028	347.2969	1.9378	101.3618	1.2051	0.8394	0.0431	6.2270	20.3776	106.2712
417	571.6562	232.3631	1.9580	3.4611	0.3380	0.0966	0.0491	4.0375	10.7752	93.5188
419	386.8009	246.9525	1.9749	9.0523	0.0988	0.1208	0.0345	4.4185	10.4448	108.0342
421	354.3876	323.9493	0.2202	12.4426	0.6482	0.2011	0.0260	5.6384	13.0750	113.4355
423	299.9734	218.8198	0.3721	3.2096	0.0185	0.0313	0.0134	4.0135	8.8920	134.5952
428	908.8005	369.5131	0.6792	13.2585	0.1250	0.0439	0.0523	5.7967	13.6819	97.3989
430	296.5979	352.6361	2.7331	6.1440	0.1330	0.0480	0.0178	5.2883	17.2505	89.9067
433	127.2435	170.4050	4.1199	3.4689	2.5923	0.0529	0.0521	4.0823	18.4106	168.6030
437	197.2683	284.0732	2.9349	2.0315	0.0217	0.0020	0.7304	4.0437	10.1640	141.8504
438	408.1028	223.6711	0.0391	5.3958	0.0836	0.0762	0.0339	3.7172	10.9073	97.3134
439	510.3885	288.5122	1.0676	2.5936	0.2175	0.0864	0.0346	4.7811	15.6926	141.6380
440	195.7475	213.1148	2.4942	5.8716	0.3366	0.1152	0.0497	4.3604	9.9390	78.3666
441	447.3463	185.9203	2.0387	1.4824	0.0005	0.0020	0.0416	4.1977	5.0985	94.4773
443	151.4942	197.4786	2.0712	7.5660	0.3822	0.1260	0.0317	4.0714	8.3324	105.5999
444	149.0552	223.2448	0.1516	3.0673	0.0005	0.0168	0.0402	5.2297	8.7112	91.1972
445	740.9056	218.2382	0.1376	20.9210	0.8944	0.1997	0.0515	4.6382	12.1848	107.8341
446	759.5296	222.4219	2.8154	2.9884	0.0005	0.0489	0.0048	3.9512	10.3825	87.2335
449	212.9170	342.9462	1.1665	7.1449	0.6866	0.1033	0.0265	5.8957	17.7095	110.8441
453	982.1097	260.0306	2.2396	11.8461	0.6104	0.1068	0.0276	4.4947	11.4892	77.3905
457	114.7859	132.4403	0.3381	5.1001	0.0005	0.0181	0.0392	3.9819	5.0284	114.0915
460	177.6557	223.2813	0.5738	6.8191	1.4308	0.1246	0.1081	3.8075	7.5222	187.0039
461	476.7477	208.1197	2.7146	2.2946	0.0359	0.0383	0.0279	2.9067	6.0806	172.8705
463	83.1356	181.4019	3.8424	3.1189	0.3845	0.0402	0.0343	4.6119	13.2767	102.1152
465	292.0130	241.1619	1.7645	2.5275	1.0827	0.0230	0.0431	3.3376	7.6404	86.4617
472	271.7787	226.1560	0.8099	4.0432	0.4076	0.0105	0.0380	3.9838	7.9414	96.5907
473	1109.5499	179.4996	4.5175	11.4868	0.0595	0.0110	0.0360	5.1409	5.0153	124.5619
475	234.4572	229.0436	0.4428	0.6666	0.0588	0.0020	0.0035	2.9743	6.5874	36.4837
476	99.5971	241.1478	0.0805	3.6377	0.0079	0.0020	0.0235	4.9077	23.2682	140.0743
477	78.3302	257.5873	1.8264	2.0784	0.1113	0.0080	0.0402	3.4234	10.3238	134.1175
479	107.2983	140.8643	0.1325	5.4659	0.0005	0.0234	0.0334	3.9994	5.2537	142.2811
480	61.8919	125.5807	2.5448	0.5324	0.0005	0.0020	0.0356	2.9505	5.5110	121.5164
481	797.3149	381.1914	1.6497	21.5086	1.0911	0.1222	0.0207	8.0878	21.1602	133.0482
482	342.1308	280.7119	2.6871	5.5169	0.2313	0.1178	0.0192	4.2435	10.9535	109.9234

No.	Na	P	B	Mn	Ni	V	Co	Cr	Cu	Fe
484	1061.3301	268.8802	2.3717	15.8752	0.1026	0.3203	0.0211	8.4206	11.2924	129.0376
489	109.7159	242.3937	2.9108	0.9498	0.0005	0.0020	0.0473	2.2025	5.9522	72.4409
490	777.9909	282.1745	1.1262	35.4180	0.0998	0.1368	0.0248	4.5110	9.6507	85.1927
498	129.0943	261.2113	1.3582	5.0194	1.5937	0.0747	0.0072	3.7109	9.1627	109.1561
499	389.1894	288.2068	0.1484	1.3384	0.0005	0.0194	0.0216	5.1249	9.2794	42.4559
500	265.9726	169.7835	1.8979	33.7972	0.2419	0.2380	0.0180	3.3668	11.8385	48.8580
503	91.1035	136.7805	2.4712	2.2801	0.0005	0.0020	0.0373	2.4186	3.9797	203.6267
504	161.2641	178.9646	0.3675	3.7441	0.7929	0.0006	0.0175	2.7696	21.8222	57.7064
514	105.6188	195.6285	2.4539	0.8967	0.1579	0.0020	0.0412	4.0118	5.9831	59.6181
515	292.8938	337.7953	1.1399	2.1236	0.0005	0.0515	0.0146	5.5481	7.5478	62.6597
516	188.5316	153.6524	1.9051	4.4148	0.0005	0.0020	0.0366	2.4113	4.9357	67.8059
519	188.0141	174.1968	0.0133	4.3572	0.0358	0.0356	0.0152	3.1195	7.5476	77.8257
520	175.1989	232.5493	2.6693	4.0745	0.7667	0.0597	0.0278	7.8714	7.1506	79.2902
521	258.7398	217.3096	2.9275	2.2176	0.0286	0.0121	0.0139	4.7996	10.3123	123.7908
522	123.2255	207.1938	2.4099	2.1189	0.4033	0.0391	0.0310	3.9653	14.0134	128.5847
523	151.7137	185.8372	2.1521	1.0035	0.1330	0.0020	0.0300	3.8646	7.0165	87.8360
524	140.9840	168.0598	2.3021	2.7814	0.0005	0.0020	0.0359	3.0684	7.1464	82.7728
525	380.4433	228.4301	2.6666	10.2639	0.2849	0.2222	0.0280	7.5871	9.8009	84.4703
528	175.0987	149.0996	0.8647	2.0003	0.0005	0.0072	0.0199	2.9146	4.5122	85.9743
529	402.1344	213.2624	0.6509	4.4687	0.4184	0.0993	0.0219	5.2903	12.8769	58.2531
530	151.6168	214.1671	1.6811	7.3000	0.0005	0.3109	0.0167	4.2907	7.6400	93.9648
531	443.7815	202.2898	1.6372	4.1266	0.3055	0.0081	0.0073	4.9498	11.8554	94.5614
532	352.9050	199.6504	1.7872	1.2037	0.0447	0.0020	0.0589	3.4635	8.3084	99.9485
533	228.4033	282.8877	2.4777	1.6744	0.1620	0.0020	0.0570	5.3163	11.7354	100.3072
534	495.6183	136.4292	2.5850	4.1536	2.2429	0.0325	0.0243	3.0683	5.2075	111.5965
538	152.9789	145.5282	2.2484	1.3060	0.0005	0.0091	0.0409	2.5263	4.8037	89.1492
539	368.3673	224.2568	2.1507	3.2310	0.3084	0.0020	0.0393	4.1863	9.3663	89.9585
541	272.9143	225.0608	0.1933	3.8198	0.0005	0.0694	0.0019	4.4521	8.7273	36.2594
542	172.3767	143.1101	3.8079	4.7926	0.0005	0.1388	0.0353	2.6725	7.6277	55.8794
543	110.3788	228.0310	0.2562	2.8061	0.0005	0.0239	0.0324	3.3427	6.3571	121.8738
545	127.6537	217.5304	2.9467	1.4429	0.0180	0.0020	0.0361	3.6127	6.5246	111.5712
546	262.0461	217.4686	1.1300	2.4164	0.0005	0.0012	0.0189	3.5817	8.9932	123.1411
547	115.8466	113.9054	1.8058	1.9858	0.0005	0.0020	0.0275	2.2399	6.2430	129.0675
548	154.7999	119.3869	2.3223	4.5961	10.8345	0.0020	0.0239	2.2092	5.5703	107.6705
549	104.5390	175.9133	2.0715	2.4039	0.0005	0.0090	0.0228	2.7460	5.2660	124.4387
551	174.8337	203.3989	0.1235	2.4978	0.0804	0.0020	0.0207	3.5575	8.7557	129.3143
552	172.4772	193.2082	2.0963	0.6451	0.0005	0.0028	0.0464	3.0442	6.8882	137.5524
553	144.9776	172.2464	1.7093	1.0399	0.0627	0.0020	0.0266	3.2274	7.8778	139.1773

No.	Na	P	B	Mn	Ni	V	Co	Cr	Cu	Fe
554	253.9381	210.5239	0.6429	1.1212	0.0005	0.0020	0.0056	3.3290	6.5540	140.5074
556	204.9782	180.6053	2.3898	2.3919	0.0005	0.0020	0.0150	3.8608	5.7096	153.3921
558	132.2497	149.5909	2.4297	0.9420	0.0005	0.0020	0.0346	4.0035	5.4382	50.0201
559	77.3764	92.2296	2.3320	0.3569	0.0005	0.0020	0.0130	1.6142	2.0208	179.9324
560	125.1848	188.5656	2.0976	0.9351	0.0409	0.0020	0.0380	2.9660	5.9208	182.3495
561	324.6650	201.1197	2.7870	7.0838	0.1945	0.0648	0.0372	3.7274	8.5311	190.5074
565	252.5871	209.7481	1.4133	4.8996	0.2739	0.0020	0.0258	3.5422	9.3134	193.1641
702	13.3107	186.7554	0.6753	0.2688	0.0005	0.0020	0.0010	0.7019	2.0172	82.7062
703	218.6842	251.0640	0.1318	16.0107	3.2214	0.1245	0.1234	4.2850	14.7909	54.2717
704	223.2870	242.2804	0.9546	3.8947	0.4435	0.1581	0.1691	4.0386	14.5160	48.8966
705	170.7992	163.5878	2.0300	2.9285	0.2582	0.0275	0.0381	3.0909	9.4579	26.5129
706	288.6409	241.7676	2.0712	10.6013	0.3975	0.4546	0.1185	3.1068	8.4204	171.4177
709	114.7122	221.6664	3.5954	11.2879	0.4422	0.0811	0.0883	4.1101	13.6356	43.3107
710	270.3774	250.2991	1.4173	7.9726	0.6073	0.0439	0.0524	3.0905	8.8185	30.8810
711	156.1621	234.3732	1.7151	2.7580	0.1851	0.0343	0.0310	3.1438	9.6524	24.8798
712	169.4863	282.8848	0.6055	11.8767	0.9319	0.1106	0.1794	8.0517	12.7923	202.6221
713	299.2800	214.4890	2.2626	2.0680	0.3532	0.0454	0.0261	3.5863	10.7189	32.4698
722	396.9563	386.6063	0.5166	1.8355	0.0005	0.0222	0.0010	6.9335	8.5420	53.4911
723	261.0149	251.9841	2.4324	9.7657	0.5781	0.0829	0.0810	5.3985	17.5627	40.7906
724	153.9308	219.5005	1.5068	3.1065	0.6755	0.0381	0.0535	3.9944	11.2173	38.1146
725	514.3538	215.5406	2.6984	8.4422	0.3855	0.0671	0.0460	4.2320	11.6195	34.6209
726	137.5120	144.9078	1.3949	8.8536	0.2880	0.0958	0.0741	3.4547	7.6205	63.4236
727	197.4125	278.7917	0.2770	3.8796	0.2654	0.1543	0.0165	4.4991	16.5680	33.1344
728	136.4674	224.7813	1.5425	6.3324	0.8305	0.0701	0.0884	3.7944	7.3421	32.2324
729	240.1326	269.8450	2.2656	4.2332	0.1964	0.0543	0.0514	4.2300	9.0071	44.0686
730	401.0945	205.8882	2.2683	10.5704	0.5661	0.1920	0.1000	4.4709	15.3211	55.7274
731	172.3555	239.7749	1.7484	2.8628	0.2987	0.0461	0.0295	4.9391	9.7205	80.4068
734	329.7457	160.0238	2.3876	29.7712	0.3954	0.1097	0.1173	3.9159	6.1311	96.6302
735	344.1740	254.6221	1.1037	19.3608	0.6297	0.1257	0.0927	3.9571	13.0109	42.8935
739	486.2775	363.2931	2.1463	6.1466	1.0063	0.0687	0.1052	4.6617	15.9153	91.2476
740	317.3728	233.0339	0.4287	5.0548	0.2119	0.0420	0.0616	3.2588	8.1724	33.7279
742	212.7566	231.1962	2.3952	2.7074	0.2320	0.0417	0.0400	3.8487	7.2584	90.2671
744	236.2119	187.0965	2.1284	1.3760	0.6206	0.0304	0.0849	3.5713	5.1506	29.7768
745	219.1002	205.5365	0.9967	2.5372	0.2938	0.0420	0.0396	3.4259	8.5514	44.7867
746	225.7316	197.2899	0.4916	6.8709	0.4214	0.0789	0.0612	3.8495	10.1466	47.5751
747	212.9439	235.2055	0.3542	3.4859	0.5392	0.0228	0.0086	4.0240	14.9951	35.5993
748	779.9039	293.0639	1.0379	6.1739	0.9173	0.1100	0.0821	5.5077	16.4521	94.6558
755	176.9771	245.6812	2.5124	8.3488	0.6376	0.0849	0.0478	3.8217	14.3095	43.2823

No.	Na	P	B	Mn	Ni	V	Co	Cr	Cu	Fe
760	389.0420	251.6087	1.3480	11.2260	0.3342	0.0789	0.0856	4.0817	10.5922	46.6416
761	452.1088	148.3767	1.8775	6.5695	0.5113	0.0415	0.0783	3.6688	8.3813	36.2066
762	377.5574	227.9234	1.2391	5.9948	0.8444	0.0387	0.1073	4.1848	9.9857	95.7062
766	155.9061	225.7152	1.7809	5.0952	0.5326	0.0500	0.0656	3.3965	3.2769	31.4321
767	141.2653	287.7614	0.1112	6.9212	1.1911	0.1614	0.3747	6.1933	12.8654	48.2401
768	221.5115	204.1147	3.4413	10.6964	6.3336	0.0454	0.0741	4.7185	8.1225	56.4811
769	144.6872	225.2723	2.7989	2.4416	0.1543	0.0468	0.0215	4.0680	10.3410	41.1200
775	60.6501	295.4683	0.5606	0.1358	0.0005	0.0020	0.0010	0.0509	3.0509	11.1947
777	137.7800	243.0604	1.9317	1.8612	0.2200	0.0348	0.0166	3.2060	7.8164	40.6072
780	571.1633	240.7662	0.1433	19.2306	0.4674	0.0446	0.0010	4.7011	8.7371	48.6424
781	333.8766	196.3421	0.8938	15.0580	0.5200	0.3337	0.1160	4.1413	14.5978	36.1883
782	196.2776	257.3746	1.8153	10.9958	5.5113	0.1443	0.0833	3.7615	11.2399	42.6450
785	122.5956	242.6096	1.3340	4.0196	0.4142	0.1650	0.0681	4.1311	13.2606	33.7046
787	604.4536	197.2860	0.5861	20.9700	0.4918	0.1068	0.1008	3.4682	9.7547	33.6293
788	181.8529	177.9132	0.6634	9.8489	0.2664	0.0402	0.0640	2.9522	8.6897	70.5709
789	496.3216	252.9062	1.1806	11.5143	1.7005	0.0608	0.1185	10.2133	13.3048	64.1139
790	218.8997	187.5384	1.2065	12.7732	4.1050	0.0754	0.1052	3.6754	8.9749	99.5297
791	229.4563	249.8833	2.3153	1.9525	0.2327	0.0295	0.0186	3.5660	8.4285	29.7870
794	123.7973	147.7447	1.8552	4.7091	0.5098	0.0257	0.0676	3.2337	9.3761	62.0606
795	1475.2497	198.0514	1.1305	9.9700	0.5163	0.0464	0.0518	3.6836	8.8698	38.3433
799	354.6053	184.0115	2.3061	1.7110	0.3070	0.0350	0.0607	3.7241	11.8540	26.0254
800	310.6603	212.4262	0.3970	1.6605	0.1657	0.0479	0.0256	3.5491	9.3350	32.4246
801	192.3203	276.0708	1.0740	5.2379	0.6556	0.1024	0.0796	4.6056	11.6690	49.5550

No.	Mo	Se	Sr	Zn	Age	Gender	Smoking statu	Drinking Statu	Arsenism
3	0.0076	0.6095	5.3504	85.8874	63	1	1	1	1
6	0.0076	0.4846	9.3368	102.8313	57	2	0	0	1
7	0.4392	0.5275	5.8945	109.2798	57	1	1	0	1
8	0.1105	0.5837	7.5120	129.1136	65	1	1	2	1
10	0.2198	0.3219	6.2418	153.0499	43	1	1	1	1
13	0.0939	1.0867	23.4567	140.8271	55	1	1	0	1
15	0.0863	1.2416	14.7206	128.3914	26	2	0	0	0
16	0.1478	1.1391	10.8339	111.2557	32	2	0	0	0
17	0.0729	0.6938	16.1092	179.0901	47	2	0	0	1
20	0.0144	0.4945	1.3665	87.7461	64	1	2	2	1
21	0.2371	0.5933	7.0842	71.0515	50	1	1	0	1
22	0.0076	0.9117	4.9822	70.2755	49	1	0	1	0
23	0.1090	0.4497	5.5818	174.6779	50	1	1	2	1
24	0.1050	0.8675	8.0524	163.2076	40	1	0	0	1
26	0.0736	0.3765	4.8773	124.7214	61	1	0	0	1
27	0.5254	0.6990	26.9336	116.2723	54	1	1	1	1
28	0.0795	0.1610	8.4332	118.4015	55	2	0	0	1
29	0.1194	0.6488	6.2749	88.5958	45	1	0	0	1
31	0.5260	0.4550	26.6321	115.3305	28	1	0	0	1
32	0.1275	0.5289	10.2961	106.2464	46	2	0	0	1
34	0.1295	0.1014	5.8929	169.6715	48	1	1	2	1
37	0.1391	0.3748	5.0832	74.2583	47	1	1	1	1
39	0.0049	0.4557	9.2404	104.7571	68	1	0	0	1
40	0.0423	0.8698	16.7098	113.9515	38	2	0	0	0
41	0.2193	0.1410	11.9214	349.0794	49	2	0	0	0
42	16.9749	0.3972	14.6271	168.6144	54	2	0	0	1
44	0.0185	0.4027	10.1522	83.9857	45	1	1	0	1
46	0.1289	0.4964	5.1810	118.1097	63	1	2	1	1
47	0.0751	0.4401	5.2546	76.2040	58	1	2	1	1
49	0.2971	0.3520	14.9690	738.9666	44	2	0	0	1
50	0.2003	0.1381	7.9790	74.3482	65	2	0	0	1
51	0.0506	0.7604	4.7360	98.6426	56	1	1	1	0
53	0.0076	0.3715	15.6783	190.3547	44	2	0	0	1
54	0.4331	0.4173	13.0522	106.1916	38	1	0	0	1
56	0.0901	0.2641	16.0507	177.0016	57	2	0	0	1
57	0.2302	0.5025	9.0348	67.0681	59	2	0	0	1
59	0.0342	0.2643	7.4495	117.6134	60	2	0	0	1
62	0.0934	0.4371	5.1339	82.6027	50	1	1	2	1

No.	Mo	Se	Sr	Zn	Age	Gender	Smoking statu	Drinking Statu	Arsenism
64	0.0292	0.4479	4.6526	128.2275	41	1	0	0	1
66	0.1934	0.3490	20.4452	630.4172	53	2	0	0	1
67	0.0014	0.8256	4.8437	157.4892	60	1	2	2	1
68	0.0120	0.4139	6.4709	96.5838	35	2	0	0	1
69	0.2383	0.0271	14.5072	130.4708	32	2	0	0	1
71	0.0076	0.7341	6.3117	127.5926	44	1	1	1	1
72	0.1652	0.5038	21.1845	79.8394	57	2	0	0	1
73	0.2478	0.4959	5.3745	62.3989	64	2	0	0	1
74	0.2059	0.3895	4.8998	107.5764	52	1	1	2	1
75	0.2003	0.3186	7.4447	106.6842	52	2	0	0	1
76	0.0076	0.5432	6.5782	70.9981	41	2	0	0	1
78	0.0769	0.5575	6.3198	103.9404	50	2	0	0	1
79	0.1233	0.1645	19.3023	133.3770	46	2	0	0	1
81	0.1929	0.4619	4.4568	66.7776	59	2	0	0	1
82	0.0374	0.4138	8.8179	98.2157	43	2	0	0	1
83	0.1180	0.6803	16.3908	356.1738	54	2	0	0	1
84	0.1388	0.4921	4.0993	90.9275	60	1	2	2	1
85	0.1752	0.6083	8.6413	89.4800	46	1	1	0	1
86	0.2654	0.1227	15.2957	168.0169	42	2	0	0	1
89	0.0825	0.3031	20.2540	191.6764	64	2	0	0	1
90	0.0355	0.5040	10.4863	178.3747	44	2	0	0	1
92	0.0052	0.5270	7.5171	77.9978	65	2	0	0	1
93	0.1430	0.2966	5.3800	105.3271	42	2	0	0	0
94	0.0498	0.4526	10.7215	258.1981	51	2	0	0	1
95	0.2576	0.4327	8.9528	113.8042	2	1	0	0	1
96	0.2141	0.2623	33.5282	139.2686	49	1	2	2	1
97	0.0418	0.6884	4.5360	123.6808	40	1	0	0	1
98	0.1232	0.4341	10.8772	112.2833	59	2	0	0	1
100	0.3048	0.4693	12.5413	244.6304	44	2	0	0	1
102	0.0076	0.1344	3.8326	38.1098	63	1	2	2	1
103	0.1658	0.3168	17.4603	141.2348	56	2	0	0	1
105	0.0943	0.1262	10.4835	62.5236	38	2	1	0	1
107	0.1771	0.4431	15.2320	234.3059	49	2	0	0	1
109	0.0961	0.4915	8.3266	74.9351	56	2	0	0	1
111	1.8376	0.1086	10.2855	41.5739	64	1	0	2	1
119	10.4342	0.4129	6.1554	65.3098	51	2	0	0	1
124	0.0792	0.2309	6.4623	106.2206	59	2	0	0	1
126	0.2555	0.4617	24.1438	102.2551	57	1	2	1	1

No.	Mo	Se	Sr	Zn	Age	Gender	Smoking statu	Drinking Statu	Arsenism
127	0.0830	0.2394	4.0958	49.4756	67	1	2	0	1
128	0.0076	0.3647	0.9730	70.0368	71	2	0	0	1
129	0.2642	1.0266	30.8521	208.8125	47	2	0	0	1
130	0.0135	1.1360	8.4093	143.6901	47	1	1	2	1
131	0.1195	0.6281	8.3584	107.0229	49	1	1	1	1
132	0.0116	0.3009	10.6079	143.3137	45	1	2	2	1
136	3.1361	0.2906	9.1146	50.0805	40	1	2	1	1
138	0.0076	0.2620	4.3712	45.4084	36	1	0	0	1
139	0.0359	0.6724	8.1803	80.1287	51	2	0	0	0
140	0.1768	0.2348	8.9297	106.6370	42	1	2	2	1
145	0.0265	0.5229	4.8194	81.9337	65	1	1	1	1
148	0.1100	0.4215	1.7443	86.8985	61	1	0	0	1
150	0.2121	0.3378	10.7037	197.9601	62	2	0	0	1
152	0.2234	0.3041	7.1168	119.4868	51	1	1	1	1
155	0.0219	0.6753	7.6869	119.8616	52	1	1	1	1
158	0.1729	0.6865	11.6387	395.7756	40	2	0	0	1
159	0.0666	0.5465	15.9030	146.4945	49	1	1	1	1
163	0.2832	0.2199	11.2933	98.4767	70	2	0	0	1
165	0.2200	0.2575	6.3652	72.4747	53	1	1	1	1
167	0.4687	0.6541	13.1225	162.0070	43	1	1	2	1
169	0.2008	0.5102	21.0806	202.0238	62	2	0	0	1
170	0.3856	0.5037	1.0328	95.9272	52	1	1	0	1
173	0.1148	0.2386	19.1169	209.4366	52	2	0	0	1
174	0.0395	0.2594	5.1469	80.2933	60	2	0	0	1
175	23.0649	0.1023	7.3997	50.1999	50	1	2	0	1
177	0.2181	0.2999	8.8283	112.9399	65	2	0	0	0
178	0.2002	0.3911	4.7163	95.1141	44	1	1	0	1
183	0.1581	0.4974	5.9249	78.5497	68	2	0	0	1
187	0.1951	0.7394	35.8727	222.1726	30	2	0	0	0
189	0.1448	0.4040	6.6696	106.6886	50	1	1	2	1
191	0.2194	0.1008	6.8881	85.4304	70	2	0	0	1
192	0.0938	0.3083	8.0622	154.5471	73	2	0	0	1
194	0.2223	0.5428	10.5999	72.6741	46	2	0	0	1
196	0.1018	0.5800	9.7297	166.0038	46	2	0	0	1
199	0.0791	0.4675	6.1411	106.9729	42	1	1	1	1
200	0.1482	0.7217	39.6237	159.8009	60	2	0	0	1
201	0.0686	0.5794	5.2231	61.6264	48	2	0	0	1
203	0.0251	0.2726	1.3274	80.4461	56	1	1	0	1

No.	Mo	Se	Sr	Zn	Age	Gender	moking statu	rinking Statu	Arsenism
204	0.0231	0.3159	3.0417	148.0457	50	2	0	0	1
205	0.0076	0.2271	1.4244	79.1777	50	1	1	0	1
206	0.1276	0.2079	5.9084	103.3856	53	2	0	0	1
209	0.0187	0.5274	6.0222	63.7996	42	1	1	0	1
211	0.2574	0.6254	7.3025	70.5228	45	1	1	1	1
213	0.0046	0.3828	0.8443	55.3395	53	2	0	0	1
214	0.0036	0.7152	5.1043	80.2583	30	1	2	2	0
215	0.0558	0.2265	1.5251	62.4492	50	2	0	0	1
217	0.2099	0.6536	3.6520	98.6183	60	2	0	0	1
219	0.0076	0.4321	3.2581	227.3266	35	2	0	0	1
220	0.0076	0.0100	0.2000	21.7854	50	2	0	0	1
221	0.0537	0.6477	4.3471	127.3330	65	1	0	0	1
224	0.0100	0.1808	1.4271	73.6943	72	1	0	0	1
226	0.0557	0.4201	1.0953	130.4762	43	1	1	0	1
227	0.0367	0.6685	12.0337	97.3277	26	2	0	0	0
229	0.0076	0.5238	3.7238	196.8622	51	1	0	0	1
231	0.0076	0.4336	1.6448	74.4544	29	1	1	0	1
232	0.0076	0.2057	1.0783	56.8961	49	1	1	2	1
233	0.0544	0.4985	3.2697	78.6163	46	1	1	0	1
234	0.0637	0.3610	4.4553	54.8674	65	2	0	0	1
235	0.0289	0.3117	1.3790	104.1009	53	1	0	1	1
236	0.0187	0.6716	10.1190	199.5560	52	2	0	0	1
238	0.0164	0.1914	9.2407	140.5786	63	2	0	0	1
239	0.0076	0.1883	5.3797	51.7153	46	2	0	2	1
240	0.3731	0.5215	1.8234	106.3775	37	1	0	0	1
241	0.0076	0.5875	2.1249	90.2725	34	1	0	1	1
242	0.0076	0.2350	2.0852	72.6983	46	1	0	0	1
243	0.1500	0.5665	10.4479	95.0530	58	2	0	0	1
244	0.1006	0.4702	5.5172	83.4406	68	2	0	0	1
245	0.1314	0.1827	2.5967	35.9750	49	2	0	0	1
246	0.0076	0.1478	1.6006	71.2196	59	1	1	2	1
248	0.0393	0.6643	13.5359	114.1465	42	2	0	0	0
251	0.1795	0.3131	0.7487	60.7242	74	1	1	2	1
254	0.0076	0.2962	5.6780	91.4897	76	2	0	0	1
255	0.0493	0.6608	0.8197	50.7131	60	2	0	0	0
256	3.3122	0.4252	1.9553	60.6962	68	1	1	1	1
257	0.0980	0.6501	8.6928	151.1959	45	2	0	0	0
259	0.0076	0.6245	6.2088	80.5845	47	2	0	0	0

No.	Mo	Se	Sr	Zn	Age	Gender	Smoking statu	Drinking Statu	Arsenism
260	0.1719	0.5790	10.4498	329.0044	48	2	0	0	1
261	0.0768	0.5281	6.7366	83.1267	62	2	0	0	1
264	0.0675	0.6205	7.4317	80.9129	58	2	2	0	0
265	0.0076	0.5264	5.2919	88.9347	31	2	0	0	1
269	0.0076	0.6146	14.0327	63.3980	64	1	1	1	0
270	0.0076	0.5067	2.0915	78.9466	65	1	1	1	1
272	0.0076	0.6119	16.2918	176.8538	51	2	0	1	0
273	0.0204	0.6080	1.0103	76.3711	37	1	1	1	0
275	0.0341	0.5158	5.6109	63.6238	54	2	0	0	1
276	0.1670	0.6051	13.5267	77.5534	45	2	0	1	0
277	0.0076	0.5729	16.1694	93.9615	41	2	0	0	0
279	0.0701	0.4988	6.2569	181.8314	50	2	0	0	1
281	0.0147	0.3714	6.7051	125.4197	59	2	0	0	1
282	543.4646	0.5694	1.4121	73.7628	41	1	1	1	0
283	0.0144	0.3171	0.8885	81.7908	52	1	1	0	1
284	0.0286	0.2211	3.3522	57.2992	50	2	0	0	1
288	0.0484	0.4553	5.8801	98.7105	43	1	1	0	1
289	0.1182	0.5666	16.7285	148.8082	38	2	0	0	0
290	0.0607	0.5769	11.3098	106.6775	45	2	0	0	1
292	0.0227	0.5655	3.7341	105.8217	56	2	0	0	0
295	0.0247	0.5597	14.8915	133.8950	43	2	0	0	0
297	0.0933	0.6240	5.1723	80.0838	62	1	1	1	1
299	0.0242	0.4181	2.0233	90.3765	68	1	1	1	1
301	0.3318	0.5543	13.1705	78.9805	37	2	0	0	0
303	0.0819	0.2789	5.3114	91.4090	66	1	2	0	1
305	0.0076	0.5409	3.2014	70.0528	42	1	1	1	1
307	0.1323	0.6150	4.2957	76.5981	62	1	2	0	1
310	0.1994	0.5493	6.4314	61.4354	50	2	0	0	0
311	0.0076	0.1111	1.4681	55.0796	54	2	0	0	1
313	0.0076	0.5425	3.3973	98.1034	41	2	0	0	0
314	0.1003	0.3346	6.4795	87.8297	44	1	1	2	1
315	0.0769	0.6247	2.9632	100.9596	56	1	0	0	1
317	0.3794	0.3009	8.2520	63.0268	60	2	0	0	1
318	0.0258	0.6184	2.0889	48.6661	64	1	1	2	1
319	0.0365	0.4779	1.4933	96.1632	41	1	1	2	1
320	0.0466	0.5194	2.6013	125.8792	65	1	1	1	0
321	0.0076	0.4863	13.0552	71.5388	43	2	0	0	0
326	0.1930	0.4742	5.6774	87.8168	60	1	1	1	0

No.	Mo	Se	Sr	Zn	Age	Gender	moking statu	rinking Statu	Arsenism
327	0.0076	0.6054	4.9648	190.8980	42	2	0	0	1
328	0.0040	0.4672	9.6068	128.7506	35	2	0	0	0
331	0.0090	0.5371	3.4491	99.2914	46	2	0	0	1
333	0.0076	0.5176	1.7793	59.0854	58	2	0	1	1
334	0.0699	0.4651	2.8074	221.4044	64	1	1	0	1
335	0.0627	0.4437	2.4325	95.3464	62	1	1	2	1
336	0.1064	0.4205	4.2768	159.1610	60	2	0	0	1
338	0.0262	0.4005	12.0206	99.6879	62	2	0	0	1
339	0.1234	0.4647	3.4114	80.4701	53	2	0	0	0
343	0.0076	0.4501	5.9507	66.8001	36	2	0	0	0
346	0.2938	0.4403	11.3105	204.2494	36	2	0	0	0
348	0.0639	0.3946	5.8494	103.9993	45	2	0	1	1
349	0.0076	0.4313	6.1278	102.7316	35	2	0	0	0
352	0.0618	0.4196	6.9541	91.3559	42	2	0	0	0
354	0.1379	0.3801	4.8240	58.0809	57	1	2	0	1
357	0.0076	0.3133	1.0640	58.5248	62	1	0	0	1
358	0.0484	0.3083	1.1118	104.7008	47	1	1	0	1
359	0.1845	0.2623	13.8619	148.5661	55	1	1	1	1
360	0.0804	0.2561	3.2556	74.1231	54	1	1	0	1
363	0.0471	0.2327	1.4658	83.6534	37	1	1	0	1
364	0.0076	0.2320	1.2073	74.5089	43	1	1	2	1
365	0.2775	0.1538	4.4000	92.2273	45	1	1	0	1
369	0.0552	0.4105	0.7431	58.7908	68	1	1	2	0
373	0.0484	0.1288	3.4690	89.9733	62	1	1	0	1
374	0.0076	0.1048	0.8592	120.0256	45	1	1	0	1
375	0.0082	0.6456	1.0453	82.8171	39	1	1	1	1
380	0.0272	0.2428	2.1846	83.2895	45	1	1	1	1
381	0.0460	0.2835	2.2741	84.9356	60	1	1	0	1
384	0.1028	0.3430	1.7283	102.2889	44	1	1	1	1
388	1.3022	0.3941	13.2064	153.2825	52	1	1	1	1
390	0.0434	0.2146	2.4684	61.0626	56	2	0	1	1
391	0.0076	0.5658	1.9114	100.7302	59	1	1	0	1
394	0.0076	0.2859	1.0939	107.6050	53	2	0	1	1
395	0.2182	0.3951	12.9347	63.2128	39	2	0	0	0
396	0.0367	0.1579	1.0368	77.6872	55	1	1	0	1
397	0.1291	0.5554	19.4117	111.4418	32	1	1	1	1
402	0.2217	0.3823	10.0775	68.0299	52	1	2	2	0
403	0.1525	0.1836	2.4636	192.2434	61	1	2	0	1

No.	Mo	Se	Sr	Zn	Age	Gender	Smoking statu	Drinking Statu	Arsenism
406	0.0564	0.2720	3.9697	104.7290	51	1	1	0	1
407	0.1551	0.9629	8.8573	178.4473	56	1	1	1	1
408	0.0084	0.3738	2.9688	137.5785	33	1	1	0	0
409	0.1531	0.3734	24.4366	95.9930	43	1	1	0	1
411	0.0381	0.3320	22.1001	106.7105	38	2	0	0	1
412	0.5093	0.3182	32.2563	197.2298	47	2	0	0	1
417	0.1851	0.3668	3.1642	112.4858	53	1	1	2	0
419	0.0155	0.3035	7.3474	109.8221	45	1	1	1	1
421	0.1383	0.2837	3.2636	162.5526	40	1	0	0	1
423	0.0957	0.2787	2.1136	97.8052	53	1	2	1	1
428	0.1471	0.2647	12.9792	140.8901	55	2	0	0	1
430	0.0701	0.3649	19.9615	181.4436	47	2	0	0	0
433	0.1045	0.3530	12.2595	348.4650	57	2	0	0	0
437	0.1059	0.3451	1.1172	121.7566	49	2	0	0	0
438	0.0980	0.2637	3.1016	94.1058	51	1	2	0	1
439	0.1711	0.2198	1.8387	111.7649	46	1	2	0	1
440	0.2943	0.2923	8.0717	117.6647	40	2	0	0	0
441	0.0076	0.2177	1.0086	75.6907	64	1	0	0	1
443	0.1495	0.2721	7.4838	86.4832	35	2	0	0	0
444	0.0147	0.2687	6.5317	97.1180	39	2	0	0	0
445	0.1142	0.2433	7.6230	105.1025	45	2	0	0	0
446	0.2858	0.2119	8.3946	117.6757	53	2	0	0	1
449	0.1699	0.2115	7.3096	162.7993	62	2	0	0	1
453	0.1160	0.2042	32.2482	131.7747	51	2	0	0	1
457	0.0231	0.1478	9.2686	52.8976	57	2	0	0	1
460	0.2198	0.1198	13.7008	268.3493	59	2	0	0	1
461	0.0132	0.1074	3.8528	89.9956	50	1	1	1	1
463	0.0455	0.1047	11.2268	54.4180	46	2	0	0	1
465	0.0426	0.1045	2.2296	112.2517	59	2	0	0	1
472	0.1444	0.1023	20.6112	138.5647	45	2	0	0	1
473	0.0577	0.3550	2.6459	92.0257	51	2	0	0	1
475	0.0614	0.2416	0.7659	95.4986	46	2	0	0	0
476	0.0141	0.2301	2.1394	154.6621	46	2	0	1	0
477	0.1120	0.3814	3.5929	141.6633	46	1	1	0	1
479	0.0076	0.3965	3.6160	87.5911	45	2	0	0	1
480	0.0076	0.3972	0.9019	79.6516	40	1	1	0	1
481	0.0987	0.2232	20.2070	271.0389	40	2	0	0	0
482	0.1740	0.4046	2.8470	97.1235	47	1	1	1	1

No.	Mo	Se	Sr	Zn	Age	Gender	Smoking statu	Drinking Statu	Arsenism
484	0.0527	0.4112	4.2101	74.3265	74	2	0	0	1
489	0.0076	0.2186	1.4607	111.5157	49	1	1	0	0
490	0.1176	0.4246	9.4636	133.2587	62	2	0	0	1
498	0.2807	0.4435	9.6781	107.0454	46	2	0	0	1
499	0.0076	0.5220	1.0938	121.9633	45	1	1	0	1
500	0.1508	0.6462	16.4015	71.6379	59	1	1	1	1
503	2.4934	0.2186	2.8529	60.1142	42	2	0	0	0
504	0.0840	0.5199	3.0532	53.7994	50	1	1	0	1
514	0.0361	0.3382	1.4922	94.8109	46	1	0	1	1
515	0.0312	0.1175	1.4669	117.1385	45	1	1	1	1
516	0.1192	0.2986	1.3540	68.4497	49	1	1	2	1
519	0.0076	0.2761	3.5709	74.7243	47	1	0	2	1
520	0.0628	0.1123	1.8297	125.3059	51	1	0	0	1
521	0.0313	0.2102	3.1602	109.5244	47	1	1	0	0
522	0.2388	0.1958	3.2578	182.3202	53	1	1	2	0
523	0.0076	0.1891	6.6495	124.5354	52	2	0	0	0
524	0.0153	0.1968	11.8107	67.5981	45	2	0	0	1
525	0.1144	0.1678	5.6263	82.0301	35	2	0	0	1
528	0.0588	0.3701	4.3748	63.9502	57	2	0	0	1
529	0.0065	0.1887	5.6350	133.2074	61	2	0	0	0
530	0.0690	0.4137	12.0827	122.0264	41	1	1	0	1
531	0.1055	0.4581	17.6016	136.7607	60	2	0	0	1
532	0.0927	0.3970	1.2682	104.7567	59	1	2	0	1
533	0.1921	0.1149	10.9201	152.3317	60	2	0	0	1
534	0.3468	0.3824	8.1105	122.1048	74	2	0	0	1
538	0.0340	0.1813	1.3437	73.5478	50	1	1	0	0
539	0.0612	0.1518	8.1131	136.1850	40	2	0	0	0
541	0.0464	0.1110	2.6580	103.3803	35	2	0	0	0
542	0.0733	0.0966	3.9429	112.1423	45	2	0	0	0
543	0.2162	0.5285	1.5268	74.0650	43	1	1	1	1
545	0.0272	0.1268	1.4009	83.7070	39	1	0	0	0
546	0.0368	0.6221	1.7305	89.7725	42	1	1	0	1
547	0.0076	0.3702	9.3639	67.2512	42	2	0	0	0
548	0.0076	0.2256	3.0528	46.2361	50	2	0	0	0
549	0.0179	0.4184	1.6895	86.5896	45	1	0	0	1
551	0.0076	0.4885	3.2091	128.7563	48	1	1	0	1
552	0.0824	0.6305	1.1763	86.7887	40	1	0	1	1
553	0.0076	0.3125	2.6838	106.5212	38	1	1	2	1

No.	Mo	Se	Sr	Zn	Age	Gender	moking statu	rinking Statu	Arsenism
554	0.0651	0.4559	2.0525	85.7543	48	1	2	2	1
556	0.0964	0.3346	1.7507	100.2201	60	1	1	1	1
558	0.0144	0.4407	0.8994	80.3257	30	1	0	2	0
559	0.0076	0.2372	1.1038	35.9294	49	1	1	0	1
560	0.0045	0.2960	1.7735	94.4047	47	1	0	1	1
561	0.0777	0.5866	6.5756	67.6727	65	1	2	0	1
565	0.0987	0.1729	3.0620	111.5016	58	2	0	0	1
702	0.0076	1.0121	0.0737	20.5273	42	2	0	0	0
703	0.1386	0.5136	58.7892	101.4025	33	2	0	0	0
704	0.1205	0.5098	11.4596	178.6092	38	2	0	0	0
705	0.0912	0.5091	25.5139	119.1739	44	2	0	0	0
706	0.0300	0.5039	44.3677	89.7036	34	2	0	1	0
709	0.0744	0.6946	48.5830	166.5603	46	2	0	0	0
710	0.0989	0.6926	94.1063	101.8689	33	2	0	0	0
711	0.1474	0.6804	7.9200	130.8992	32	1	1	1	0
712	0.1844	0.6797	84.6253	306.0028	29	2	0	0	0
713	0.2205	0.6739	8.1286	143.8651	40	1	1	1	0
722	0.0021	0.6693	1.1468	115.6875	45	2	1	0	0
723	0.0887	0.6530	20.5240	368.6389	29	2	0	0	0
724	0.0502	0.6418	29.6816	237.2789	36	2	0	0	0
725	0.1468	0.6411	6.2079	120.7431	60	2	0	0	0
726	0.1073	0.6392	9.9484	127.9593	36	2	0	0	0
727	0.1046	0.6285	7.1255	213.0155	38	1	1	1	0
728	0.2954	0.6273	40.2291	521.2354	38	2	0	0	0
729	0.2100	0.6191	3.7221	117.9350	41	1	1	0	0
730	0.2090	0.6181	22.3937	193.4509	44	1	1	1	0
731	0.2116	1.3979	3.3209	147.8496	51	1	1	0	0
734	0.1884	0.3764	25.6772	67.0730	51	2	0	0	0
735	0.1416	0.6146	39.5280	308.8637	48	2	0	0	0
739	0.2518	0.6882	40.2707	148.9735	58	2	0	0	0
740	0.1325	0.6062	5.5133	93.3376	36	1	2	0	0
742	0.1938	0.4728	14.6183	109.1350	55	2	0	0	0
744	0.1779	0.5938	35.6434	601.2195	36	2	0	0	0
745	0.1067	0.5808	2.8712	101.2137	30	1	1	0	0
746	0.2609	0.5784	26.5370	90.2080	42	2	0	0	0
747	0.0076	0.5647	38.6392	235.9210	28	2	0	0	0
748	0.1392	0.7449	121.9399	126.7124	34	2	0	0	0
755	0.0559	0.5615	60.2644	354.3082	36	2	0	0	0

No.	Mo	Se	Sr	Zn	Age	Gender	moking statu	rinking Statu	Arsenism
760	0.2548	0.5567	18.2540	241.2340	45	2	0	0	0
761	0.3246	0.5565	16.1548	86.1433	55	2	0	0	0
762	0.1589	0.3435	74.0761	95.3612	51	2	0	0	0
766	0.0901	0.5536	29.0880	429.7318	30	2	0	0	0
767	0.2836	0.5419	46.0973	538.8050	36	2	0	0	0
768	0.1658	0.5385	15.4895	116.7790	58	2	0	0	0
769	0.0554	0.5348	2.3668	114.8077	38	2	0	0	0
775	0.0076	0.5253	0.2000	30.9321	32	1	1	1	0
777	0.0641	0.5147	1.7519	107.4168	42	1	1	1	0
780	0.0343	0.5047	7.8722	101.6657	36	2	0	0	0
781	0.2015	0.4893	45.1160	141.9065	50	2	0	0	0
782	28.3752	0.4715	15.7622	177.5771	39	2	0	0	0
785	0.1246	0.4564	22.2503	136.2985	32	2	0	0	0
787	0.1629	0.4559	42.0615	107.5400	30	2	0	0	0
788	0.1084	0.4530	39.7717	78.8169	38	2	0	0	0
789	0.1945	0.4376	24.1462	127.8446	59	2	0	0	0
790	0.1557	0.4098	29.1926	153.0503	58	2	0	0	0
791	0.1572	0.4078	6.2312	115.6021	58	2	0	0	0
794	0.1852	0.3718	46.8537	106.3370	41	2	0	0	0
795	0.0171	0.3699	66.1630	133.6309	42	2	0	0	0
799	0.1722	0.3523	8.6325	101.9643	23	1	1	1	0
800	0.1571	0.3184	3.5223	138.3146	28	1	1	2	0
801	0.1913	0.3064	18.3030	115.6957	27	1	1	1	0

No.	Intervention	Group	Al	As	Cd	Hg	Li	Pb	Ca	K	Mg	Na
2	1	1	30.716	0.528	0.100	0.066	0.018	1.175	469.929	130.300	52.878	63.687
7	1	1	35.627	0.728	0.123	0.014	0.024	1.212	402.541	142.357	141.799	162.066
8	1	1	11.553	0.255	0.209	0.079	0.121	7.899	588.664	134.270	78.520	306.600
9	1	1	66.324	0.219	0.317	0.043	0.069	1.917	374.068	97.543	78.200	236.747
17	1	1	32.146	0.220	0.199	0.064	0.183	1.199	638.870	297.111	161.205	143.899
19	1	1	90.629	0.210	0.844	0.086	0.045	0.564	601.996	178.557	129.647	77.814
20	1	1	31.584	0.321	0.188	0.017	0.016	0.463	518.558	274.899	146.205	621.287
31	1	1	17.694	0.290	0.183	0.057	0.109	2.656	462.681	167.066	226.724	206.963
36	1	1	113.024	0.233	0.184	0.081	0.094	0.512	562.068	141.234	148.983	43.815
37	1	1	15.283	0.347	0.554	0.010	0.018	0.652	980.222	176.282	152.654	282.639
43	1	1	16.671	0.712	0.613	0.013	0.022	0.543	1139.757	209.391	151.838	203.769
44	1	1	23.169	0.785	0.239	0.056	0.057	0.699	1019.511	129.100	135.606	56.362
56	1	1	42.203	0.328	0.294	0.052	0.018	1.088	363.076	109.443	139.666	203.986
58	1	1										
61	1	1	32.040	0.443	0.201	0.016	0.010	0.555	741.929	122.457	118.818	280.416
63	1	1	28.755	0.457	0.459	0.070	0.078	1.299	993.245	213.749	144.126	512.295
64	1	1	56.520	0.229	0.221	0.011	0.016	0.509	504.169	114.103	167.571	102.104
67	1	1	36.797	0.212	0.322	0.036	0.029	0.645	375.663	118.755	68.839	96.349
68	1	1	84.580	0.679	0.336	0.059	0.027	1.022	388.777	196.409	90.510	99.303
69	1	1	23.265	1.103	0.780	0.013	0.018	1.362	564.276	87.787	158.659	102.342
71	1	1	32.923	0.210	0.181	0.023	0.032	1.711	400.208	69.923	55.931	418.885
72	1	1	69.551	0.253	0.754	0.047	0.013	4.031	527.022	157.403	61.210	186.404
77	1	1	20.330	0.643	0.440	0.022	0.080	3.077	675.823	451.699	78.807	188.788
79	1	1	33.935	0.609	0.207	0.019	0.034	0.876	252.189	151.508	137.730	81.894
90	1	1	15.835	0.549	0.207	0.038	0.030	1.042	695.677	797.922	168.635	96.346
93	1	1	24.643	0.391	0.141	0.027	0.014	1.138	443.947	230.264	181.472	186.074
97	1	1	14.268	0.563	0.211	0.011	0.030	0.832	565.506	125.586	77.306	57.551
98	1	1										
102	1	1	22.450	0.203	0.331	0.064	0.015	1.235	497.426	200.188	70.523	77.542
104	1	1	15.353	0.313	0.933	0.145	0.086	0.454	180.128	207.347	232.036	177.465
108	1	1	73.165	0.300	0.290	0.051	0.025	0.813	882.879	280.967	111.666	380.158
111	1	1	34.698	0.519	0.290	0.015	0.155	2.806	1187.447	240.330	152.335	220.475
112	1	1	102.294	0.351	0.526	0.051	0.016	1.538	343.752	139.743	151.949	150.035
116	1	1	73.394	0.228	0.262	0.021	0.026	0.888	334.608	74.342	76.865	177.951
124	1	1	21.794	0.242	0.369	0.026	0.017	0.793	780.643	409.332	228.789	362.748
125	1	1	30.228	0.196	0.080	0.026	0.045	0.429	557.856	117.199	112.802	61.190
129	1	1	12.793	0.268	0.236	0.052	0.171	0.665	506.370	151.314	181.172	218.718
145	1	1	27.049	0.586	0.481	0.071	0.065	0.482	1156.691	128.821	167.497	111.961

No.	Intervention	Group	Al	As	Cd	Hg	Li	Pb	Ca	K	Mg	Na
147	1	1										
149	1	1	17.658	0.355	0.787	0.091	0.029	0.455	810.832	73.213	98.075	126.799
152	1	1	31.045	0.315	0.516	0.039	0.117	2.939	594.456	513.420	82.154	322.068
154	1	1	11.363	0.381	0.476	0.068	0.041	2.440	603.697	212.706	147.223	36.019
159	1	1	10.807	0.279	0.876	0.028	0.061	0.929	312.870	141.124	72.182	63.815
160	1	1	37.787	0.215	0.195	0.047	0.055	1.139	260.124	68.744	160.451	71.043
161	1	1	15.068	0.288	0.498	0.027	0.068	1.147	791.072	125.775	191.627	95.846
162	1	1	28.684	0.211	0.191	0.016	0.008	0.682	220.541	79.968	77.591	83.321
3	1	2	38.303	0.207	0.291	0.088	0.058	0.373	997.622	266.957	110.932	263.479
10	1	2	36.298	0.388	0.172	0.032	0.013	1.064	703.857	180.173	142.512	279.099
11	1	2	41.555	0.606	0.330	0.074	0.032	0.764	762.799	214.816	78.576	183.501
16	1	2	17.423	0.341	0.493	0.049	0.032	1.185	291.514	325.995	214.560	267.021
18	1	2	60.689	0.153	0.280	0.015	0.074	0.825	892.619	105.785	81.963	143.038
21	1	2	43.985	0.615	0.248	0.054	0.025	0.738	779.093	109.881	97.497	347.182
24	1	2	12.428	0.520	0.327	0.077	0.019	1.059	424.564	84.335	130.753	62.834
25	1	2	52.063	0.298	0.126	0.037	0.072	0.654	480.339	132.299	124.491	42.949
28	1	2	48.845	0.379	0.271	0.047	0.060	1.914	320.934	445.943	138.205	296.597
32	1	2	32.805	0.522	0.129	0.023	0.031	0.809	383.485	223.478	125.525	135.346
33	1	2										
41	1	2	30.453	0.237	0.306	0.028	0.049	0.764	481.551	372.695	124.631	152.155
59	1	2	31.935	0.342	0.215	0.048	0.086	0.933	1094.872	172.610	184.221	118.328
60	1	2	19.981	0.411	0.228	0.090	0.017	3.910	777.846	73.800	72.521	144.943
62	1	2	32.820	1.506	0.359	0.015	0.058	1.365	1113.934	48.017	240.521	77.603
65	1	2	29.249	0.756	0.582	0.093	0.006	1.754	525.496	107.121	161.355	138.761
70	1	2	28.090	0.465	1.266	0.072	0.035	1.410	385.559	147.584	140.111	226.457
73	1	2	51.998	0.018	0.197	0.027	0.092	0.223	694.573	111.205	143.525	48.057
74	1	2	26.489	0.540	1.064	0.074	0.058	0.599	680.555	563.690	167.104	278.775
78	1	2	27.877	0.332	0.175	0.104	0.028	0.331	390.204	56.626	213.983	60.000
80	1	2	23.008	0.420	0.267	0.041	0.058	1.506	691.137	286.134	177.016	753.664
82	1	2	25.765	0.530	0.406	0.043	0.098	2.146	385.306	91.063	144.172	191.670
83	1	2	23.092	0.664	0.224	0.027	0.042	1.424	325.731	129.301	118.929	118.373
88	1	2	18.244	0.579	0.193	0.098	0.043	0.338	639.035	107.460	161.165	191.677
95	1	2	40.519	0.185	0.219	0.096	0.041	0.660	297.220	159.679	149.199	293.891
99	1	2	24.812	0.239	0.200	0.022	0.024	0.878	384.838	57.351	136.068	79.733
103	1	2	29.815	0.709	0.146	0.032	0.025	0.348	615.025	148.180	153.616	140.921
105	1	2	21.283	0.216	1.205	0.010	0.028	0.582	904.880	92.330	174.055	133.549
107	1	2	14.415	0.314	0.174	0.026	0.064	1.492	756.406	171.280	92.948	129.414
109	1	2	37.777	0.829	1.281	0.040	0.057	1.033	1065.745	813.359	347.331	385.795

No.	Intervention	Group	Al	As	Cd	Hg	Li	Pb	Ca	K	Mg	Na
110	1	2	49.200	0.315	0.312	0.085	0.078	1.127	298.711	52.051	118.459	74.240
123	1	2	32.930	0.226	0.375	0.065	0.025	0.885	398.398	148.907	120.471	425.777
126	1	2	73.237	0.627	0.341	0.035	0.010	0.352	323.553	287.640	125.991	354.415
127	1	2	24.185	0.242	0.473	0.064	0.065	0.907	816.343	160.285	199.371	94.779
130	1	2	32.381	0.241	0.167	0.079	0.058	0.889	484.767	135.706	154.356	204.481
133	1	2	20.186	0.190	0.173	0.110	0.087	0.430	492.048	45.518	140.141	32.781
135	1	2										
138	1	2	61.592	0.455	0.307	0.029	0.094	0.691	234.218	897.792	111.420	314.794
143	1	2	29.896	0.261	0.274	0.020	0.028	2.529	588.070	41.987	73.852	22.638
144	1	2	39.172	0.297	0.162	0.047	0.064	2.363	382.185	42.282	141.107	23.182
148	1	2	13.249	0.694	0.609	0.060	0.087	1.622	499.892	110.806	165.486	391.501
151	1	2	34.806	0.572	0.396	0.030	0.019	0.851	122.146	167.671	119.997	136.165
157	1	2	30.726	0.427	0.253	0.067	0.028	0.574	388.735	60.586	129.525	79.687
2	2	3	24.497	0.716	0.194	0.159	0.044	1.658	1103.391	42.649	114.874	102.113
7	2	3	37.029	0.536	0.265	0.036	0.019	0.436	416.237	87.012	180.684	144.712
8	2	3	56.448	0.322	0.249	0.323	0.028	0.671	241.932	197.664	127.912	222.187
9	2	3	4.999	0.592	0.086	0.046	0.041	1.717	637.234	136.981	112.241	69.989
17	2	3	76.463	0.312	0.324	0.038	0.049	0.911	491.991	34.448	61.935	93.193
19	2	3	30.265	0.146	0.478	0.139	0.055	0.777	501.066	174.886	172.958	214.263
20	2	3	25.988	0.427	0.162	0.065	0.007	1.149	234.589	388.716	233.108	61.333
31	2	3	35.241	0.733	0.490	0.023	0.066	1.379	313.237	187.209	235.928	318.314
36	2	3	36.289	0.354	0.474	0.037	0.071	0.636	1208.449	76.357	237.724	133.083
37	2	3	41.416	0.381	1.634	0.120	0.038	1.104	566.065	57.240	223.634	123.807
43	2	3	21.659	0.278	0.39	0.119	0.014	1.018	813.462	146.652	143.623	59.635
44	2	3	11.928	0.222	0.178	0.034	0.077	0.850	549.730	42.915	140.810	56.929
56	2	3	33.729	0.271	0.167	0.023	0.230	1.688	723.124	432.227	147.989	138.459
58	2	3	36.856	0.135	0.271	0.225	0.055	0.474	312.063	75.597	145.717	280.647
61	2	3	5.057	0.396	0.230	0.228	0.059	0.993	1079.743	286.181	93.037	136.688
63	2	3	26.786	1.111	0.658	0.035	0.061	2.385	1810.599	158.192	97.198	223.542
64	2	3	11.621	0.192	0.323	0.020	0.045	1.175	1111.061	224.759	116.181	76.671
67	2	3	8.420	0.194	0.571	0.035	0.249	0.989	644.393	33.038	151.690	117.333
68	2	3	18.129	0.211	0.273	0.037	0.241	2.373	1173.584	39.907	144.319	149.789
69	2	3	11.718	0.109	0.136	0.029	0.067	0.958	595.319	12.889	187.678	135.361
71	2	3	5.921	0.246	1.023	0.090	0.038	1.260	521.193	414.744	71.715	434.350
72	2	3	18.340	0.229	1.849	0.081	0.055	4.891	899.563	101.821	179.134	44.551
77	2	3	55.518	0.259	0.388	0.032	0.046	3.235	743.725	191.519	112.949	231.554
79	2	3	89.081	0.577	0.496	0.056	0.054	0.935	516.328	79.512	74.601	75.592
90	2	3										

No.	Intervention	Group	Al	As	Cd	Hg	Li	Pb	Ca	K	Mg	Na
93	2	3	43.396	0.345	0.158	0.030	0.010	0.747	661.120	16.606	159.931	150.543
97	2	3	5.983	0.737	0.151	0.019	0.039	0.581	716.851	31.211	57.141	92.273
98	2	3	39.303	0.440	0.514	0.030	0.057	0.591	339.471	150.078	137.154	214.525
102	2	3	53.504	0.747	0.449	0.037	0.019	0.673	379.678	64.970	145.451	184.571
104	2	3	16.172	0.259	0.143	0.046	0.022	1.753	596.582	684.537	74.932	386.249
108	2	3	37.852	0.297	0.532	0.028	0.030	1.137	533.887	562.611	80.168	321.586
111	2	3	31.149	0.407	0.360	0.101	0.011	4.205	607.497	242.324	177.943	343.748
112	2	3	59.184	0.390	0.903	0.124	0.008	1.611	407.118	410.043	152.524	284.829
116	2	3	16.969	0.399	0.244	0.332	0.006	0.490	266.100	286.746	152.567	96.898
124	2	3	63.792	0.710	0.386	0.033	0.011	3.075	300.953	131.807	142.165	297.571
125	2	3	69.807	0.251	0.641	0.049	0.014	0.708	405.872	168.686	100.536	170.787
129	2	3	55.039	0.169	0.252	0.021	0.017	0.549	378.874	13.033	81.000	171.578
145	2	3	55.354	0.238	0.235	0.026	0.010	1.945	434.659	254.843	92.968	379.144
147	2	3	44.143	0.485	0.131	0.076	0.031	2.535	866.505	8.462	149.304	43.621
149	2	3										
152	2	3	29.725	0.526	1.841	0.270	0.013	2.120	326.771	380.358	137.256	176.399
154	2	3	22.612	0.238	0.337	0.166	0.014	0.895	512.295	375.492	225.728	316.155
159	2	3										
160	2	3	13.869	0.202	0.249	0.043	0.010	1.941	841.675	32.404	157.089	234.657
161	2	3										
162	2	3	3.086	0.406	0.124	0.019	0.020	0.683	878.359	64.851	220.745	118.606
3	2	4	10.953	0.250	0.334	0.024	0.037	0.918	828.034	487.017	240.259	502.870
10	2	4	14.024	0.206	0.330	0.021	0.032	1.441	22.240	9.784	155.222	175.750
11	2	4	10.107	0.309	0.388	0.043	0.106	1.969	378.226	1062.593	134.480	1281.810
16	2	4	14.569	0.439	0.344	0.034	0.038	0.611	1683.275	566.205	116.311	535.631
18	2	4	12.704	0.188	0.341	0.039	0.027	1.194	1345.640	33.581	166.481	135.467
21	2	4	12.853	0.436	0.340	0.026	0.041	0.497	20.291	113.590	164.141	57.286
24	2	4	6.357	0.202	0.337	0.043	0.116	1.137	656.288	56.294	98.174	129.629
25	2	4	12.178	0.318	0.346	0.047	0.035	0.552	81.436	405.958	163.438	950.359
28	2	4	14.879	0.183	0.324	0.028	0.052	0.903	557.831	87.051	267.206	137.197
32	2	4	18.496	0.462	0.347	0.030	0.068	0.252	1911.234	560.427	88.238	528.652
33	2	4	11.972	0.282	0.346	0.055	0.044	1.120	644.478	330.611	104.977	309.346
41	2	4	30.453	0.206	0.324	0.039	0.120	1.016	587.017	120.832	136.907	68.821
59	2	4	18.698	0.110	0.334	0.028	0.114	0.752	496.329	99.592	149.000	45.951
60	2	4	9.489	0.314	0.336	0.039	0.039	0.886	129.242	409.988	243.267	1052.473
62	2	4	8.145	0.146	0.346	0.057	0.067	0.745	128.611	36.739	81.118	162.674
65	2	4	10.277	0.245	0.333	0.038	0.048	1.581	52.053	208.111	50.435	535.347
70	2	4	12.520	0.198	0.335	0.097	0.055	1.663	531.497	434.981	168.154	428.264

No.	Intervention	Group	Al	As	Cd	Hg	Li	Pb	Ca	K	Mg	Na
73	2	4	33.739	0.132	0.325	0.051	0.028	0.737	1054.475	82.767	169.431	52.416
74	2	4	6.440	0.411	0.343	0.024	0.037	0.599	793.846	443.905	355.807	834.396
78	2	4	9.723	0.355	0.345	0.057	0.039	0.631	0.577	268.143	123.909	241.087
80	2	4	12.765	0.170	0.327	0.046	0.062	1.163	790.334	27.990	166.797	44.502
82	2	4	10.667	0.154	0.370	0.036	0.063	0.366	372.399	34.048	91.045	52.460
83	2	4	12.426	0.275	0.363	0.034	0.031	1.454	470.841	173.734	76.382	471.071
88	2	4	11.356	0.353	0.329	0.035	0.028	0.728	466.312	58.638	93.162	5.216
95	2	4	16.169	0.282	0.360	0.037	0.033	1.327	402.876	74.941	72.493	200.626
99	2	4	4.236	0.248	0.353	0.045	0.039	1.745	661.227	176.133	72.260	424.182
103	2	4	15.133	0.215	0.399	0.026	0.028	1.064	53.972	85.523	312.406	25.820
105	2	4	24.336	0.126	0.336	0.030	0.023	0.904	3514.552	30.883	158.616	187.580
107	2	4	21.872	0.154	0.338	0.036	0.045	1.277	585.982	78.419	133.531	122.507
109	2	4	9.038	0.411	0.338	0.036	0.023	0.376	883.506	184.715	259.629	491.598
110	2	4	11.706	0.195	0.322	0.037	0.075	1.184	2002.584	124.887	135.892	57.812
123	2	4	19.683	0.360	0.394	0.059	0.058	0.847	679.684	876.407	163.141	2019.598
126	2	4	16.889	0.287	0.339	0.057	0.022	0.477	245.942	228.911	265.330	318.373
127	2	4	15.761	0.344	0.321	0.049	0.037	0.748	1338.289	23.402	210.625	123.678
130	2	4	13.292	0.185	0.327	0.039	0.026	0.787	1984.384	457.944	181.521	1273.301
133	2	4	28.457	0.247	0.386	0.055	0.054	0.695	599.318	168.815	85.160	36.152
135	2	4	17.323	0.310	0.347	0.061	0.048	0.278	391.669	129.013	126.992	41.348
138	2	4	21.907	0.207	0.333	0.047	0.048	0.200	73.458	160.755	222.501	66.098
143	2	4	13.440	0.288	0.327	0.073	0.038	1.307	773.432	232.929	74.448	228.240
144	2	4	7.049	0.269	0.336	0.076	0.025	1.236	385.257	80.405	149.588	145.110
148	2	4	10.757	0.130	0.388	0.041	0.029	0.639	2179.305	921.800	164.530	2154.549
151	2	4	13.136	0.081	0.324	0.024	0.040	0.116	180.949	58.014	382.129	39.706
157	2	4										

No.	P	B	Mn	Ni	V	Co	Cr	Cu	Fe	Mo	Se	Sr	Zn
2	168.408	0.614	0.971	0.436	0.047	0.034	1.707	6.068	220.934	0.124	0.377	9.437	58.749
7	132.051	0.872	0.230	0.130	0.010	0.018	1.842	6.106	226.434	0.151	0.403	10.981	64.638
8	299.122	4.524	1.424	0.703	0.098	0.030	3.175	9.559	180.466	0.685	0.388	11.901	91.669
9	170.848	1.194	2.121	0.152	0.043	0.039	2.604	13.394	252.138	0.366	0.271	3.238	52.977
17	97.671	1.555	0.853	1.365	0.040	0.193	1.608	6.449	325.962	0.153	0.395	11.929	265.352
19	87.054	0.527	1.115	0.310	0.049	0.018	1.766	9.840	23.909	0.200	0.289	4.907	41.451
20	154.203	1.265	1.177	0.071	0.035	0.035	3.315	11.998	456.795	0.170	0.360	4.694	87.144
31	92.746	2.284	1.084	0.159	0.023	0.014	1.974	5.588	230.264	0.080	0.440	14.695	74.093
36	155.137	1.697	0.610	0.611	0.033	0.117	2.419	2.614	131.412	0.224	0.276	8.003	109.783
37	206.859	1.264	0.839	0.078	0.025	0.043	2.215	20.366	125.572	0.698	0.520	10.926	88.531
43	101.278	0.110	0.360	0.137	0.016	0.014	1.401	8.107	158.177	0.054	0.352	7.825	59.177
44	166.936	1.499	1.141	0.460	0.014	0.051	2.676	7.176	150.277	0.456	0.388	8.659	99.309
56	164.642	1.336	5.572	0.297	0.017	0.076	3.034	8.408	198.687	1.178	0.381	16.319	83.468
58													
61	169.543	1.358	1.661	0.031	0.022	0.055	2.276	2.854	153.408	0.192	0.428	6.503	62.674
63	191.873	1.657	1.991	0.561	0.035	0.062	2.761	14.860	184.847	0.292	0.248	8.610	68.409
64	147.104	1.372	1.609	0.041	0.035	0.045	2.825	12.614	131.412	0.405	0.423	4.733	119.049
67	161.222	1.480	1.367	0.292	0.017	0.193	1.985	12.067	113.962	0.248	0.358	13.473	284.106
68	176.994	1.868	1.501	0.595	0.049	0.270	1.961	6.312	137.228	0.386	0.307	5.070	147.745
69	96.665	0.341	0.624	0.298	0.011	0.077	1.778	7.336	226.625	0.170	0.305	12.743	104.251
71	167.546	5.453	1.172	0.681	0.026	0.035	5.364	11.162	471.212	1.329	0.461	13.329	96.511
72	101.263	1.459	3.329	0.218	0.042	0.045	1.658	23.956	217.362	0.247	0.238	4.164	63.393
77	153.332	0.737	1.033	0.252	0.049	0.029	2.224	12.333	161.460	0.304	0.444	5.892	61.151
79	101.476	0.778	0.454	0.165	0.028	0.016	3.837	12.644	145.438	0.086	0.782	6.601	65.915
90	122.619	5.438	1.517	0.092	0.043	0.014	1.651	3.769	183.325	0.111	0.390	1.445	60.375
93	95.353	0.739	2.405	0.241	0.030	0.031	1.585	12.620	156.563	0.242	0.276	3.619	45.958
97	171.770	1.532	0.929	0.234	0.038	0.041	2.579	5.369	160.220	0.232	0.369	5.010	108.203
98													
102	160.482	1.574	2.006	0.464	0.032	0.073	2.828	12.617	184.300	0.317	0.258	2.650	113.809
104	123.614	2.998	0.556	0.129	0.025	0.019	1.647	22.833	149.025	0.051	0.438	5.919	76.960
108	219.867	1.445	1.907	0.342	0.037	0.037	2.085	13.011	170.619	0.193	0.338	4.989	80.619
111	180.213	1.030	1.766	0.210	0.036	0.038	2.468	12.574	89.098	0.229	0.280	11.525	58.267
112	177.250	0.902	5.160	0.068	0.024	0.052	2.868	12.911	161.365	0.295	0.314	12.273	80.147
116	246.838	1.305	1.164	0.369	0.058	0.078	3.021	12.922	150.390	0.636	0.299	11.343	110.609
124	111.725	2.269	0.685	0.190	0.030	0.013	1.708	5.914	195.680	0.107	0.450	3.108	76.510
125	101.893	0.298	0.694	0.103	0.016	0.113	1.546	20.135	117.091	0.079	0.335	1.890	61.745
129	186.953	0.967	1.402	0.786	0.045	0.034	2.893	10.299	125.860	0.182	0.312	6.140	97.273
145	99.634	0.320	0.306	0.091	0.014	0.100	1.566	10.293	179.465	0.061	0.403	8.519	66.068

No.	P	B	Mn	Ni	V	Co	Cr	Cu	Fe	Mo	Se	Sr	Zn
147													
149	80.006	0.211	2.100	0.270	0.022	0.018	1.391	12.119	175.197	0.085	0.211	9.597	37.320
152	177.798	1.143	1.632	0.670	0.046	0.058	2.478	9.677	184.380	0.636	0.329	2.966	91.116
154	215.474	1.250	2.995	0.754	0.017	0.049	4.927	5.194	193.733	0.207	0.263	11.532	84.097
159	94.334	1.405	0.899	0.454	0.012	0.113	1.513	8.796	156.544	0.110	0.310	3.562	73.743
160	99.839	0.304	1.509	0.175	0.040	0.029	1.961	13.025	125.848	0.353	0.332	2.914	70.653
161	121.912	1.963	1.634	0.694	0.026	0.023	1.715	6.771	174.827	0.080	0.276	5.473	99.977
162	116.702	0.933	0.293	0.224	0.020	0.030	1.858	3.826	113.127	0.113	0.394	7.921	72.055
3	109.752	2.891	2.743	0.509	0.015	0.064	2.141	10.238	52.274	0.180	0.413	13.240	54.738
10	112.796	3.736	1.971	0.123	0.027	0.059	1.671	7.920	44.425	0.130	0.503	10.641	51.088
11	137.287	2.541	2.497	0.258	0.031	0.048	3.943	8.391	56.879	0.139	0.356	6.606	76.677
16	192.433	1.112	1.148	0.431	0.009	0.046	3.042	10.408	196.326	0.667	0.245	7.062	81.477
18	175.009	1.587	1.028	0.035	0.039	0.034	2.208	9.821	201.156	0.334	0.255	12.882	75.985
21	207.534	0.687	2.216	0.141	0.059	0.040	2.197	13.910	163.842	0.254	0.536	9.582	76.411
24	118.482	1.592	1.189	0.272	0.028	0.037	2.249	10.371	156.042	0.882	0.764	4.425	109.122
25	174.144	1.401	1.198	0.281	0.199	0.045	2.668	10.064	249.328	0.200	0.619	5.699	63.225
28	156.515	2.649	0.641	0.244	0.030	0.009	2.064	8.860	55.224	0.191	0.467	7.116	88.106
32	116.424	2.789	2.841	0.115	0.035	0.117	1.863	8.246	54.166	0.185	0.281	3.585	49.691
33													
41	95.143	0.381	0.583	0.290	0.021	0.112	1.564	9.987	142.714	0.144	0.322	14.555	144.775
59	143.439	1.455	1.273	0.421	0.014	0.149	2.390	7.311	194.542	0.379	0.623	2.429	68.799
60	102.613	0.105	2.227	0.313	0.256	0.052	2.356	12.189	50.300	0.224	0.317	4.734	63.362
62	137.398	0.196	3.986	0.620	0.019	0.030	8.113	12.672	66.719	0.161	0.173	11.318	147.600
65	86.400	0.571	1.396	0.235	0.033	0.054	1.656	12.599	44.317	0.130	0.666	7.740	133.995
70	146.564	1.572	1.822	0.318	0.006	0.087	2.406	8.718	193.741	0.348	0.522	3.991	111.673
73	45.036	0.476	0.543	0.139	0.015	0.037	0.625	7.496	49.384	0.154	0.684	3.656	100.794
74	98.790	0.676	5.108	0.131	0.078	0.109	1.590	10.183	43.341	0.124	0.394	5.061	231.060
78	129.198	2.836	0.728	0.240	0.063	0.059	1.653	7.989	156.543	0.092	0.316	8.700	74.960
80	178.076	1.065	1.419	0.180	0.017	0.044	2.415	9.543	177.902	0.511	0.390	10.974	78.790
82	164.144	1.342	0.458	0.040	0.027	0.031	2.552	9.056	164.312	0.210	0.279	4.436	86.033
83	138.004	1.235	2.227	0.039	0.019	0.034	2.277	9.825	175.421	0.096	0.426	5.044	48.784
88	194.673	2.689	0.665	0.024	0.042	0.015	1.889	9.736	141.409	0.078	0.534	6.024	60.462
95	145.712	1.286	1.076	0.144	0.020	0.028	2.156	12.388	259.581	0.203	0.179	6.381	54.315
99	218.719	3.527	0.699	0.091	0.026	0.045	2.867	10.972	202.747	0.154	0.275	8.300	81.884
103	159.907	1.166	2.079	0.080	0.035	0.035	1.895	10.397	109.098	0.158	0.717	11.793	72.506
105	172.039	1.240	2.118	0.493	0.033	0.031	2.201	10.769	173.292	0.255	0.282	6.116	76.211
107	99.157	2.872	1.525	0.121	0.013	0.136	1.470	11.763	42.632	0.136	0.357	3.621	139.664
109	116.426	1.590	4.740	0.493	0.035	0.037	2.054	10.258	363.153	1.364	0.783	10.880	128.049

No.	P	B	Mn	Ni	V	Co	Cr	Cu	Fe	Mo	Se	Sr	Zn
110	142.819	1.480	0.829	0.225	0.012	0.037	2.345	9.532	172.785	0.221	0.267	22.575	74.738
123	171.354	0.738	2.914	0.388	0.060	0.039	2.622	8.709	171.573	0.347	0.545	4.641	74.846
126	181.657	0.551	5.323	0.085	0.023	0.029	3.189	8.195	145.903	0.539	0.321	13.031	80.377
127	151.178	1.504	3.207	0.395	0.062	0.020	1.955	8.911	154.373	0.258	0.238	3.007	64.709
130	90.482	0.311	4.058	0.438	0.031	0.029	1.964	10.341	177.902	0.241	0.283	3.949	155.232
133	107.822	1.992	1.832	0.174	0.019	0.192	1.327	9.506	150.569	0.144	0.237	11.424	62.343
135													
138	188.593	0.759	1.579	0.045	0.008	0.024	1.986	10.795	192.090	0.152	0.320	3.572	49.639
143	192.388	1.435	3.098	0.042	0.050	0.042	2.875	11.076	155.618	0.178	0.353	10.483	100.985
144	159.533	1.298	0.931	0.169	0.033	0.028	2.510	10.227	161.946	0.200	0.303	3.768	195.334
148	157.996	1.205	0.639	0.129	0.006	0.116	2.395	8.685	178.406	0.415	0.330	12.397	60.793
151	170.540	1.313	0.379	0.062	0.012	0.027	2.066	7.640	187.810	0.254	0.494	7.316	77.573
157	167.371	1.454	1.472	0.241	0.012	0.034	2.217	9.628	173.852	0.238	0.242	4.216	84.514
2	124.357	1.670	1.265	0.114	0.037	0.056	2.175	8.836	171.082	0.332	0.345	6.419	76.895
7	162.121	0.541	0.616	0.463	0.030	0.018	2.015	11.271	181.218	0.264	0.271	3.054	69.714
8	172.976	1.370	2.899	0.076	0.054	0.044	2.859	9.283	375.752	0.503	0.375	3.745	79.464
9	149.241	1.406	1.245	0.502	0.028	0.043	2.008	10.796	99.448	0.313	0.222	7.705	97.772
17	106.422	1.360	0.636	0.336	0.031	0.176	1.344	6.667	243.138	0.213	0.249	5.413	337.929
19	160.008	1.347	1.226	0.579	0.037	0.015	2.109	10.793	159.355	0.451	0.316	4.376	61.014
20	151.535	0.537	2.744	0.412	0.047	0.005	1.421	7.404	150.572	0.388	0.306	8.092	54.004
31	163.545	1.491	1.005	0.467	0.058	0.008	3.263	8.342	168.855	0.190	0.358	3.517	87.525
36	178.847	1.490	0.639	0.162	0.034	0.007	2.156	11.256	159.825	0.454	0.289	8.429	87.955
37	160.371	2.170	0.890	0.077	0.028	0.004	1.893	7.732	162.085	0.240	0.303	10.677	69.555
43	79.631	0.311	0.676	0.273	0.021	0.016	2.321	6.0653	227.698	0.018	0.314	10.966	78.326
44	178.136	1.584	0.944	0.255	0.042	0.004	3.344	10.253	92.539	0.009	0.306	5.265	79.408
56	120.221	1.260	1.062	0.385	0.047	0.037	2.219	10.321	101.760	0.418	0.338	10.983	62.887
58	150.645	1.396	0.491	0.100	0.046	0.022	2.229	8.840	267.814	0.161	0.252	6.652	72.891
61	151.228	1.064	1.309	0.247	0.058	0.027	2.720	9.135	192.101	0.082	0.303	5.322	59.675
63	213.645	1.357	3.413	0.561	0.038	0.091	3.807	31.721	119.989	0.302	0.264	3.055	59.367
64	176.940	1.357	2.391	0.071	0.031	0.065	7.967	9.864	115.850	0.014	0.334	4.652	123.303
67	211.713	3.604	1.132	0.438	0.016	0.141	1.902	10.588	78.830	0.141	0.264	5.365	126.060
68	174.251	1.066	3.436	0.283	0.037	0.092	2.463	22.828	95.857	0.499	0.353	4.366	277.624
69	168.715	0.916	1.041	0.032	0.048	0.039	3.413	12.151	196.071	0.286	0.311	4.633	192.363
71	184.743	1.263	0.952	0.382	0.037	0.019	2.478	9.815	95.945	0.116	0.341	7.137	68.041
72	175.127	1.000	3.866	0.113	0.060	0.027	2.968	15.742	103.968	0.281	0.218	10.366	62.326
77	146.224	1.528	1.914	0.375	0.075	0.025	1.981	9.176	289.118	0.505	0.286	8.699	97.143
79	178.542	0.662	0.838	0.051	0.029	0.022	1.968	9.017	165.568	0.133	0.439	6.235	72.282
90													

No.	P	B	Mn	Ni	V	Co	Cr	Cu	Fe	Mo	Se	Sr	Zn
93	117.820	1.204	1.666	0.132	0.027	0.114	1.707	8.155	153.917	0.048	0.129	9.021	63.951
97	116.381	2.379	0.060	0.485	0.038	0.006	1.137	11.599	148.091	0.040	0.214	8.636	90.592
98	109.017	2.327	0.151	0.111	0.055	0.007	1.022	10.768	263.280	0.050	0.272	10.997	91.951
102	94.781	0.746	1.533	0.260	0.019	0.033	1.340	6.621	156.615	0.211	0.179	6.436	82.797
104	145.107	3.147	1.812	0.529	0.020	0.016	1.662	10.596	371.466	1.096	0.288	5.954	56.513
108	185.409	0.834	1.241	0.340	0.027	0.018	2.404	11.771	102.317	0.134	0.377	7.587	77.198
111	161.277	0.532	3.773	0.074	0.037	0.067	2.866	12.289	108.131	0.468	0.309	7.127	50.987
112	159.152	1.075	3.670	0.182	0.032	0.034	1.769	10.853	184.919	0.156	0.277	6.257	58.944
116	174.528	1.310	0.355	0.144	0.023	0.076	1.991	9.307	248.415	0.416	0.317	10.287	85.467
124	164.844	1.202	1.237	0.186	0.027	0.032	1.485	11.127	256.758	0.072	0.426	10.613	80.602
125	136.238	1.161	0.752	0.218	0.035	0.092	2.051	9.438	159.940	0.525	0.348	6.067	128.733
129	140.814	1.237	0.453	0.230	0.027	0.041	1.258	13.405	154.129	0.032	0.363	8.645	155.187
145	183.214	1.497	2.257	0.262	0.047	0.094	3.111	11.927	95.586	0.167	0.524	4.615	58.948
147	139.389	3.071	3.797	0.270	0.034	0.014	1.306	9.916	249.236	0.043	0.292	5.883	75.971
149													
152	118.298	0.466	1.264	0.244	0.041	0.091	1.536	11.880	162.672	0.297	0.353	4.161	52.859
154	119.573	1.410	0.437	0.082	0.044	0.003	1.315	9.976	149.281	0.019	0.286	4.216	48.842
159													
160	100.319	1.512	3.066	0.333	0.022	0.131	2.620	10.833	276.368	0.759	0.272	3.113	53.043
161													
162	123.821	0.585	0.692	0.202	0.019	0.065	2.395	9.728	255.648	0.671	0.305	4.231	49.409
3	97.497	1.591	8.146	2.636	0.018	0.035	2.134	8.074	46.662	0.057	0.663	8.457	173.164
10	143.344	1.741	1.961	0.392	0.057	0.037	2.749	7.199	54.459	0.228	0.651	11.049	104.295
11	186.983	1.288	6.081	1.071	0.035	0.076	2.657	7.032	99.623	0.204	0.377	12.025	157.609
16	184.064	1.547	5.196	0.484	0.058	0.086	3.954	9.061	36.458	0.625	0.342	12.121	89.309
18	189.434	1.528	2.958	0.298	0.026	0.049	2.356	10.324	30.269	0.170	0.900	8.225	90.345
21	181.897	1.209	0.258	0.391	0.017	0.066	2.909	6.659	46.219	0.202	0.358	13.483	166.750
24	171.059	0.868	1.282	0.540	0.043	0.088	3.398	5.510	50.050	0.731	0.530	10.333	159.962
25	219.043	1.197	5.310	0.240	0.031	0.018	2.862	9.253	40.082	0.408	0.493	12.110	81.988
28	136.569	0.919	4.927	0.197	0.024	0.028	2.283	3.514	17.124	0.101	0.899	9.355	277.829
32	197.414	1.089	5.215	0.068	0.068	0.011	2.655	1.196	35.299	0.116	0.444	18.432	170.152
33	151.365	1.099	0.836	0.111	0.067	0.071	4.032	2.837	38.933	0.370	0.416	11.363	178.969
41	182.011	1.472	3.891	0.288	0.015	0.083	3.676	3.581	53.920	0.256	0.543	13.016	178.200
59	164.886	1.439	4.055	0.328	0.044	0.225	3.169	9.181	50.075	0.308	0.234	6.202	373.289
60	112.332	1.192	0.493	0.282	0.037	0.016	2.265	6.402	59.568	0.391	0.372	5.855	103.569
62	163.750	1.724	4.139	0.151	0.005	0.055	2.618	5.477	35.475	0.110	0.932	8.699	80.584
65	165.024	1.682	1.829	0.002	0.024	0.023	3.488	6.276	65.870	0.301	0.520	7.303	78.593
70	174.812	1.479	3.020	0.568	0.038	0.033	4.201	7.221	74.842	0.086	0.742	13.366	177.484

No.	P	B	Mn	Ni	V	Co	Cr	Cu	Fe	Mo	Se	Sr	Zn
73	176.050	1.621	1.132	0.142	0.168	0.069	2.101	8.834	42.571	0.038	0.620	13.662	86.634
74	165.343	1.030	5.054	0.154	0.031	0.023	2.951	3.693	72.072	0.665	0.419	14.303	90.846
78	195.355	1.947	3.294	0.214	0.025	0.031	4.489	8.656	60.778	0.125	0.711	13.525	109.313
80	159.504	0.604	4.057	0.212	0.067	0.017	3.038	1.979	67.314	0.219	0.742	8.377	81.515
82	125.817	0.257	1.777	0.262	0.040	0.069	3.152	3.904	32.572	0.296	0.880	7.054	174.678
83	77.848	0.885	2.182	0.337	0.008	0.025	10.174	8.714	46.398	0.427	0.731	6.352	265.842
88	112.749	0.392	0.241	0.521	0.020	0.037	2.187	2.003	28.908	0.354	0.331	5.033	181.833
95	108.171	0.636	4.224	2.159	0.113	0.027	2.012	4.158	59.127	0.105	1.166	21.364	166.003
99	145.151	0.300	4.965	0.568	0.025	0.055	2.553	10.095	53.591	0.065	0.659	8.216	99.014
103	143.991	2.232	6.312	0.088	0.024	0.050	2.639	5.743	45.807	0.426	0.864	5.323	97.307
105	134.839	1.061	2.153	0.112	0.049	0.017	2.369	3.109	40.123	0.507	0.668	13.095	73.928
107	151.032	0.850	5.923	0.308	0.022	0.039	3.545	8.382	74.468	0.415	0.779	10.625	115.229
109	175.497	1.590	4.847	0.083	0.055	0.017	2.932	2.701	53.122	0.149	0.363	13.652	100.099
110	156.089	1.016	5.676	0.091	0.060	0.036	3.796	5.076	62.044	0.422	0.361	16.557	165.784
123	144.865	1.914	0.012	0.142	0.073	0.051	2.229	5.939	35.241	0.412	0.576	15.985	181.814
126	113.882	0.601	0.850	2.539	0.045	0.116	7.436	4.154	55.266	0.876	0.291	11.831	200.705
127	171.542	1.670	2.421	2.757	0.046	0.023	2.516	5.884	56.642	0.143	0.533	13.128	119.464
130	166.234	0.784	2.525	0.114	0.030	0.068	2.151	3.979	40.269	0.029	0.381	4.582	79.880
133	178.903	1.620	5.921	0.056	0.047	0.035	2.096	3.993	35.122	0.022	0.442	20.430	94.668
135	195.751	0.966	3.003	1.989	0.049	0.027	3.907	6.781	46.946	0.896	0.526	5.627	136.792
138	201.039	1.852	5.881	0.191	0.054	0.051	2.062	5.159	40.946	0.060	0.365	12.199	170.550
143	192.838	0.827	3.470	0.008	0.060	0.037	2.492	5.868	26.554	0.029	0.422	13.099	97.648
144	163.304	0.603	2.555	0.061	0.043	0.023	2.357	8.501	34.350	0.041	0.497	13.481	99.816
148	162.226	0.612	1.667	0.389	0.074	0.115	2.541	5.780	34.620	0.113	0.497	7.560	87.153
151	5.591	0.257	1.488	0.207	0.023	0.027	0.144	9.331	33.304	0.019	0.550	10.150	78.652
157													
